# An atlas of the tomato epigenome reveals that KRYPTONITE shapes TAD-like boundaries through the control of H3K9ac distribution

**DOI:** 10.1073/pnas.2400737121

**Published:** 2024-07-05

**Authors:** Jing An, Rim Brik Chaouche, Leonardo I. Pereyra-Bistraín, Hugo Zalzalé, Qingyi Wang, Ying Huang, Xiaoning He, Chloé Dias Lopes, Javier Antunez-Sanchez, Catherine Bergounioux, Claire Boulogne, Cynthia Dupas, Cynthia Gillet, José Manuel Pérez-Pérez, Olivier Mathieu, Nicolas Bouché, Sotirios Fragkostefanakis, Yijing Zhang, Shaojian Zheng, Martin Crespi, Magdy M. Mahfouz, Federico Ariel, Jose Gutierrez-Marcos, Cécile Raynaud, David Latrasse, Moussa Benhamed

**Affiliations:** ^a^Université Paris-Saclay, CNRS, INRAE, Université Evry, Institute of Plant Sciences Paris-Saclay (IPS2), Orsay 91405, France; ^b^Université de Paris Cité, Institute of Plant Sciences Paris-Saclay (IPS2), Gif-sur-Yvette F-91190, France; ^c^School of Life Science, University of Warwick, Coventry CV4 7AL, United Kingdom; ^d^Imagerie-Gif, Electron Microscopy Facility, Institute for Integrative Biology of the Cell (I2BC), CEA, CNRS, Université Paris-Sud, Université Paris-Saclay, Gif-sur-Yvette 91198, France; ^e^Instituto de Bioingeniería, Universidad Miguel Hernández, Elche 03202, Spain; ^f^Institute of Genetics Reproduction and Development (iGReD), Université Clermont Auvergne, CNRS, Inserm, Clermont-Ferrand F-63000, France; ^g^Institut Jean-Pierre Bourgin, INRA, AgroParisTech, CNRS, Université Paris-Saclay, Versailles 78000, France; ^h^Molecular and Cell Biology of Plants, Goethe University Frankfurt, Frankfurt D-60438, Germany; ^i^State Key Laboratory of Genetic Engineering, Collaborative Innovation Center of Genetics and Development, Department of Biochemistry, Institute of Plant Biology, School of Life Sciences, Fudan University, Shanghai 200438, China; ^j^State Key Laboratory of Plant Physiology and Biochemistry, College of Life Sciences, Zhejiang University, Hangzhou 310058, China; ^k^Laboratory for Genome Engineering and Synthetic Biology, Division of Biological Sciences, 4700 King Abdullah University of Science and Technology, Thuwal 23955-6900, Saudi Arabia; ^l^Instituto de Agrobiotecnología del Litoral, CONICET, Universidad Nacional del Litoral, Santa Fe 3000, Argentina; ^m^Institut Universitaire de France, Orsay, Gif-sur-Yvette 91190, France

**Keywords:** chromatin, *Solanum lycopersicum*, genome topology

## Abstract

In recent years, the exploration of genome three-dimensional (3D) conformation has yielded profound insights into the regulation of gene expression and cellular functions in both animals and plants. While animals exhibit a characteristic genome topology defined by topologically associating domains (TADs), plants display similar features with a more diverse conformation across species. Employing advanced high-throughput sequencing and microscopy techniques, we investigated the landscape of 26 histone modifications and RNA polymerase II distribution in tomato (*Solanum lycopersicum*). Our study unveiled a rich and nuanced epigenetic landscape, shedding light on distinct chromatin states associated with heterochromatin formation and gene silencing. Moreover, we elucidated the intricate interplay between these chromatin states and the overall topology of the genome. Employing a genetic approach, we delved into the role of the histone modification H3K9ac in genome topology. Notably, our investigation revealed that the ectopic deposition of this chromatin mark triggered a reorganization of the 3D chromatin structure, defining different TAD-like borders. Our work emphasizes the critical role of H3K9ac in shaping the topology of the tomato genome, providing valuable insights into the epigenetic landscape of this agriculturally significant crop species.

## Significance

The investigation into the three-dimensional (3D) conformation of the genome has yielded crucial insights into the regulation of gene expression across both animal and plant kingdoms. Unlike animals, where genome topology is defined by topologically associating domains (TADs), plants present a comparable yet more diversified conformation across species. Addressing the unique challenge of defining TAD-like borders in plants, which lack the key architectural protein CTCF (CCCTC-binding factor), we delve into the intricate interplay between histone modifications and the 3D organization of chromatin. Our study unveils H3K9ac as a pivotal determinant, playing a major role in shaping the overall organization of chromatin in plants, providing a unique perspective on the distinctive chromatin dynamics in the absence of CTCF.

In recent decades, there has been considerable research focused on deciphering the intricate regulatory language employed by organisms to modulate molecular, biochemical, and structural genomic alterations without modifying their DNA sequence. Epigenetic modifications provide a dynamic and reversible layer of regulation that can rapidly respond to environmental cues. In eukaryotes, epigenetic modifications across genomes occur at multiple levels. Among these, histone modifications play a pivotal role in chromosome dynamics. Histone methylation dynamics is particularly relevant due to its association with repressive gene expression and chromatin remodeling (1). Indeed, although histone methylation does not alter the electrostatic interactions between histones and DNA, this mark can lead to a more compact chromatin structure that promotes transcriptional repression when deposited on particular residues, such as lysines 9 or 27 of histone H3. Recent studies have implicated histone methylation in regulating chromatin three-dimensional (3D) conformation, further highlighting the interplay between transcriptional activity and the spatial organization of genetic information (2–5).

The study of 3D organization of chromatin within the nucleus has provided valuable insights into the regulation of gene expression and cellular function in both animals and plants (6, 7). In mammals, genome topology is characterized by the formation of discrete chromatin domains called topologically associating domains (TADs), which are thought to facilitate gene regulation by bringing distal regulatory elements into physical proximity with their target genes (8). TADs are crucial in establishing and maintaining cell type–specific gene expression patterns and are associated with the spatial segregation of active and repressive chromatin compartments. Additionally, enhancer-promoter interactions within TADs contribute to the precise spatiotemporal control of gene expression during development and cellular differentiation (9–12).

Studies on plant genome topology have revealed both common and distinctive features compared to mammals. Both kingdoms exhibit the clustering of transcriptionally active genes into spatially proximal regions and the separation of active and repressive chromatin compartments (5, 13, 14). Additionally, the presence of chromatin loops and interactions between regulatory elements and gene promoters is a shared characteristic of genome topology in animals and plants, albeit with variations in their frequency and scale (15). However, unlike animals, plants lack CTCF (CCCTC-binding factor), a key architectural protein involved in shaping the 3D structure of chromatin and mediating long-range chromatin interactions. Accordingly, the *Arabidopsis* genome does not exhibit canonical TAD structures (16), and modeling approaches recently suggested that its overall organization is primarily driven by constitutive heterochromatin (CH) and nucleolar organizing regions (17). Further studies of chromatin topology in plants led to the consensus that the organization of the *Arabidopsis* genome is unusual and that most plant genomes fold into TAD-like structures that resemble TADs in chromosome conformation capture experiments, but are functionally different. Indeed, these TAD-like structures appear to be organized around local chromatin compartments defined by their epigenetic status (active, polycomb or repressive, (reviewed in ref. 18). In crops with large genomes such as wheat, tomato, or maize, these TAD-like structures bring together constitutive heterochromatin regions, whereas genes are mostly found in the borders (18, 19), suggesting that histone marks characteristic of constitutive heterochromatin could contribute to shaping the global 3D genome organization.

The dynamic modulation of histone methylation hinges on the interplay between histone methyltransferase enzymes, often referred to as writers, and histone demethylases, known as erasers (1). Histone lysine methyltransferases (HKMTs) feature a distinct, conserved domain known as the SET (Suppressor of variegation, Enhancer of zeste and Trithorax) domain, serving as the chief architect of histone methylation activity. Among HKMTs, KRYPTONITE (KYP, also known as SUVH4), SUVH5, and SUVH6 have been extensively characterized as histone H3K9 methyltransferases. KYP, SUVH5, and SUVH6 collaboratively contribute to the silencing of TEs through the regulation of H3K9me1 and H3K9me2 at specific genomic loci. Additionally, H3K9me1/2 plays a pivotal role in maintaining non-CG DNA methylation through a positive feedback loop involving the DNA methyltransferases CHROMOMETHYLASE 2 (CMT2), which methylates mostly CHH sites, and CMT3, which mostly function at CHG sites (20–22). Interestingly, recent research has suggested that both KYP and CMT3 play a vital role in the phenomenon of paramutation at the *sulfurea* (*sulf*) locus in tomato, which is associated with local remodeling of chromatin 3D organization (23), providing further evidence that constitutive heterochromatin marks are important determinants of chromatin folding.

Although the molecular mechanisms controlling the distribution of chromatin marks are increasingly well understood, as is the case for constitutive heterochromatin, a few studies have experimentally investigated how histone posttranslational modifications drive 3D genome organization in plants. Due to the scarcity of genetic resources in other plant models, most studies have been focused on *Arabidopsis*, which is atypical in terms of genome size and organization (24).

While extensive genomic and transcriptomic resources have been generated for tomato, our knowledge of the epigenetic landscape remains limited, thus limiting our understanding of the regulatory mechanisms governing its growth, development, and response to environmental stimuli. In this study, we present an integrative analysis of 26 histone modifications, Pol II distribution, and chromatin conformation for tomato. This chromatin atlas provides a comprehensive view of the distribution of epigenetic marks and their association with differential chromatin states, gene expression, and chromatin architecture. Using this resource, we have explored the functional relationship between histone modifications and chromatin 3D organization. Using the *kyp* mutant, which is known to display extensive changes in chromatin modifications (25), we have revealed a hitherto unrecognized role for H3K9ac in the determination of TAD-like boundaries in plants.

## Results

### Distinct Alternative Euchromatic and Heterochromatic Landscapes Characterize the Tomato Genome.

**Fig. 1.**
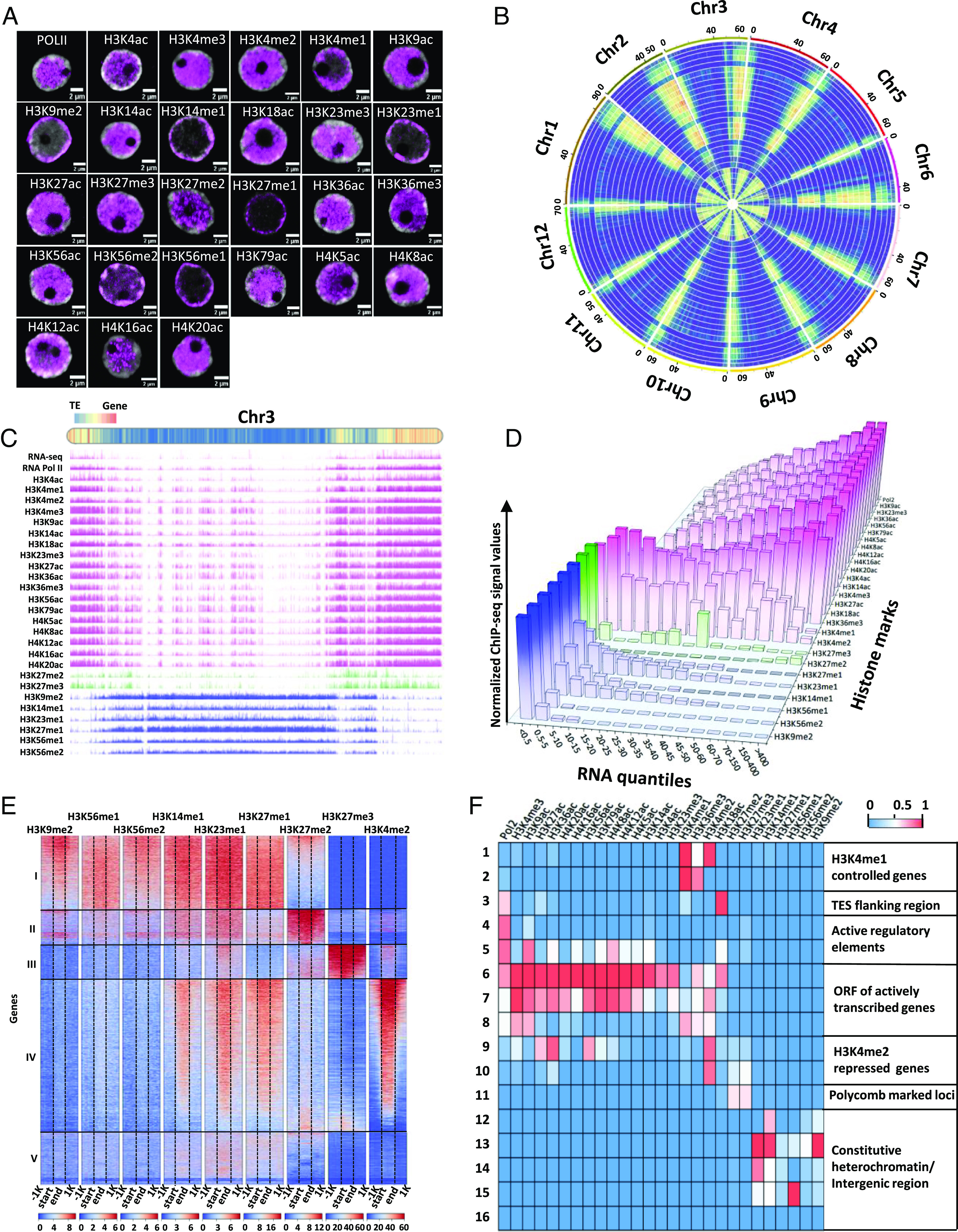
High-resolution identification of tomato chromatin states. (*A*) Immunofluorescence detection of 26 histone modifications and RNA Pol II (pink) and DAPI staining (gray) in isolated tomato nuclei. (*B*) Circos plot summarizing the chromosomal distribution of histone marks. The outermost circle depicts the ideograms of each chromosome. Bar plots present the density of corresponding signals. From outside to inner layer: RNA-seq, Assay for Transposase-Accessible Chromatin using sequencing (ATAC-seq), RNA Pol II, H3K9ac, H3K23me3, H3K36ac, H3K56ac, H3K79ac, H4K5ac, H4K8ac, H4K12ac, H4K16ac, H4K20ac, H3K4ac, H3K14ac, H3K4me3, H3K27ac, H3K18ac, H3K36me3, H3K4me1, H3K4me2, H3K27me3, H3K27me2, H3K27me1, H3K23me1, H3K14me1, H3K56me1, H3K56me2, and H3K9me2. (*C*) JBrowse view illustrating chromosome 3. Histone marks linked to euchromatin (pink), FH (green), and CH (blue). (*D*) Correlation between average enrichment profile of histone modifications and gene expression variations. Gene expression is classified from low (1st quantile) to high expression (16th quantile). Median-normalized ChIP-seq densities of equal bins along genes associated with each quantile were plotted for each histone mark. (*E*) Enrichment profiles of repressive marks and H3K4me2 euchromatin mark over not expressed genes. There were five clusters showing a correlation between average enrichment profiles of histone modifications and gene expression variations. The number of genes for each cluster is I: 3,126, II: 1,326, III: 1,236, IV: 7,133, and V: 2,317. (*F*) Genome-wide chromatin state matrix determined with a multivariate hidden Markov model. The matrix shows the emission probabilities for histone modifications across 16 chromatin states.

In order to set the basis for an extensive understanding of genome organization and its interplay with gene activity in tomato, we undertook complementary cell biology and biochemical approaches. First, we investigated tomato nuclei organization using transmission electron microscopy. This analysis revealed that heterochromatin is strongly associated with the nuclear envelope (*SI Appendix*, Fig. S1 *A* and *B*). Second, we performed immuno-localization of a subset of 26 histone marks and RNA polymerase II (RNA Pol II, Fig. 1*A*). The localization of RNA Pol II across the nucleus coincided with 17 euchromatic histone marks, as well as with the facultative heterochromatin (FH)-related marks H3K27me2 and H3K27me3. As expected, the two constitutive heterochromatin related marks H3K9me2 and H3K27me1 exhibited a peripheric distribution, in proximity to the nuclear envelope. Interestingly, our results also revealed the localization of the histone mark H3K23me3 in the center of the nucleus and four other marks: H3K14me1, H3K23me1, H3K56me1, and H3K56me2, localized in the constitutive heterochromatic area. This suggested that these four histone marks are characteristic heterochromatic features (Fig. 1*A* and *SI Appendix*, Fig. S1*C*).

To go further, we mapped by Chromatin immunoprecipitation followed by sequencing (ChIP-seq) the distribution of these histone marks and RNA Pol II across the tomato genome. This analysis revealed a genome-wide view of the distribution of histone marks in heterochromatic and euchromatic genome compartments (see Fig. 1*B* for a global analysis and Fig. 1*C* for a detailed plot of chromosome 3). As expected, we observed a correlation between the immuno-localization analysis and the positioning of histone marks. The deposition of RNA Pol II revealed that gene expression occurs mostly at chromosome arms, in association with the distribution of permissive and facultative heterochromatin histone marks. Notably, we confirmed that the histone mark H3K23me3, and the histone marks H3K14me1, H3K23me1, H3K56me1, and H3K56me2, serve as euchromatin and constitutive heterochromatin signatures, respectively (Fig. 1*C*).

To identify the relationships between histone mark signatures and gene transcriptional activity, we conducted RNA sequencing (RNA-seq) analysis. Protein coding genes were classified into 16 groups according to their expression level (*SI Appendix*, Figs. S2 and S3). This classification of gene expression revealed that repressed and active genes were distinct in terms of epigenetic profiles and that a large repertoire of histone modifications is associated with each state (Fig. 1*D* and *SI Appendix*, Fig. S3). We found that six constitutive heterochromatinmarks were almost exclusively on genes belonging to the lowest quantile of expression, and this was also the case for the facultative heterochromatin marks H3K27me2 and H3K27me3. By contrast, different euchromatin-related marks were found to be associated with highly and lowly expressed genes (Fig. 1*D*). Interestingly, genes exhibiting low expression levels were enriched in H3K4me1 and H3K4me2, and their enrichment decreased along with higher transcript levels (Fig. 1*D*). Moreover, H3K4me1 enrichment was notably reduced in nonexpressed genes (*SI Appendix*, Fig. S3*R*). On the other hand, a high accumulation of gene transcripts is highly correlated with the enrichment of the other 18 marks which are signatures of euchromatin. With this in mind, we conducted a comprehensive correlation of the main constitutive heterochromatin signatures, including the constitutive histone marks (H3K14me1, H3K23me1, H3K56me1, and H3K56me2), as well as facultative heterochromatin and euchromatin signatures over genes belonging to the lowest quantile (i.e., not expressed). We found distinct epigenetic profiles that are associated with heterochromatin formation and gene silencing (Fig. 1*E*). Five different chromatin signatures were identified from our correlation analysis (*SI Appendix*, Fig. S4). The first, representing 21% of genes, was mainly enriched with heterochromatin-related marks, with a small proportion of genes showing association with the facultative heterochromatin mark H3K27me2. The second, accounting for 9% of genes, was highly enriched for H3K27me2 and to a lesser extent for constitutive heterochromatin marks. Notably, the third signature (8%) was associated with H3K27me3, indicating that they belong to polycomb repressive complex II-controlled genes and represent only a small subset of transcriptionally repressed genes. Remarkably, the fourth signature corresponded to a significant proportion of repressed genes (47%) that are associated with the histone mark H3K4me2. Finally, the fifth group represents 15% of repressed and has a less clear signature, appearing to be enriched for the constitutive heterochromatin-related histone marks H3K14me1, H3K23me1, and H3K27me1. These chromatin signatures revealed by ChIP-seq were in agreement with the immunolocalization using specific antibodies (Fig. 1*A*), revealing that the central and peripheral distribution of chromatin marks defines two distinct transcriptionally inactive compartments.

The distribution of the 26 different histone marks and RNA Pol II, allowed us to define 16 different chromatin states in the tomato genome (Fig. 1*F* and *SI Appendix*, Fig. S5). Among those, one corresponded to Polycomb-repressed genes, and two to H3K4me2 repressed genes, further highlighting that H3K27me3 deposition does not appear to be the only histone mark associated with gene transcriptional repression. In addition, each chromatin state is linked to positional features such as distal enhancers, proximal enhancers intergenic regions among others (*SI Appendix*, Fig. S6). This classification allows the definition of chromatin features associated with the regulation of gene expression and reveals the distinctive euchromatic and heterochromatic features that characterize the tomato genome.

### Compartmental Domains Correlate with Histone Modifications.

**Fig. 2.**
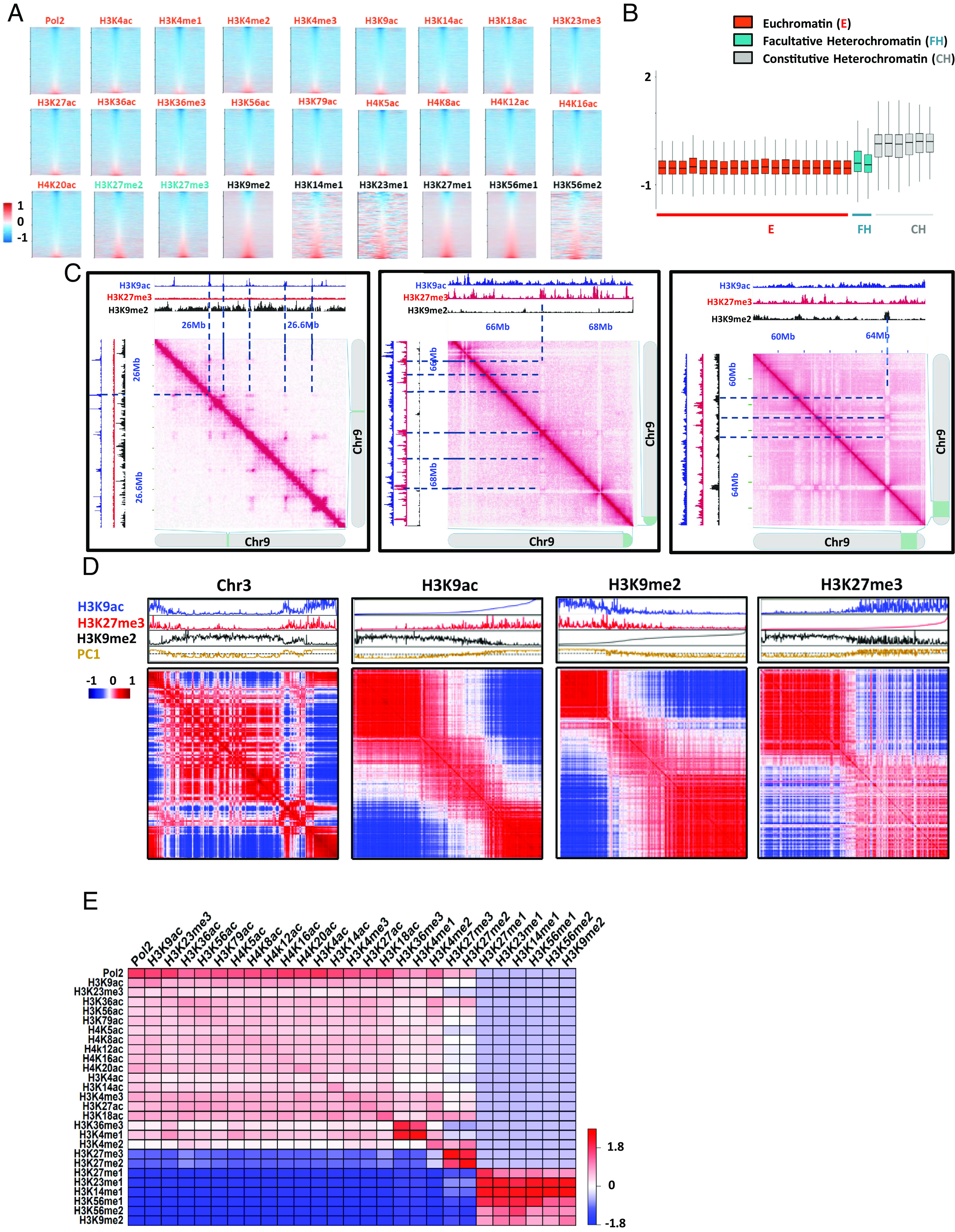
Genome-wide topographical relationship between histone modification and 3D genome organization. (*A*) Relationship between the insulation index and histone marks. Heat map displaying the insulation index over histone modifications peaks. (*B*) Box plot representing the median of the insulation index associated with histone modifications peaks. Euchromatin (E) represents euchromatin histone modification markers: Pol2, H3K9ac, H3K18ac, H3K27ac, H3K23me3, H3K36ac, H3K56ac, H3K79ac, H4K5ac, H4K8ac, H4K12ac, H4K16ac, H4K20ac, H3K4ac, H3K14ac, H3K4me3, H3K36me3, H3K4me1, and H3K4me2. FH represents facultative heterochromatin histone modification markers: H3K27me3 and H3K27me2. CH represents constitutive heterochromatin histone modification markers: H3K27me1, H3K23me1, H3K14me1, H3K56me1, H3K56me2, and H3K9me2. (*C*) 2D heat maps of contact matrices with interaction frequencies in different regions of chromosome 9. (*D*) Pearson correlations for distance-normalized Hi-C interaction frequency map of chromosome 3. Shown above each Hi-C map from *Top* to *Bottom* are the following: H3K9ac signal (blue), H3K27me3 signal (red), and H3K9me2 signal (black). Principal component 1 distribution (PC1) in brown. From *Left* to *Right*: i) interactions among sequences from chromosome 3 arranged in natural order; ii) interactions among sequences from chromosome 3 sorted by H3K9ac levels, from lowest to highest; iii) interactions among sequences from chromosome 3 sorted by H3K9me2 levels, from lowest to highest; and iv) interactions among sequences from chromosome 3 sorted by H3K27me3 levels, from lowest to highest. (*E*) Heatmap presenting the logarithm of odd ratios of all feature combinations of interacting loci. Positive log_2_(odds ratios) indicates enrichment and negative indicates depletion.

To investigate the link between histone marks, chromatin states, and genome topology, we performed Hi-C assays and calculated the insulation index of regions enriched for RNA Pol II or each of the assessed histone marks (Fig. 2*A*) and chromatin states (*SI Appendix*, Fig. S7). The median of the frequency of interaction with the neighboring regions was lower for regions enriched with euchromatin-related marks than for constitutive heterochromatin, whereas facultative heterochromatin associated regions exhibited an intermediate contact behavior (Fig. 2*B*). Hence, we further analyzed a distinctive euchromatin mark (H3K9ac), as well as facultative and constitutive heterochromatin histone marks (H3K27me3 and H3K9me2, respectively). A Visual inspection of the interaction matrix revealed the widespread presence of interaction hotspots between genomic bins associated with the same histone modifications (Fig. 2*C*). Therefore, to evaluate the potential of a genomic region to establish a distinct compartmental domain characterized by particular chromatin marks, we produced a Pearson correlation map and organized it based on various chromatin features. In this process, we restructured the rows and columns within the correlation matrix. Instead of arranging them based on their linear sequence position, we sorted the bins in ascending order of the selected feature’s signal strength (Fig. 2*D* and *SI Appendix*, Fig. S8). Sorting by H3K9ac signal value, we observed a strong compartmentalization, as seen with chromosome 3 (Fig. 2*D*), and on all chromosomes (*SI Appendix*, Fig. S8). The matrix displayed a clear segregation which is characterized by an anticorrelated enrichment of H3K9ac and H3K9me2 histone marks. Reciprocally, sorting by the H3K9me2 signal value also resulted in a clear compartmentalization due to the strong anticorrelated enrichment between these two histone marks. By contrast, when sorting the chromosome by H3K27me3 signal value, we also observed compartmental segregation, but less pronounced as compared to H3K9ac sorting. Notably, this patterning occurred in all the tomato chromosomes (*SI Appendix*, Fig. S8), suggesting that H3K9ac and H3K9me2 are important driving elements for genome compartmentalization. Finally, to reveal the most frequent interactions among the 26 histone marks and RNA Pol II, we calculated the odds ratios (ORs) of their interactions (*Materials and Methods*). Analysis of all feature combinations of interacting loci across the genome revealed that chromatin interactions occur more frequently between regions with concordant epigenetic status (Fig. 2*E* and *SI Appendix*, Fig. S9). Altogether, our results hint at an intricate relationship between histone modifications and genome topology organization, and subsequently gene activity.

### H3K9ac Plays a Major Role in Genome Topology Organization.

**Fig. 3.**
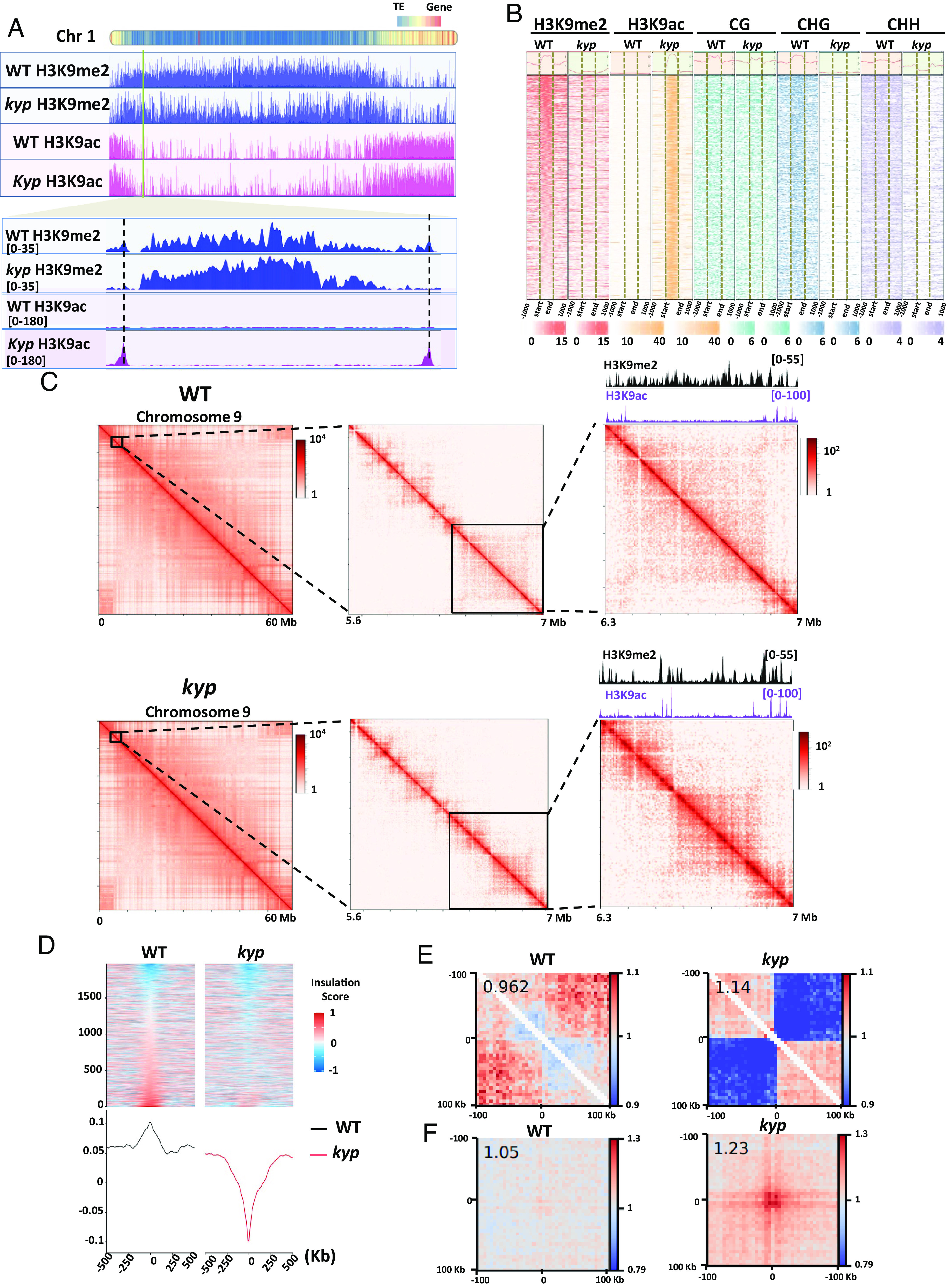
H3K9ac plays a major role in defining TAD-like border. (*A*) JBrowse view illustrating chromosome 1. H3K9me2 (blue) and H3K9ac (pink) enrichment in both WT and *kyp*. Zoomed-in chromosome 1 region illustrating H3K9me2 loss and H3K9ac gain in *kyp*. (*B*) Heatmaps showing the distribution of H3K9me2, H3K9ac, and DNA methylation signals on genomic regions that gain H3K9ac in *kyp*. Sample size n = 3. (*C*) Hi-C contact matrices reveal a change in *kyp* upon switch from H3K9me2 to H3K9ac in chromosome 9. (*D*) Heatmap showing the insulation index over genomic loci that display a switch from H3K9me2 to H3K9ac in *kyp*. (*E*) Local pile-ups of genomic loci that display a switch from H3K9me2 to H3K9ac in *kyp*, indicating depletion of local associations within TAD-like structures. The enrichment score is displayed on *Top* *Left*. (*F*) Pile-ups of interactions between genomic loci that display a switch from H3K9me2 to H3K9ac in *kyp*, indicate the emergence of distant contact points. The enrichment score in the central pixel is displayed on *Top* *Left*. The distance from the peak region is 100 Kb.

The thorough characterization of tomato chromatin states and their correlation with genome topology revealed a distinct and exclusive pattern of H3K9ac and H3K9me2 distribution that are closely tied to the spatial organization of euchromatin and heterochromatin compartments. To test the role of these two chromatin marks on genome topology we use the mutant *kyp*, which is known to affect H3K9me2 deposition (25). We mapped H3K9me2 and H3K9ac by ChIP-seq in WT and *kyp* plants (Fig. 3*A* and *SI Appendix*, Fig. S10). This analysis revealed that the levels of H3K9me2 in *kyp* plants diminished in discrete pericentromeric regions. Notably, the regions where H3K9me2 was lost in *kyp* showed a sharp deposition of H3K9ac. To investigate why H3K9ac deposition occurs in specific regions, we analyzed the associated motifs and found a significant enrichment for the TATA binding protein (TBP) motif bound by the TBP. This protein is known to interact with the transcription factor IID and SAGA two protein complexes that display a histone acetyl-transferase activity (26) (*SI Appendix*, Fig. S11). Further analysis revealed that the global reduction of H3K9me2 in *kyp* correlated with a strong deposition of H3K9ac (Fig. 3 *A* and *B* and *SI Appendix*, Fig. S12).

**Fig. 4.**
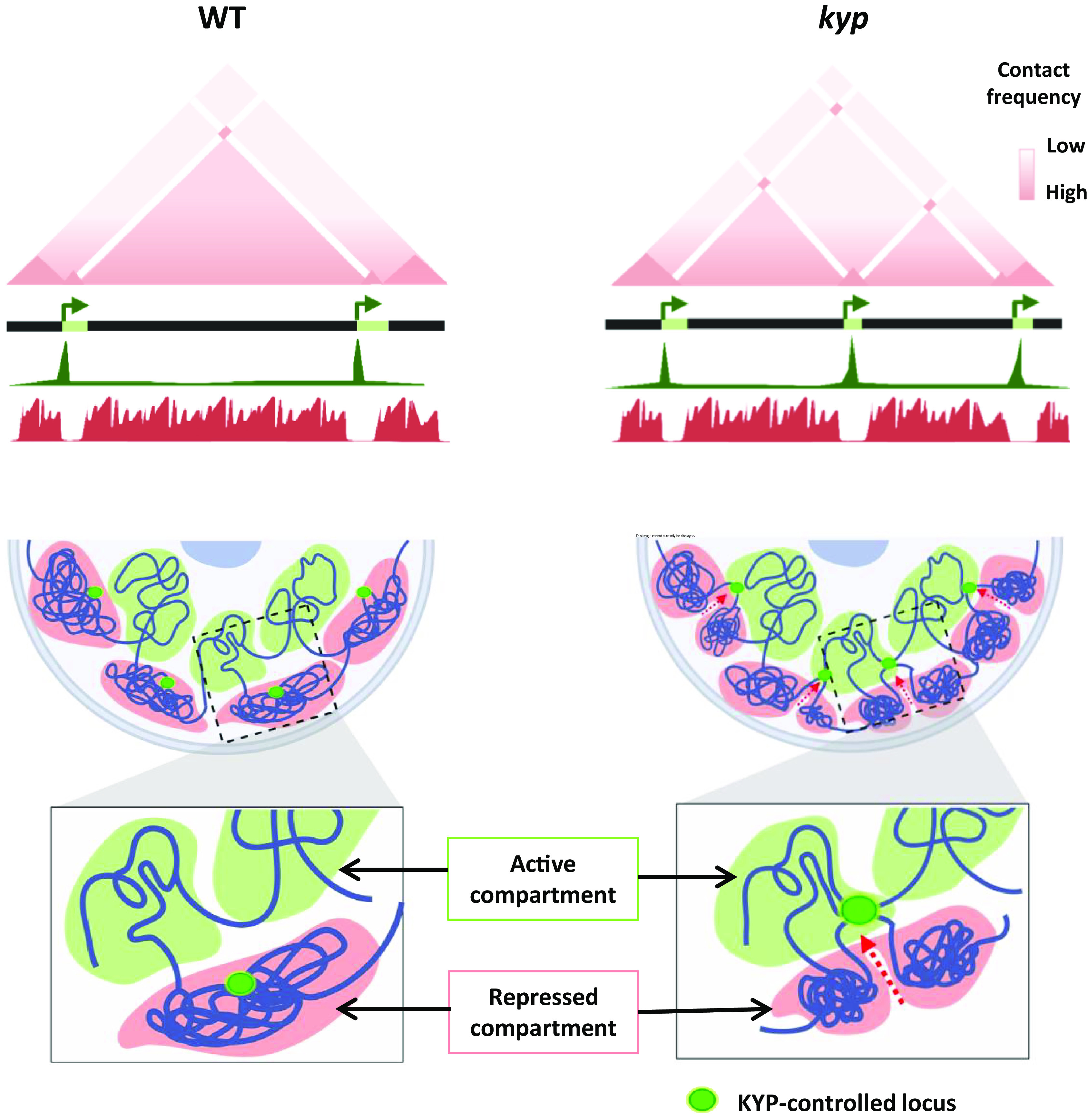
KYP shapes TAD-like boundaries through the control of H3K9ac. Regions on repressed compartments can act as anchors for TAD-like structure formation after the ectopic deposition of H3K9ac in *kyp*.

To determine the role of the ectopic H3K9ac deposition on genome topology, we performed Hi-C on WT and *kyp* plants. In the WT, TAD-like structures rich in H3K9me2 exhibit borders with high levels of H3K9ac (Fig. 3*C* and *SI Appendix*, Figs. S13–S16). Notably, the emergence of H3K9ac deposition within the same TAD-like structure *kyp* resulted in a reorganization of chromatin 3D conformation, determining different TAD-like borders and splitting the chromatin into two sub-self-interacting domains. The determination of the insulation index over genomic loci that display a switch from H3K9me2 to H3K9ac in *kyp* showed that this chromatin 3D reorganization is a global feature across the tomato genome (Fig. 3*D* and *SI Appendix*, Fig. S17). To understand the relationship between the level of H3K9ac and the insulation index, we divided the sites that gain H3K9ac in *kyp* into three categories (low, medium, and high levels) and plotted the insulation associated with each category. Interestingly, we observed a correlation between the H3K9ac level and the insulation (*SI Appendix*, Fig. S18). In addition, to determine to what extent this gain of H3K9ac and insulation correlate with chromatin accessibility, and gene expression, we performed ATAC-seq and RNA-seq. We found a positive correlation between these four features, suggesting that the local chromatin structure may play a role in 3D genome organization (*SI Appendix*, Fig. S18). Remarkably, a pile-up of chromatin interactions between genomic loci that display a switch from H3K9me2 to H3K9ac revealed that local associations (within TAD-like structures) are depleted (Fig. 3*E* and *SI Appendix*, Fig. S19), whereas distal contact points emerge (Fig. 3*F* and *SI Appendix*, Fig. S20), supporting the view that H3K9ac spots act as anchor points for newly formed TAD-like structures. Collectively, our results uncover the role of H3K9ac as a major determinant of chromatin organization and KYP as a modulator of chromatin topology in tomato (Fig. 4).

## Discussion

In this study, we conducted a comprehensive analysis of the epigenetic landscape and genome organization in tomato, aiming to shed knowledge on the role that histone modifications play in chromatin architecture in plants. Our work illuminates the intricate interplay between histone modifications, chromatin states, and 3D organization. The establishment of epigenome atlases spanning a growing number of species enhances our comprehension of gene expression regulation and provides important resources to investigate its orchestration in cell fate, development, and natural variation, as well as its relevance in the context of disease and stress responses (27–31). By mapping the distribution of epigenetic signatures across the tomato genome, we were able to define distinct euchromatic and heterochromatic states. Previous epigenomic studies in *Arabidopsis* (31) and rice (32) have shown the inverse correlation along the nuclear genome of euchromatin marks such as H3K9ac and constitutive heterochromatin marks. Our analysis has revealed the presence in plants of different constitutive heterochromatic marks, H3K14me1 or H3K56me1 and H3K56me2, which have been previously described in animals. These findings suggest that the repertoire of chromatin marks in plants is much broader than previously thought. In addition, we observed the existence of alternative epigenetic profiles even within euchromatic regions, which are associated with varying levels of gene expression and distinct parts of the genes, as previously reported in *Arabidopsis* (31). This observation implies that not all euchromatic domains share functional equivalence, and specific combinations of histone marks exert a role in dictating the transcriptional activity of genes. Notably in tomato, the simultaneous presence of H3K4me1 and intermediate acetylation levels appears to be a hallmark of moderate gene expression, whereas the most highly expressed genes display the highest histone acetylation levels. Furthermore, we found that only a small subset of genes is repressed by the H3K27me3 mark, whereas others seem to be repressed by the H3K4me2 mark, suggesting that histone modifications associated with transcriptional repression is more diverse in tomato than previously anticipated.

Beyond the identification of distinct chromatin states, our findings support that the 3D configuration of the tomato genome is intricately linked to histone modifications, which trigger the formation of chromatin interactions associated with the coregulation of spatially grouped loci. The characterization of 3D genome organization in plants was pioneered in the model species *Arabidopsis thaliana*. The first studies unveiled the absence of TAD structures and the existence of “A/B” type compartmentalization at a local scale, manifesting within domains spanning a few tens of kilobases (13, 14) The orchestration of this compartmentalization is significantly steered by constitutive heterochromatin, primarily composed of transposable elements that bear distinct histone modifications such as H3K9me2 and H3K27me1. However, the contribution of facultative heterochromatin to higher-order nuclear topology in plants long remained enigmatic. Recently, immuno-localization experiments in *Arabidopsis* have shown that H3K9ac-rich and H3K27me3-rich regions are spatially segregated, demonstrating that actively transcribed and repressed genes occupy distinct regions within the nucleus (33). In addition, changes in the distribution of these marks alter genome folding and chromatin interactions: It was observed that certain genomic regions in the *Arabidopsis* *clf* mutant, upon losing H3K27me3, exhibit a tendency to establish interactions with transcribed regions distinguished by H3K9ac marks, providing further evidence for the role of H3K9ac and H3K27me3 distribution on 3D genome organization (33). Likewise, an analogous approach was employed in maize, revealing that H3K27me3 mediates the formation of chromatin loops established between putative regulatory elements and their target genes, a process closely associated with gene repression (34). Here, we found that in tomato, a large set of repressed genes is not associated with H3K27me3 and that this mark is not as well correlated with genome compartmentalization as H3K9ac or H3K9me2, suggesting that gene expression and/or constitutive heterochromatin are the main drivers of global genome folding, although H3K27me3 may also contribute to global genome folding.

As mentioned before, local active and repressive compartments seem to play a pivotal role in plant 3D genome organization (18). In this study, we took advantage of a tomato mutant (*kyp*) that shows defects in the deposition of H3K9me2 (25). We found that in this mutant the lack of H3K9me2 was associated with the discrete deposition of H3K9ac and with the emergence of different TAD-like structures that restructure chromatin 3D conformation. This finding underscores the pivotal role of H3K9ac and H3K9me2 in shaping chromatin organization and suggests that KYP plays a central role in governing H3K9ac distribution by preventing its deposition. In line with this finding, recent evidence obtained in animal models indicated that histone hyperacetylation also exerts an influence on nuclear organization by inducing the formation of long-range chromatin interactions and nuclear subcompartments (35). The precise molecular mechanisms involved in the recruitment of acetylated loci remain unknown, but recent research in human cell lines suggested that the dynamics of transcriptional activity and RNA Pol II orchestrate distal interactions among protein-coding loci and regulatory elements situated in noncoding genomic regions (36). In tomato, an in-depth exploration of the heat-induced changes in nuclear architecture, histone modifications, chromatin accessibility, and transcriptional activity has also revealed that the tomato genome is organized around specific sites of transcriptionally active chromatin regions (37). These findings uncovered the distinct roles played by various levels of chromatin spatial organization, underscoring the pivotal regulatory significance of nuclear organization as a holistic entity. In the realm of tomato chromatin, a pronounced compartmentalization is evident, with a remarkable organization centered around specific sites of transcription within active chromatin regions that bear a striking resemblance to transcription factories, as previously described for wheat (19). While the precise mechanisms governing the organization of transcription factories remain elusive, they are believed to assume a critical role in clustering coregulated genes (38), and appear to be a common feature observed in most plant genomes. Collectively, these findings support the view that a complex interplay between the epigenome and transcription shapes the 3D organization of genetic information in plants and animals.

## Materials and Methods

### Plant Material, Growth, and Genotyping.

*Solanum lycopersicum* cv M82 was used for this study. The *kyp* mutant in M82 was donated by David C. Baulcombe (25). All seeds were sown in a commercial mix substrate under controlled conditions in growth chambers at 24 °C with a 16/8 h light/dark period. Genotyping of *kyp* plants was performed according to ref. 23. The fourth true leaf was used for all experiments in this study.

### Transmission Electron Microscopy.

Leaves were cut into thin pieces in a petri dish and then fixed for 10 min using vacuum infiltration with a fixation solution containing 3% glutaraldehyde and 1% PFA in 0.1 M cacodylate and 250 µM CaCl_2_. Following several washes with 0.1 M sodium cacodylate buffer, the plant tissue was postfixed with 1% OsO_4_ and 1.5% potassium ferricyanide in 0.1 M sodium cacodylate for 1 h at room temperature. Subsequently, the plant tissues were washed with H_2_O. Afterward, the tissues were dehydrated using a series of ethanol baths (10%, 20%, 30%, 50%, 70%, 90%, 100%), each for 30 min. The plant tissues were subsequently embedded in a mixture of resin and propylene oxide, then in pure epoxy resin, for a total duration of 72 h. Samples were polymerized for 24 h at 60 °C. Then, 80-nm ultrathin sections were prepared with an ultramicrotome (UC6, Leica Microsystems), counterstained with 2% uranyl acetate and lead citrate. Finally, the prepared plant material was observed with a transmission electron microscope operating at 80 kV (JEOL 1400).

### Immunolocalization Assay.

Immunofluorescence labeling assays were conducted according to ref. 39. Four-week-old leaves were fixed, and nuclei were isolated, placed on a poly-lysine slide, and incubated overnight at 4 °C with primary antibodies, 400× diluted, listed in *SI Appendix*, Table S1. Slides were washed and incubated during 1 h at room temperature in dark with Goat anti-Rabbit Alexa Fluor Plus 488 (A11034 Invitrogen) and Goat anti-Mouse Alexa Fluor Plus 555 (A32727 Invitrogen) or with Goat anti-Rabbit Alexa Fluor Plus 555 (A32732 Invitrogen) and Goat anti-Mouse Alexa Fluor Plus 488 (A32723 Invitrogen) secondary antibodies (400× diluted). DNA was counterstained with DAPI in SlowFade Diamond Antifade mounting media. Slides were directly imaged on a confocal microscope (Zeiss Microsystems).

### ChIP-seq Assay.

ChIP-seq assays were performed on the fourth leaves of 4-wk-old tomato plants using the antibodies listed in *SI Appendix*, Table S1, following the procedure described in ref. 40. ChIP-seq libraries were prepared from 10 ng of DNA using the NEBNext Ultra II DNA Library Prep Kit for Illumina (NEB) according to the manufacturer’s instructions. Two independent biological replicates were generated for each condition. The quality of DNA libraries was assessed with the Agilent 2100 Bioanalyzer. Single-end 75 bp reads were produced by the Illumina NextSeq 500 platform.

### ChIP-seq Data Analysis.

Adapter removal from raw sequencing data was performed using Trimmomatic v0.38 (41). Parameters for read quality filtering were set as follows: minimum length of 30 bp; mean Phred quality score greater than 30; and leading and trailing base removal with base quality below 5. Cleaned reads were aligned to tomato M82 genome v1 (42) using Bowtie2 v2.3.5 using mode “--very-sensitive” (43). Unmapped reads, PCR duplicates, and low mapping quality alignments (MAPQ < 30) were discarded for further analysis. MACS2 v 2.2.7.1 was used for peak detection (44). For broad peaks, parameters were set as follows: --broad --nomodel --extsize 147 -g 829069930 -p 0.05 --extsize 150 --bw 500. For sharp peaks, parameters were set as “-g 829069930 -p 0.05 --extsize 150 --bw 500.” Visualization of histone modification profiles was conducted with JBrowse (45) and r package EnrichedHeatmap (46).

To define genome-wide chromatin states for all histone modifications, ChromHMM v 1.22 was used with default parameters (47, 48). Peaks for each histone mark were selected individually using a *P*-value cutoff of 0.05, pileup > 36, and fold change > 1.5. The peaks were binarized with ChroHMM’s BinarizeBed method setting parameters “-b 150 -peaks -center.” Chromatin state models were learned jointly on all histone markers ranging from 15 to 20 states. Finally, a model of 16 chromatin states was selected for further analysis.

### RNA-seq Library Preparation and Sequencing.

Total RNA was extracted using the Nucleospin RNA kit (Macherey-Nagel), according to the manufacturer’s instructions. Complementary DNA libraries were produced using 2 μg of total RNA with the NEBNext Ultra II Directional RNA library Preparation Kit (NEB) according to the manufacturer’s instructions. Quantification and quality control of libraries were performed using an Agilent 2100 Bioanalyzer. Libraries were sequenced in pair-end on the Illumina NovaSeq 6000 platform. The average targeted length obtained per read was 150 bp. Three biological replicates were used for each genotype.

### RNA-seq Data Analysis.

Adapters removal from raw sequencing data was performed using Trimmomatic v0.38 (41). Cleaned reads were mapped to the tomato M82 genome v1 using Bowtie2 algorithm v2.3.5 (43) with “very sensitive” mode. After removing unmapped reads, PCR duplicates, and low mapping quality alignments (MAPQ < 30), aligned bam files were used for transcript expression estimation. Read density was assessed using the bamCoverage function of deeptools v3.5.0 (49) software with default parameters. Sixteen quantiles of gene expression values for the whole genome were calculated on the basis of the coverage bigwig generated in the previous step, considering only wild-type tomato RNAseq samples, using deeptools v3.5.0 (49) software with the multiBigwigSummary function.

### Hi-C Assay.

Two biological replicates for both the wild type and *kyp* mutant were processed according to ref. 50 using the *Dpn*II enzyme (New England Biolabs) and the *Dde*I enzyme (New England Biolabs). DNA library preparation was performed with the NEBNext Ultra II DNA library preparation kit (NEB). For the PCR amplification step, 10 cycles were used for libraries. Libraries were quantified and quality-checked using the Agilent 2100 Bioanalyzer, then sequenced on Illumina NextSeq 500 and NovaSeq 6000 instruments.

### Hi-C Data Analysis.

#### Construction of contact maps.

Raw Illumina sequencing fastq files were processed with Trimmomatic v0.38 (41). Cleaned reads were mapped independently to M82 v1 reference genome (42) using HiC-Pro v3.0.0 (51). Bowtie2 parameter settings were default, expect “BOWTIE2_GLOBAL_OPTIONS = --very-sensitive -L 30 --score-min L, -0.6, -0.6 --end-to-end –reorder” and “BOWTIE2_LOCAL_OPTIONS = --very-sensitive -L 20 --score-min L, -0.6, -0.6 --end-to-end --reorder” Forward and reverse mapped reads were paired and assigned to *Dpn*II and *Dde*I restriction fragments. Invalid ligation reads, such as dangling ends, were filtered. The mapping statistics for hic libraries are listed in *SI Appendix*, Table S2. Valid pairs were used to generate normalized matrices with the JUICER v1.9.9 (52) KR normalization method. Visualization of interactions was implemented with Juicebox v 2.16.0 (52).

#### Calculation of the insulation score and compartmentalization visualization.

TAD calling and TAD boundary insulation scores were performed with R package GENOVA (v 0.95) (53). Calculation and visualization of Hi-C compartmentalization were used with the same strategy as Nichols and Corces (54). Visualization of the Hi-C heatmap was performed with HiCExplorer (v 3.6) (9). Aggregated pile-up analysis was conducted with the coolpup package (v 1.1.0) (55).

#### Quantification and statistical analysis.

Valid pairs were further used to identify interactions with HOMER (v 4.11) (56) for 1 kb resolution. Annotation of each histone modification with interaction was performed with bedtools (v 2.28.0) intersect (57) at a threshold of *P* value 0.01 and interaction zscore > 200. R package GeneOverlap (v3.17) (58) was used to calculate the ORs, and z score transformation was implemented with R (v 4.3) function sapply.

## DNA Methylation Data.

DNA methylation data of M82 and *kyp* mutants were downloaded from ref. 25. Adapters were removed with Trimmomatic (v0.38) (41). Clean reads were mapped onto tomato M82 genome (42) using Bismark (v0.24.0) (59) with option -N 1 and nucleotide coverage. Deduplicate_bismark was used to deduplicate alignments, and bismark_methylation_extractor with option -ignore_r2 2 was used to extract methylation status. Bigwig files were generated with bismark2bedGraph and bedgraph2bigwig.

## Supplementary Material

Appendix 01 (PDF)

## Data Availability

All sequencing data generated in this study have been deposited in the NCBI Gene Expression Omnibus (GEO) database with accession GSE245529 (60). All other study data are included in the article and/or *SI Appendix*. Previously published data were used for this work (DOI: https://doi.org/10.1038/s41467-020-14995-6 NCBI Sequence Read Archive accession number PRJNA516166) (61).
