# Supplementary material for: An atlas of the tomato epigenome reveals that KRYPTONITE shapes TAD-like boundaries through the control of H3K9ac distribution
Source: Proc Natl Acad Sci U S A. 2024 Jul 5;121(28):e2400737121. doi: 10.1073/pnas.2400737121 (PMC11252963; doi:10.1073/pnas.2400737121)
Supplement: Supplementary file 1 — Appendix 01 (PDF) [file pnas.2400737121.sapp.pdf]

PNAS

Supporting Information for

An atlas of the tomato epigenome reveals that KRYPTONITE shapes TAD-like boundaries through the control of H3K9ac distribution

Jing An[a,1], Rim Brik Chaouche[a], Leonardo I. Pereyra-Bistraín[b,a], Hugo Zalzalé[b,a], Qingyi Wang[a], Ying Huang[a], Xiaoning He[a], Chloé Dias Lopes[a], Javier Antunez-Sanchez[c], Catherine Bergounioux[a], Claire Boulogne[d], Cynthia Dupas[d], Cynthia Gillet[d], José Manuel Pérez-Pérez[e], Olivier Mathieu[f], Nicolas Bouché[g], Sotirios Fragkostefanakis[h], Yijing Zhang[i], Shaojian Zheng[j], Martin Crespi[a], Magdy M. Mahfouz[k], Federico Ariel[l], Jose Gutierrez-Marcos[c], Cécile Raynaud[a], David Latrasse[a] and Moussa Benhamed[a,b,m*]

a Université Paris-Saclay, CNRS, INRAE, Univ Evry, Institute of Plant Sciences Paris-Saclay (IPS2), 91405 Orsay, France.
b Université de Paris Cité, Institute of Plant Sciences Paris-Saclay (IPS2), F-91190 Gif-sur-Yvette, France.
c School of Life Science, University of Warwick, Coventry CV4 7AL, UK. d Imagerie-Gif, Electron Microscopy Facility, Institute for Integrative Biology of the Cell (I2BC), CEA, CNRS, Univ. Paris-Sud, Université Paris-Saclay, Gif-sur-Yvette, France.
e Instituto de Bioingeniería, Universidad Miguel Hernández, 03202 Elche, Spain.
f Institute of Genetics Reproduction and Development (iGReD), Université Clermont Auvergne, CNRS, Inserm, F-63000 Clermont-Ferrand, France.
g Institut Jean-Pierre Bourgin, INRA, AgroParisTech, CNRS, Université Paris-Saclay, 78000 Versailles, France
h Molecular and Cell Biology of Plants, Goethe University Frankfurt, Frankfurt D-60438, Germany.
i State Key Laboratory of Genetic Engineering, Collaborative Innovation Center of Genetics and Development, Department of Biochemistry, Institute of Plant Biology, School of Life Sciences, Fudan University, 200438 Shanghai, China.
j State Key Laboratory of Plant Physiology and Biochemistry, College of Life Sciences, Zheijang University, 310058 Hangzhou, China.
k Laboratory for Genome Engineering and Synthetic Biology, Division of Biological Sciences, 4700 King Abdullah University of Science and Technology (KAUST), Thuwal 23955-6900, Saudi Arabia.
l Instituto de Agrobiotecnología del Litoral, CONICET, Universidad Nacional del Litoral, Santa Fe, Argentina.
m Institut Universitaire de France (IUF), Orsay, France.

* Moussa Benhamed
Email: moussa.benhamed@u-psud.fr

This file includes:

Supporting text
Figures S1 to S20
Tables S1 to S2

a

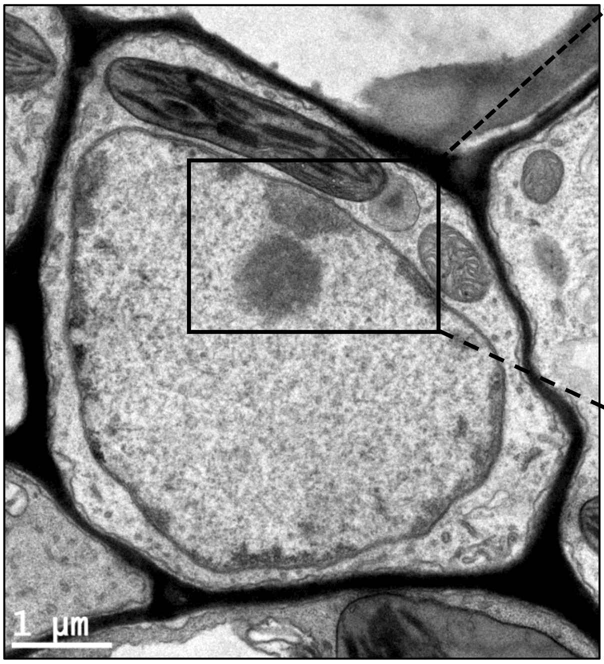

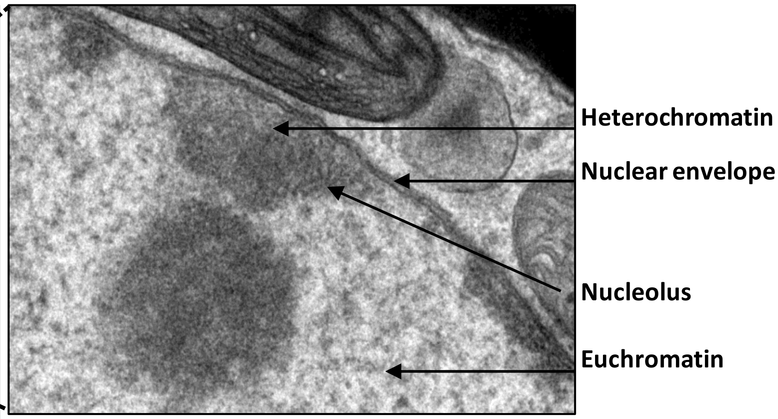

b

Heterochromatin associated to:	
**Nuclear envelope**	**nucleolus**
**100% (n=104)**	**65% (n=60)**

c

Histone mark	Number of observed nuclei	Central	Nuclear envelop
POLII	10	100%	---
H3K4ac	10	100%	---
H3K4me3	10	100%	---
H3K4me2	10	100%	---
H3K4me1	10	100%	---
H3K9ac	10	100%	---
H3K9me2	10	---	100%
H3K14ac	10	100%	---
H3K14me1	10	---	100%
H3K18ac	10	100%	---
H3K23me3	10	100%	---
H3K23me1	10	---	100%
H3K27ac	10	100%	---
H3K27me3	10	100%	---
H3K27me2	10	100%	---
H3K27me1	10	---	100%
H3K36ac	10	100%	---
H3K36me3	10	100%	---
H3K56ac	10	100%	---
H3K56me2	10	---	100%
H3K56me1	10	---	100%
H3K79ac	10	100%	---
H4K5ac	10	100%	---
H4K8ac	10	100%	---
H4K12ac	10	100%	---
H4K16ac	10	100%	---
H4K20ac	10	100%	---

Supplementary Figure 1. (a) Tomato nuclei under transmission electron microscopy. bar=1 μm. Electron micrograph of tomato nuclei with an enlarged section on the right. revealing dense heterochromatin located around the nucleolus and adjacent the nuclear envelope. (b) Heterochromatin sub-localization (associated with the nuclear envelope and nucleolus). In total, 104 nuclei were analyzed. Sixty nuclei were visualized with nucleoli, and 65% of them exhibited associated heterochromatin. (c) Average immunolocalization of 26 histone marks and RNA polymerase II in tomato nuclei. Ten nuclei were observed per mark (n=10).

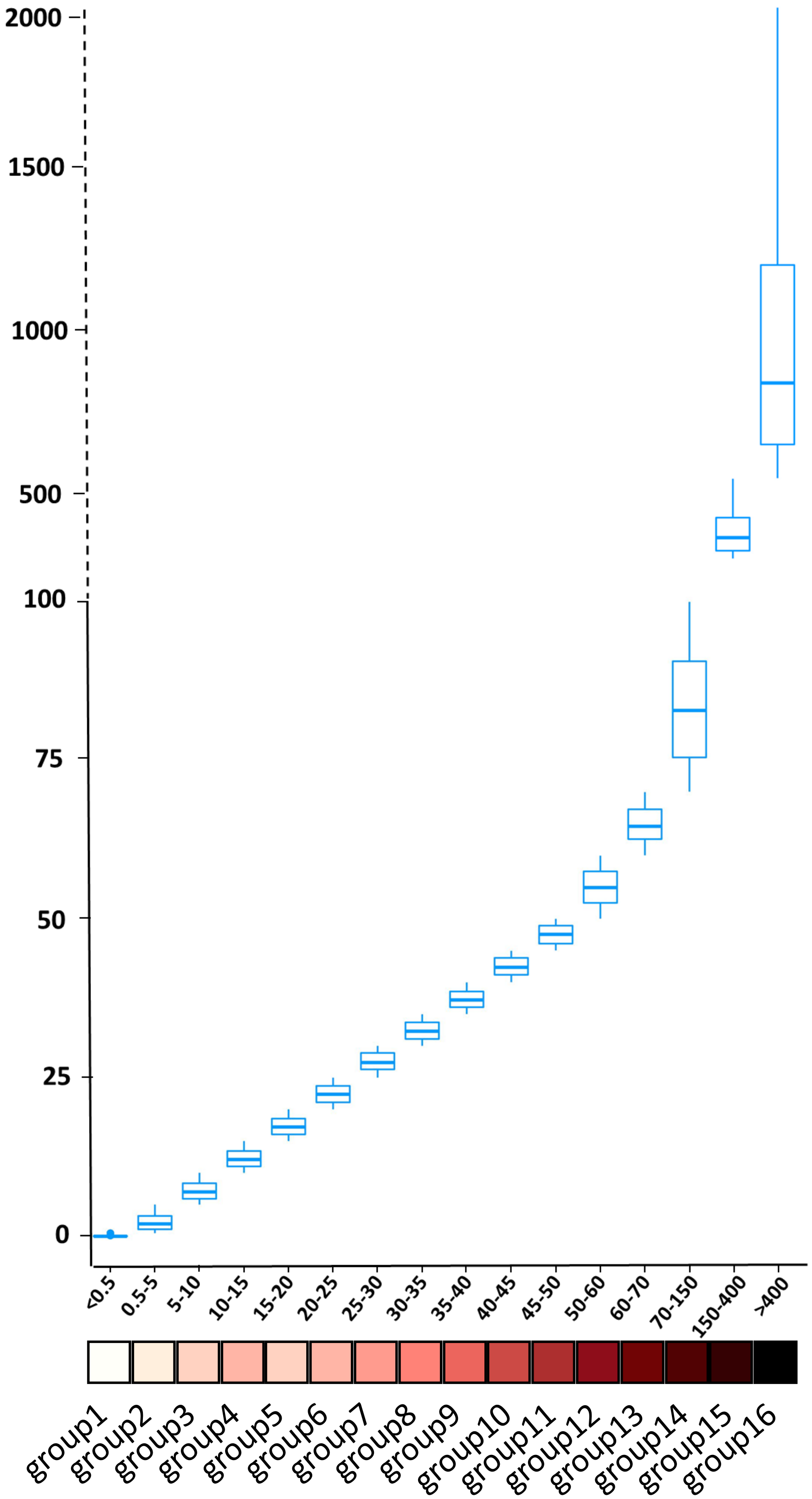

Supplementary Figure 2. Genes classified into 16 groups according to expression levels. Colors scale represented gene expression levels from low (white) to high (red). X axis represents distinct gene expression degrees. Y axis represents gene expression values from tomato WT RNA-seq data coverage of bigwig signals. in which outliers have been excluded.

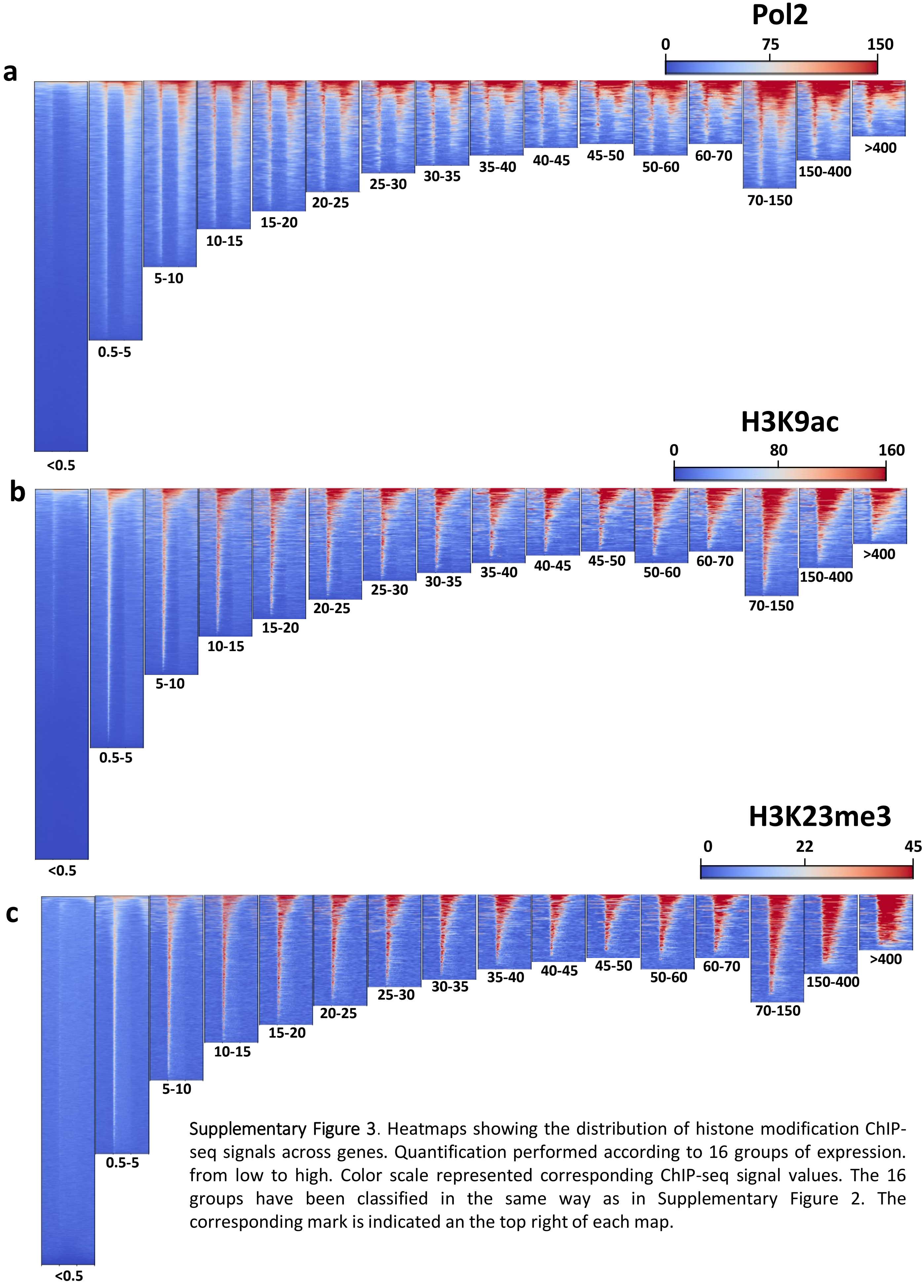

Supplementary Figure 3. Heatmaps showing the distribution of histone modification ChIP-seq signals across genes. Quantification performed according to 16 groups of expression. from low to high. Color scale represented corresponding ChIP-seq signal values. The 16 groups have been classified in the same way as in Supplementary Figure 2. The corresponding mark is indicated an the top right of each map.

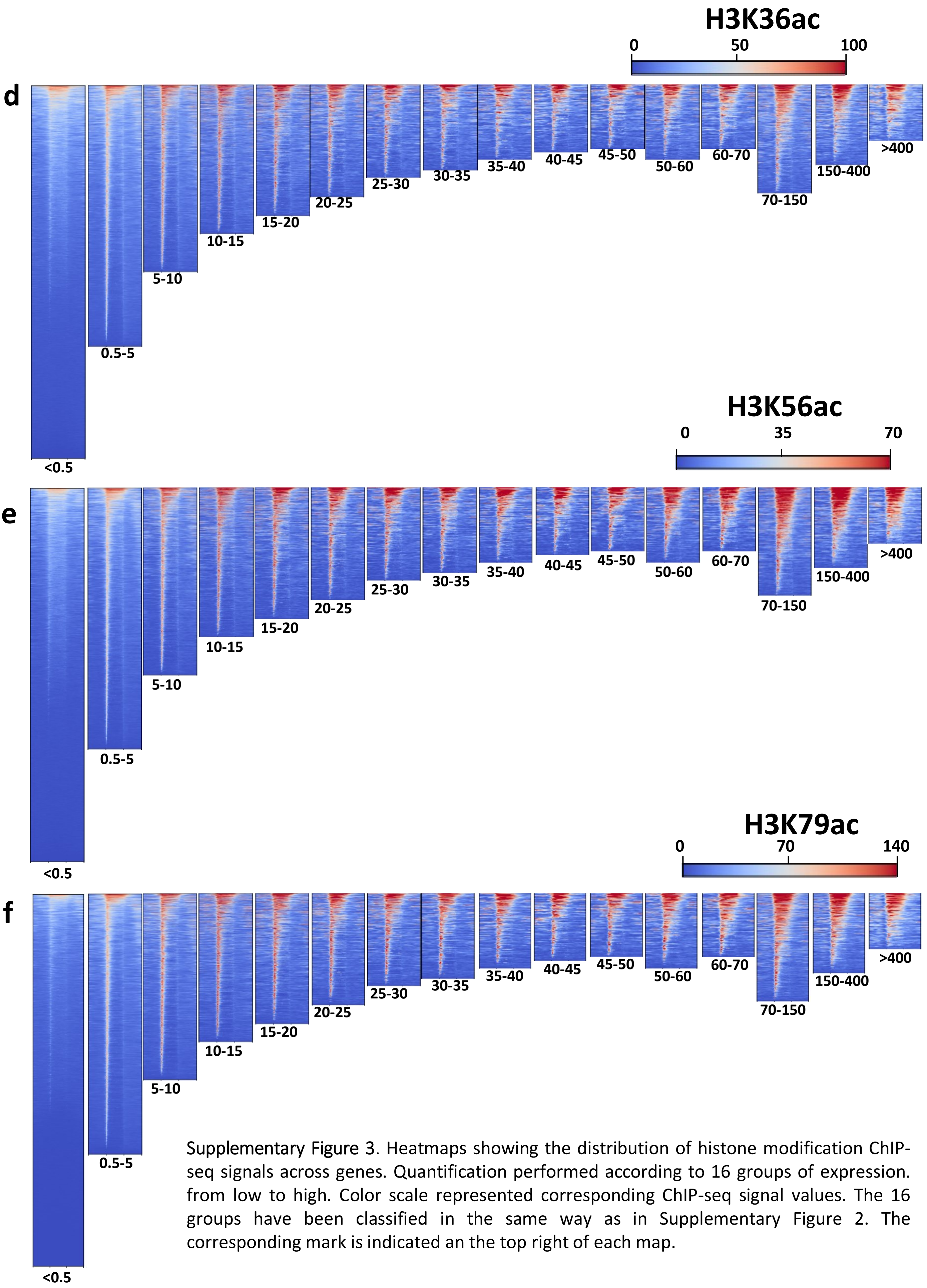

Supplementary Figure 3. Heatmaps showing the distribution of histone modification ChIP-seq signals across genes. Quantification performed according to 16 groups of expression. from low to high. Color scale represented corresponding ChIP-seq signal values. The 16 groups have been classified in the same way as in Supplementary Figure 2. The corresponding mark is indicated an the top right of each map.

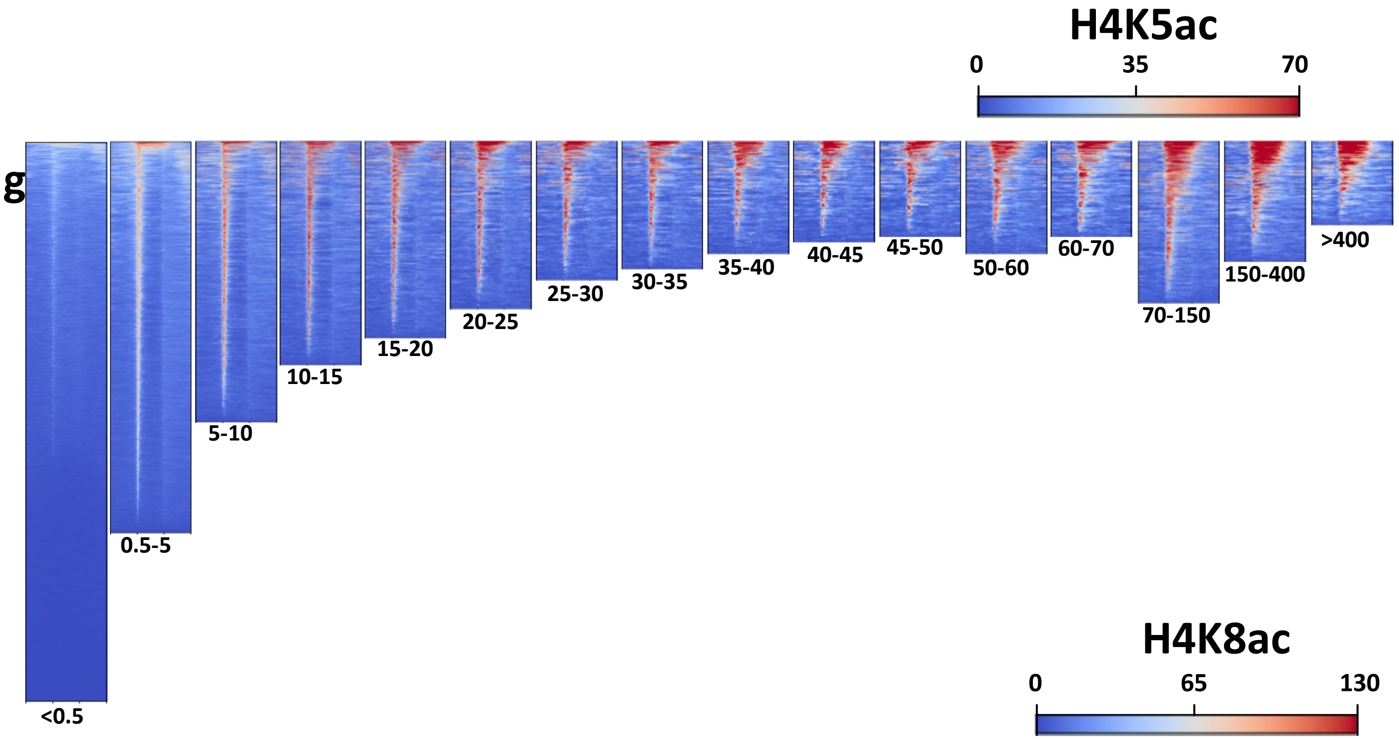

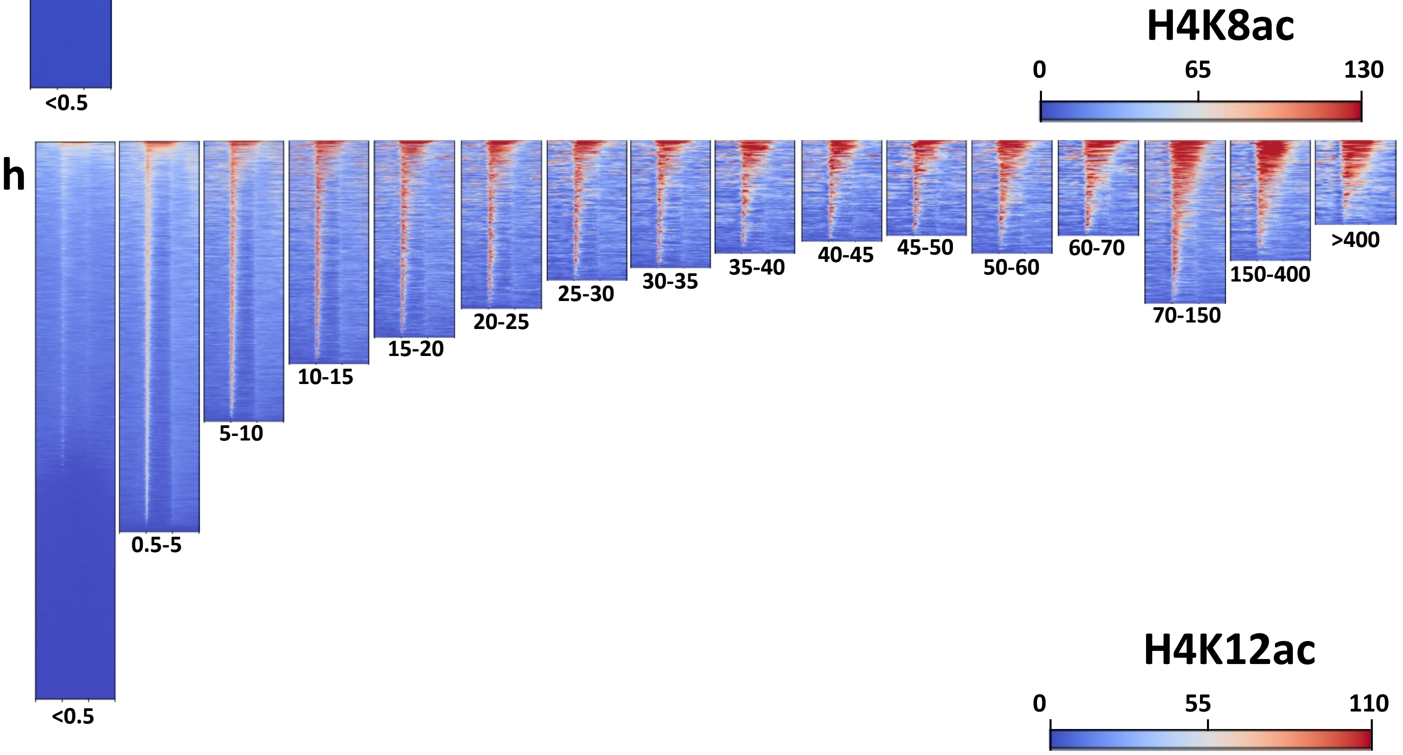

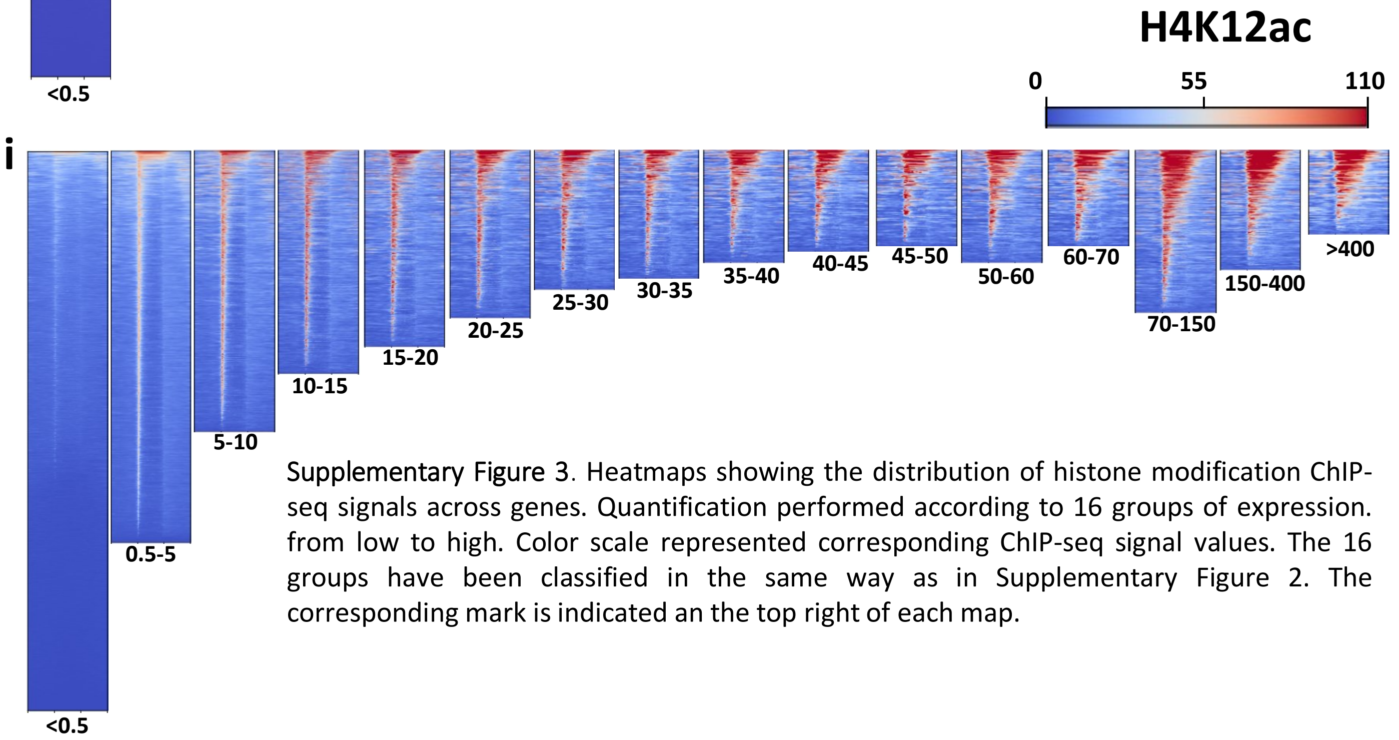

Supplementary Figure 3. Heatmaps showing the distribution of histone modification ChIP-seq signals across genes. Quantification performed according to 16 groups of expression. from low to high. Color scale represented corresponding ChIP-seq signal values. The 16 groups have been classified in the same way as in Supplementary Figure 2. The corresponding mark is indicated an the top right of each map.

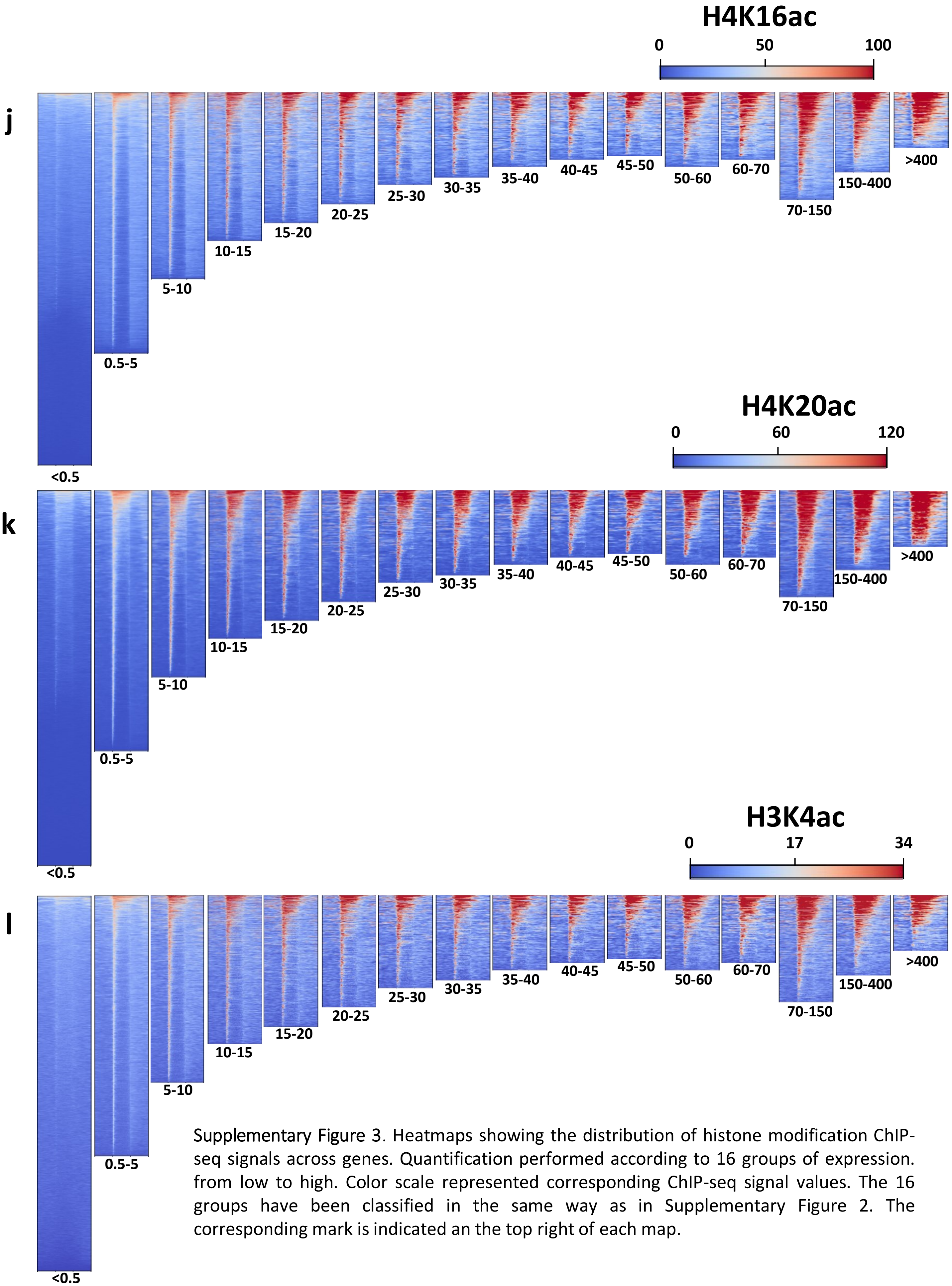

Supplementary Figure 3. Heatmaps showing the distribution of histone modification ChIP-seq signals across genes. Quantification performed according to 16 groups of expression. from low to high. Color scale represented corresponding ChIP-seq signal values. The 16 groups have been classified in the same way as in Supplementary Figure 2. The corresponding mark is indicated an the top right of each map.

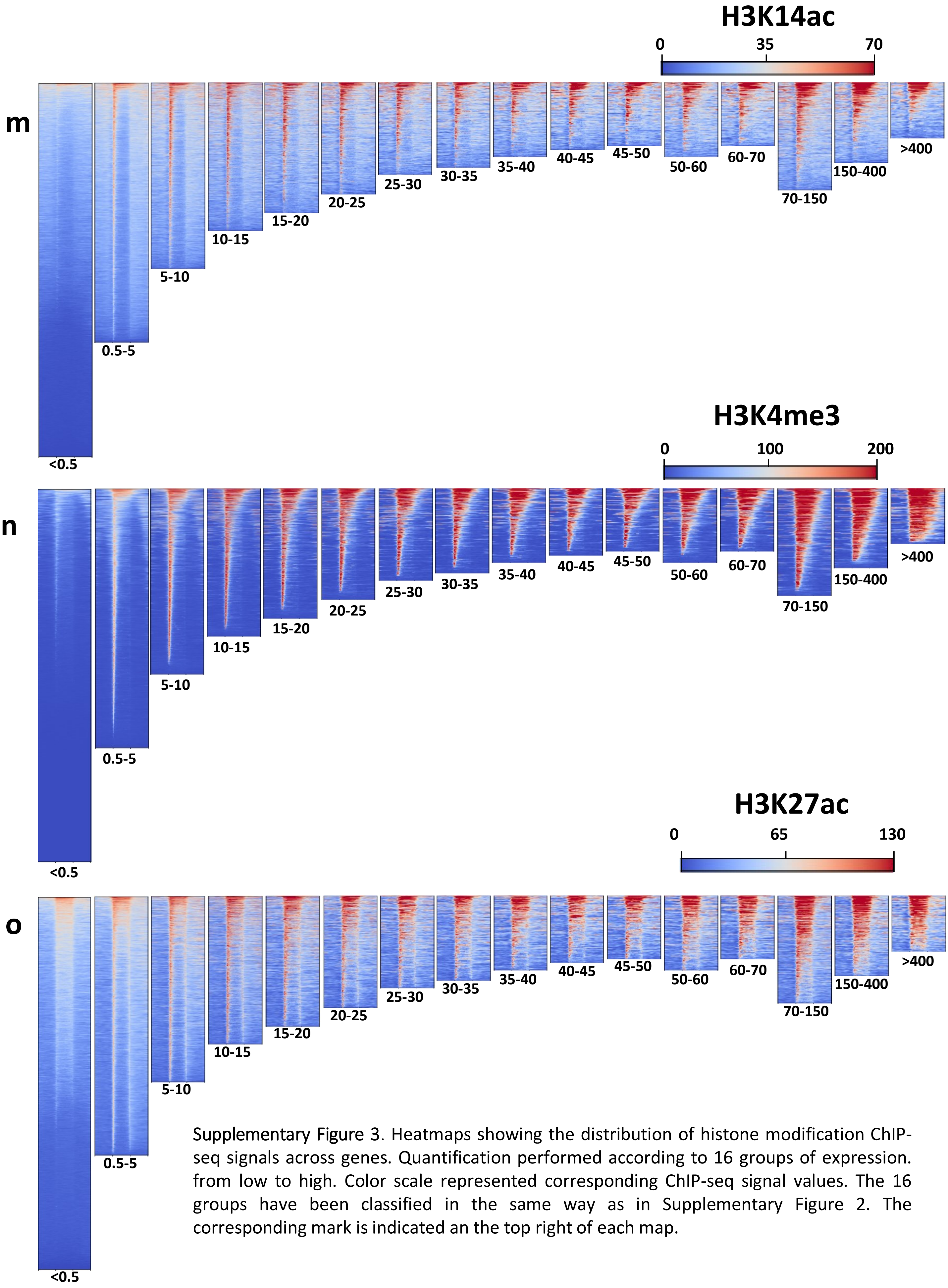

Supplementary Figure 3. Heatmaps showing the distribution of histone modification ChIP-seq signals across genes. Quantification performed according to 16 groups of expression. from low to high. Color scale represented corresponding ChIP-seq signal values. The 16 groups have been classified in the same way as in Supplementary Figure 2. The corresponding mark is indicated an the top right of each map.

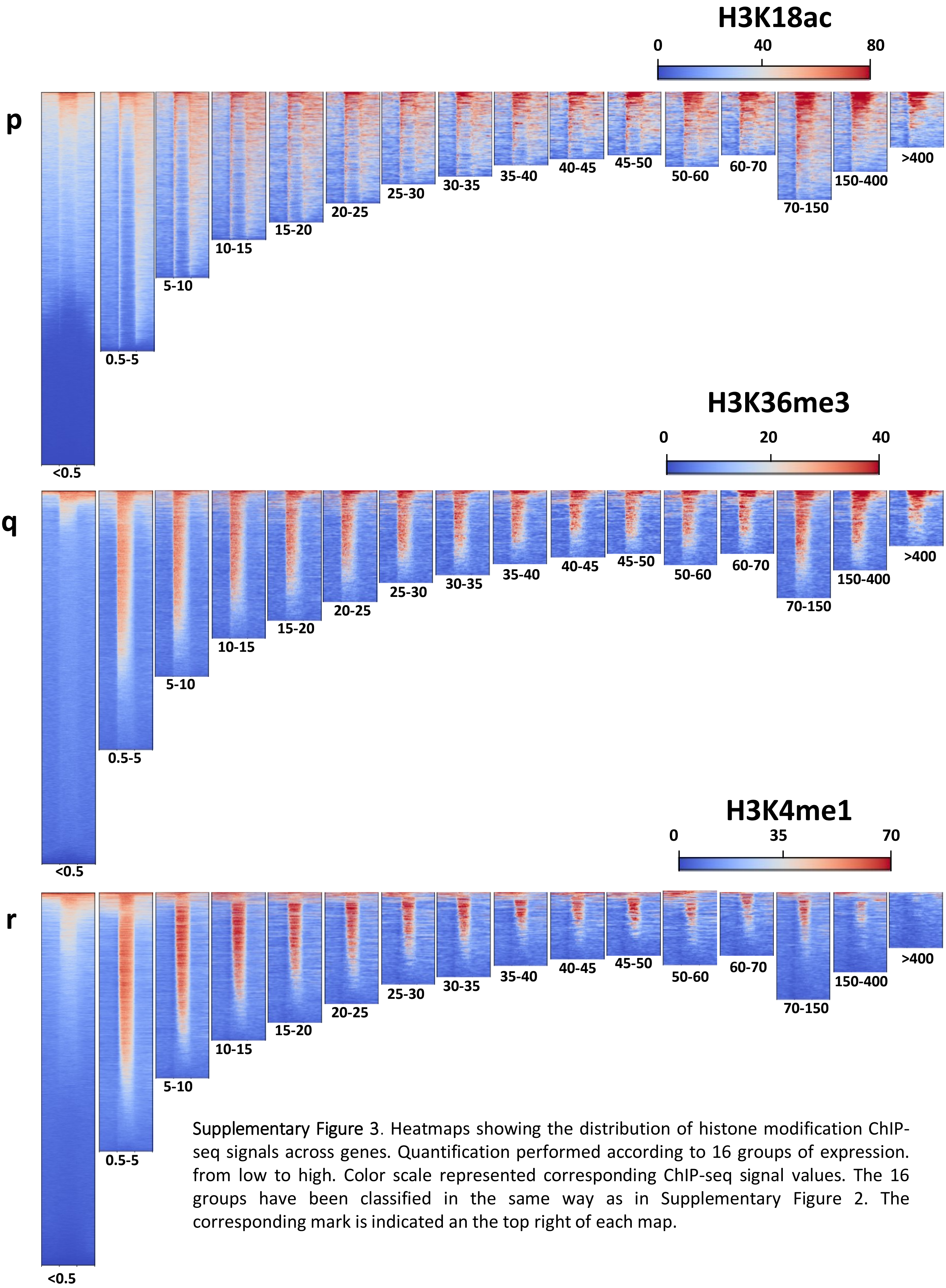

Supplementary Figure 3. Heatmaps showing the distribution of histone modification ChIP-seq signals across genes. Quantification performed according to 16 groups of expression. from low to high. Color scale represented corresponding ChIP-seq signal values. The 16 groups have been classified in the same way as in Supplementary Figure 2. The corresponding mark is indicated an the top right of each map.

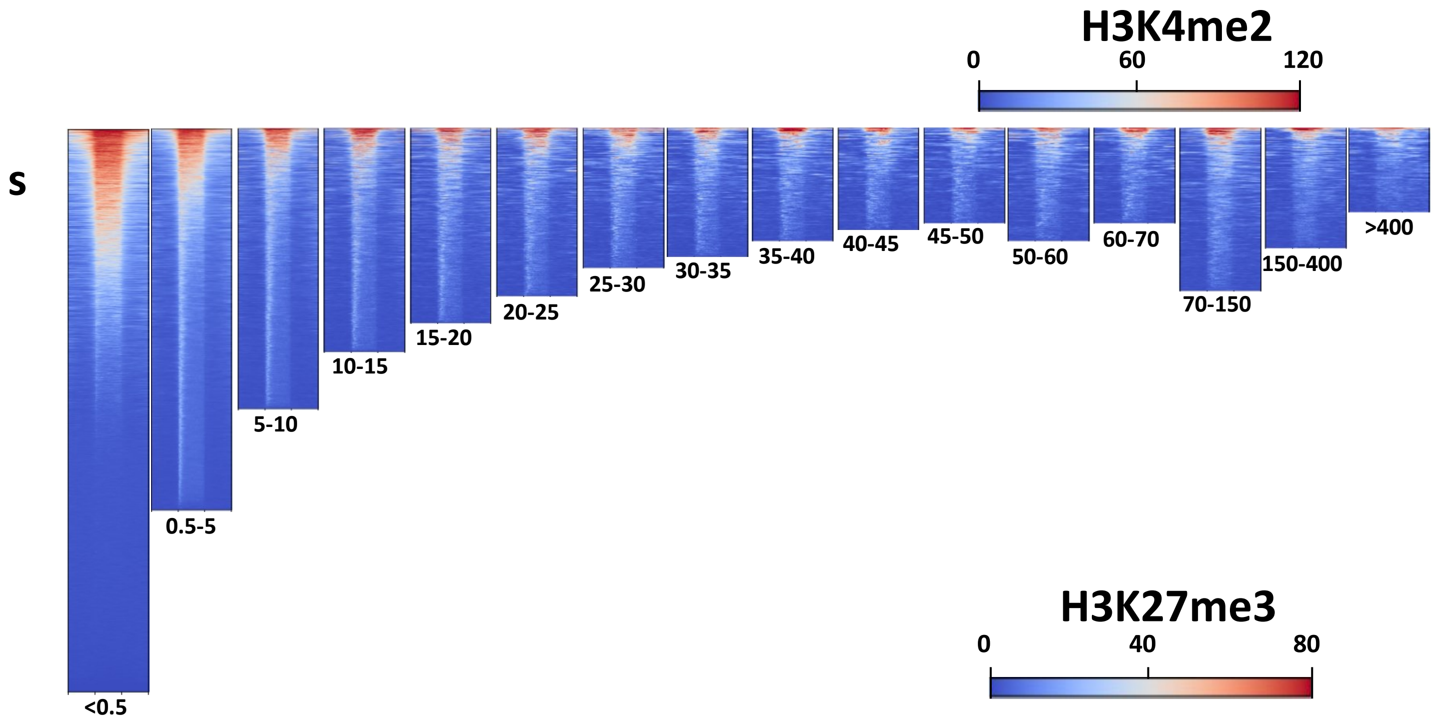

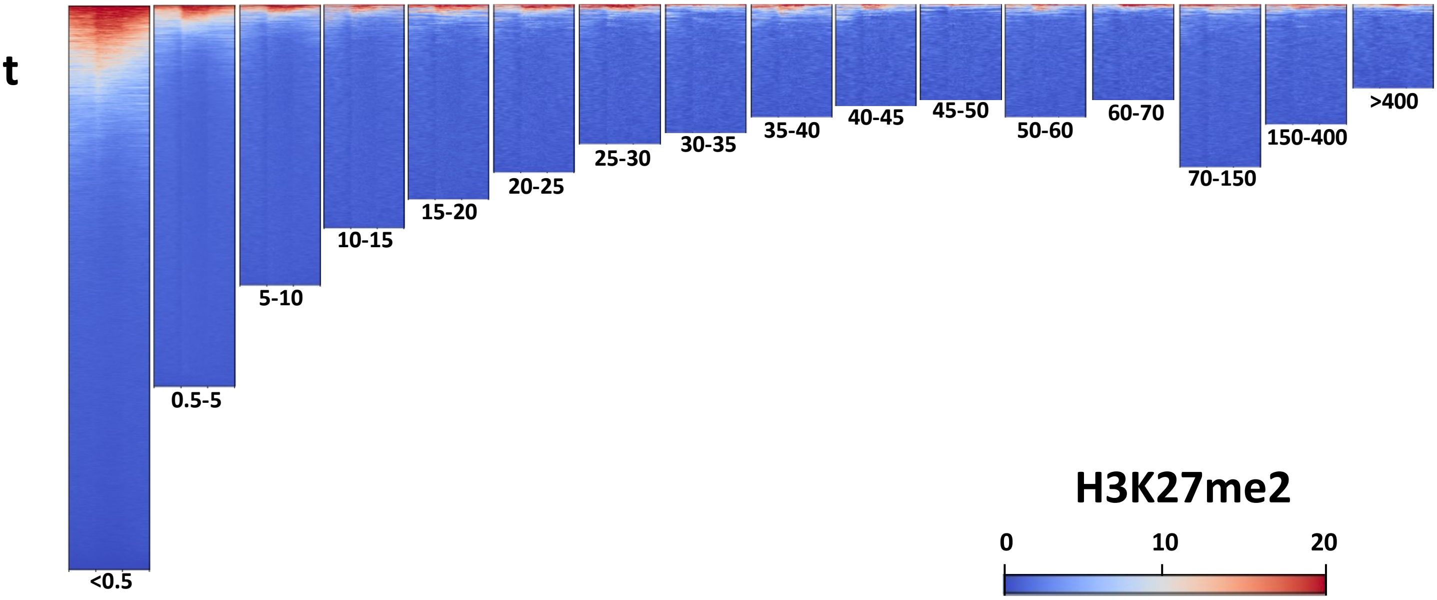

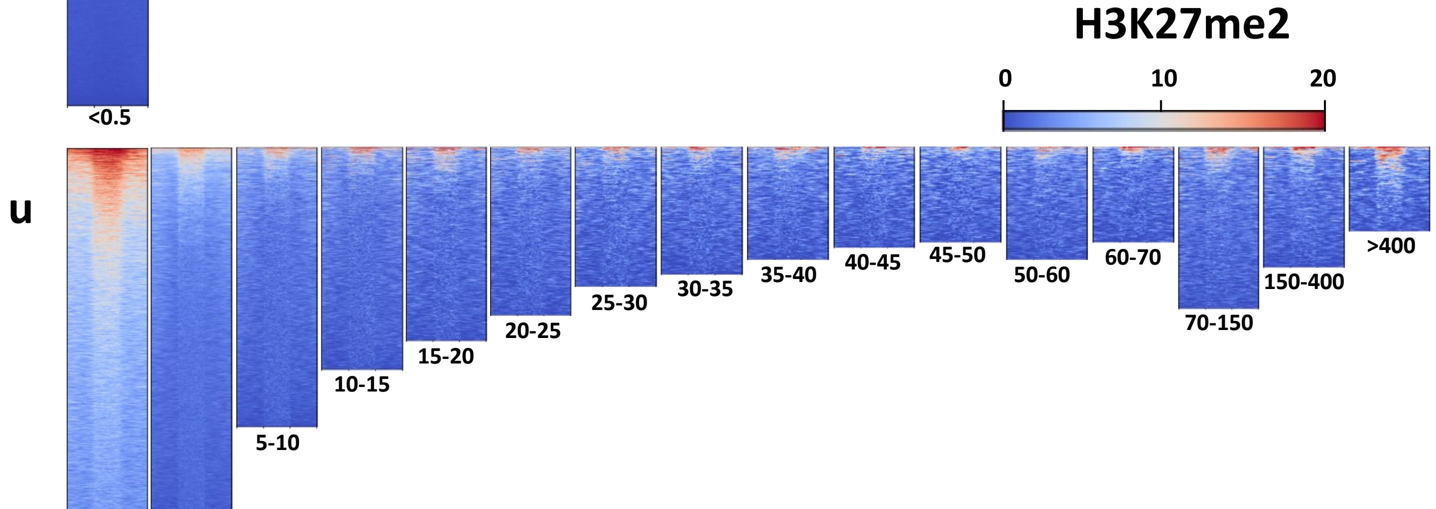

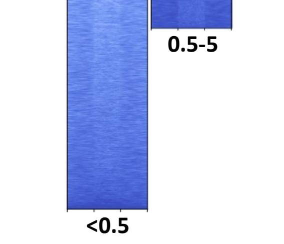

Supplementary Figure 3. Heatmaps showing the distribution of histone modification ChIP-seq signals across genes. Quantification performed according to 16 groups of expression. from low to high. Color scale represented corresponding ChIP-seq signal values. The 16 groups have been classified in the same way as in Supplementary Figure 2. The corresponding mark is indicated an the top right of each map.

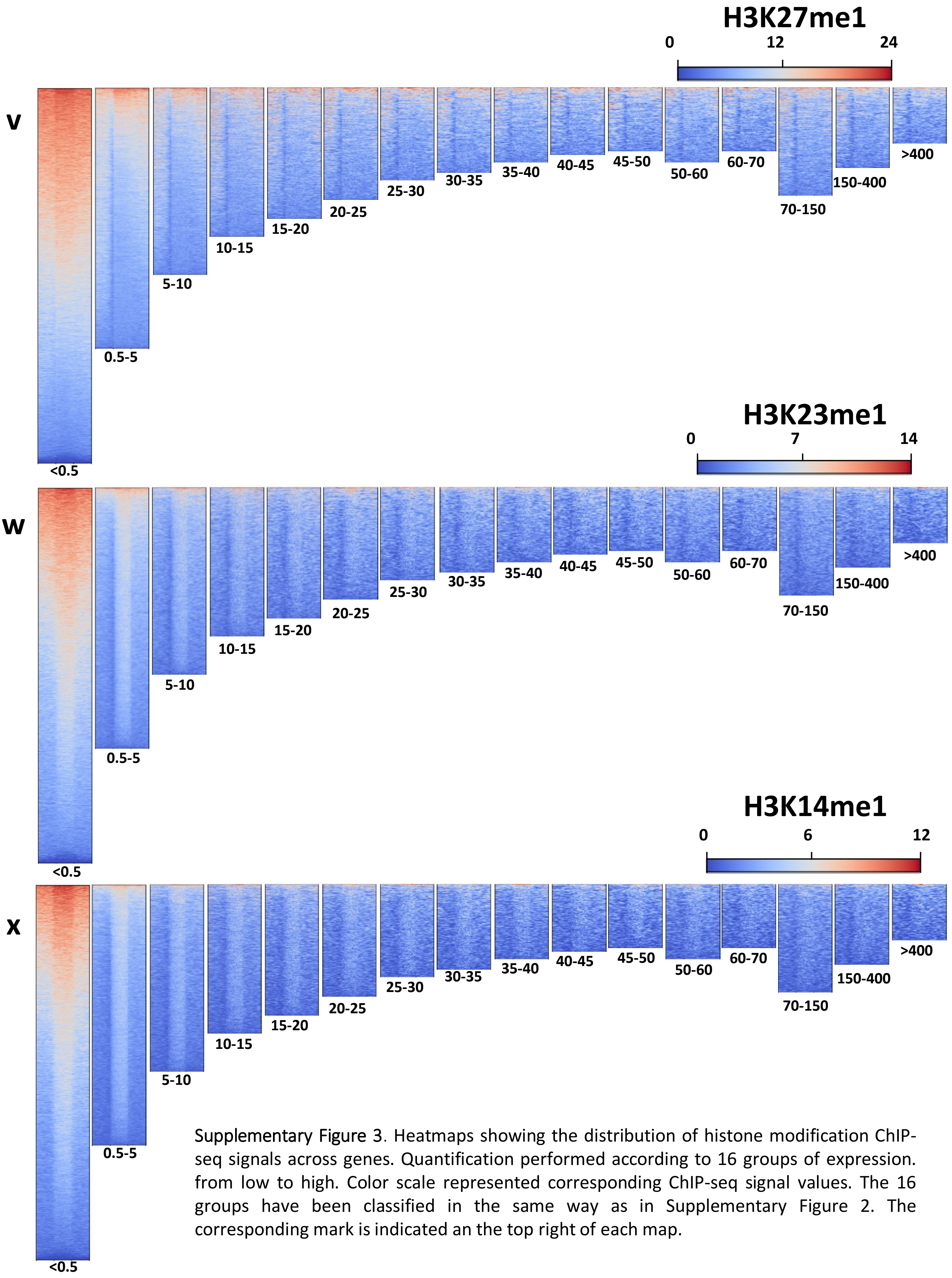

Supplementary Figure 3. Heatmaps showing the distribution of histone modification ChIP-seq signals across genes. Quantification performed according to 16 groups of expression. from low to high. Color scale represented corresponding ChIP-seq signal values. The 16 groups have been classified in the same way as in Supplementary Figure 2. The corresponding mark is indicated an the top right of each map.

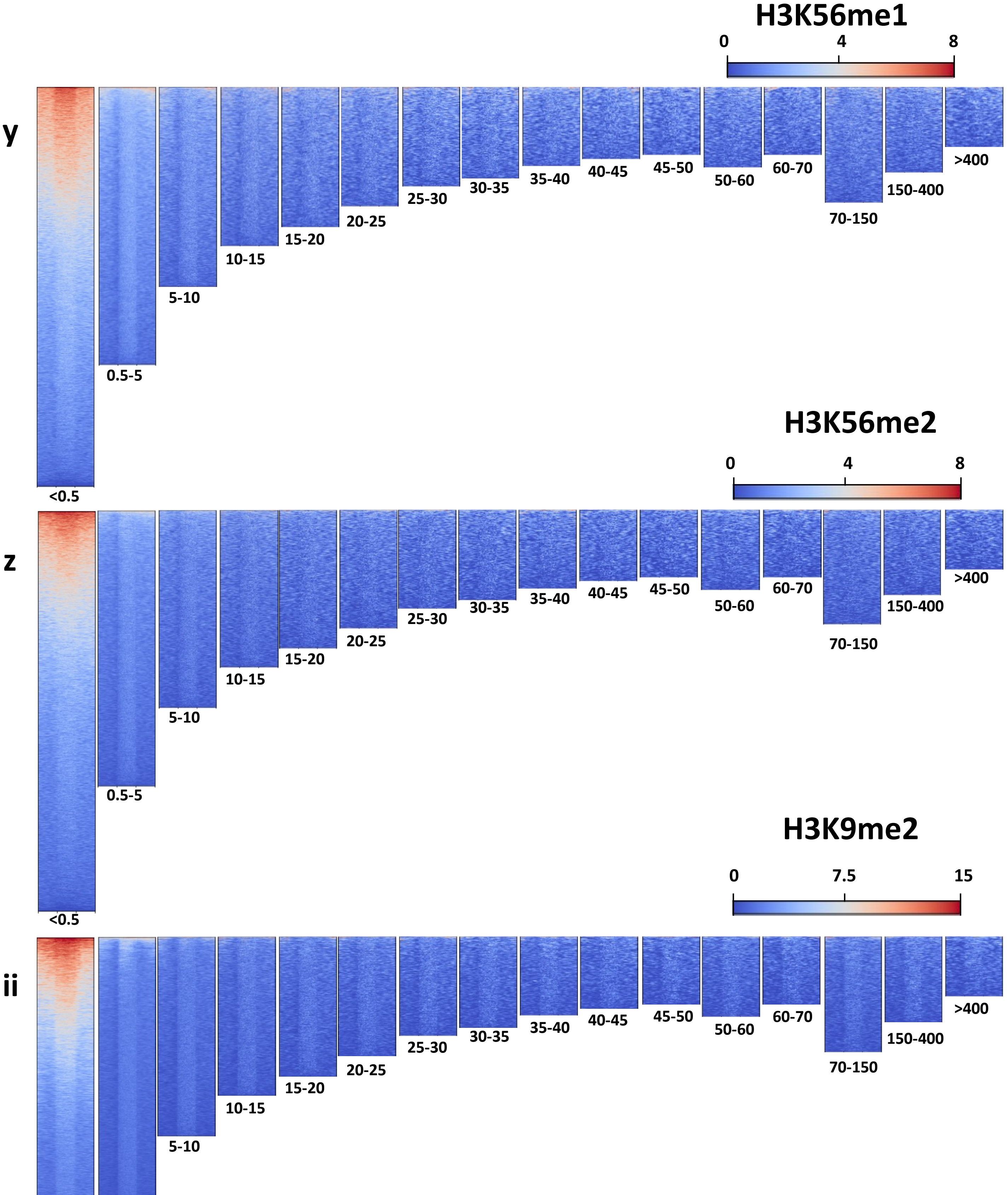

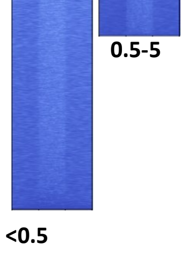

Supplementary Figure 3. Heatmaps showing the distribution of histone modification ChIP-seq signals across genes. Quantification performed according to 16 groups of expression. from low to high. Color scale represented corresponding ChIP-seq signal values. The 16 groups have been classified in the same way as in Supplementary Figure 2. The corresponding mark is indicated an the top right of each map.

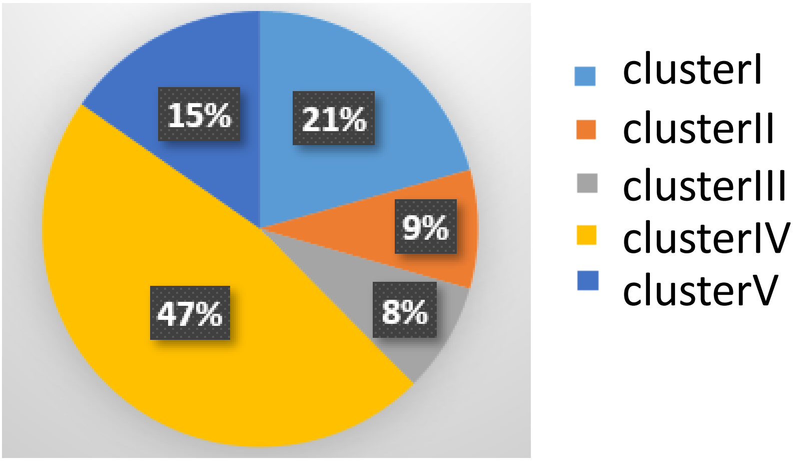

Supplementary Figure 4. Pie chart representing the resulting five different chromatin signatures shown in figure 1e and their corresponding gene proportion.

a

chromatin_state	location_numbers	gene	
E1	15403	13801	0,895994
E2	9032	8485	0,939438
E3	12142	11430	0,941361
E4	10728	7838	0,730611
E5	35232	14477	0,410905
E6	11608	6620	0,570296
E7	12269	10890	0,887603
E8	16247	14092	0,86736
E9	6203	4396	0,708689
E10	10997	6109	0,555515
E11	17904	4255	0,237656
E12	68350	2658	0,038888
E13	50951	1836	0,036035
E14	76080	2890	0,037986
E15	47343	2204	0,046554
E16	89067	9001	0,101059

b

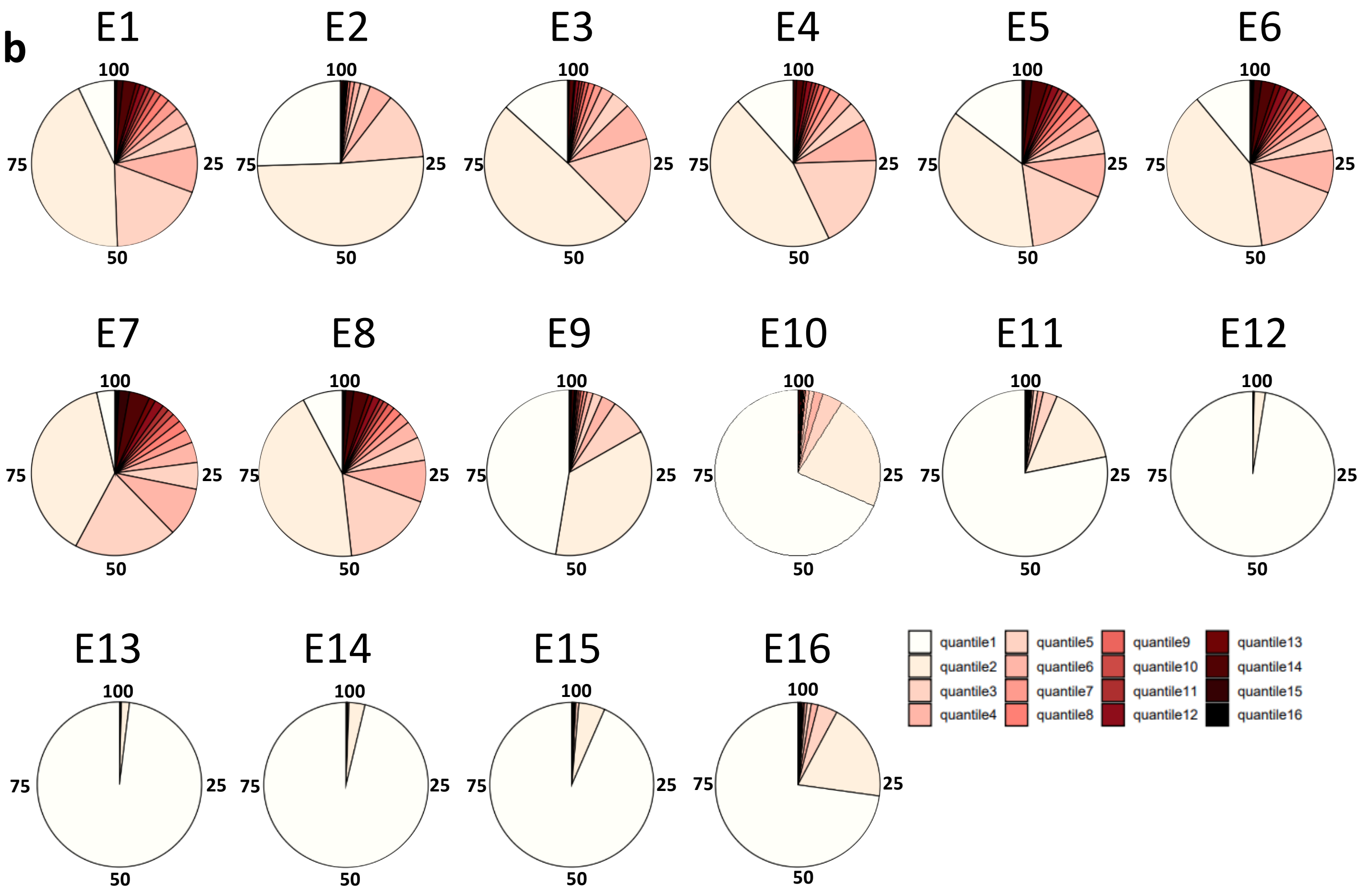

Supplementary Figure 5. Chromatin state gene annotation. (a) Status of the gene coverage from chromatin states. The last column shows the gene proportion covered by each chromatin state. (b) Expression coverage across each chromatin state. Whole genome genes were classified into 16 groups of expression and colors scale represented gene expression levels from low (white) to high (red).

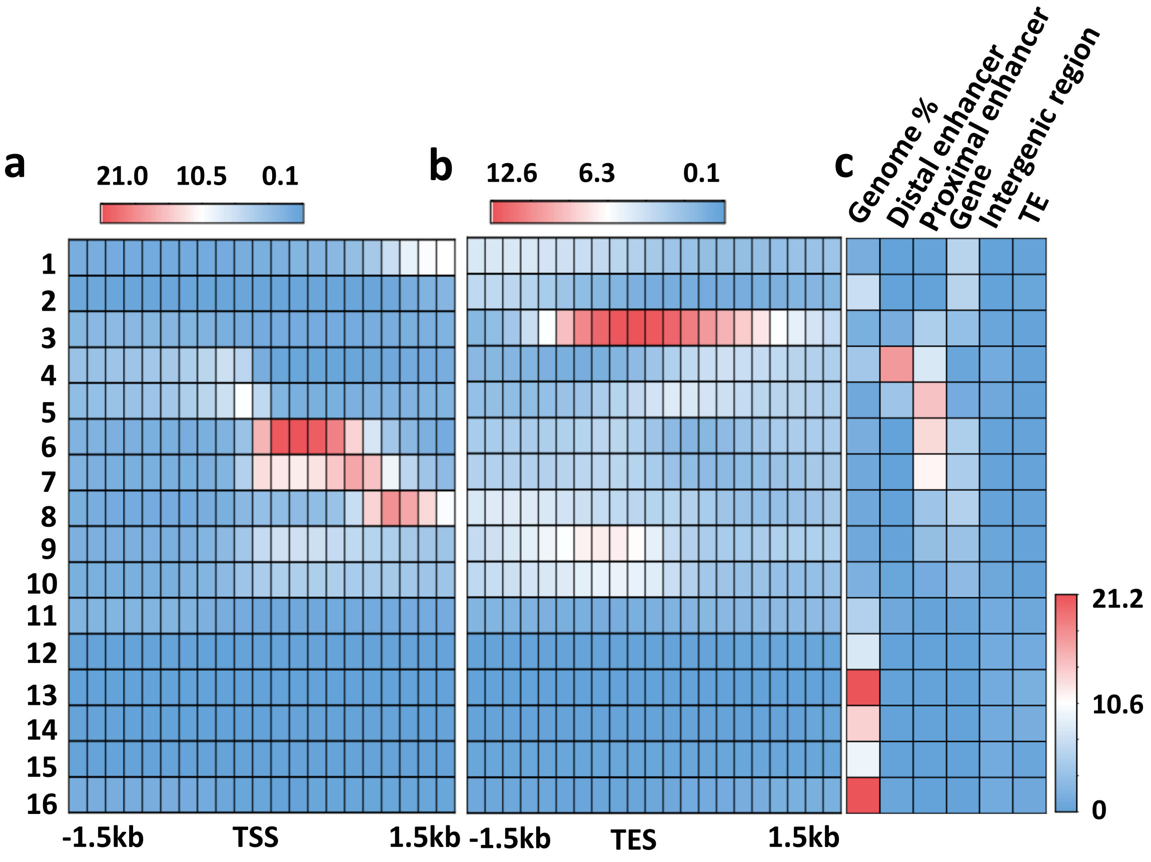

Supplementary Figure 6. Positional enrichments of 16 chromatin states relative to annotated transcription start sites (TSS) (a). transcription end sites (TES) (b) and genomic features (c). Each row represents a specific chromatin state. The color scale denotes enrichment degrees. Positive coordinate values represent the distance downstream in a 5′ to 3′ direction of transcription. while negative values represent the number of bases upstream. Distal and proximal enhancer were identified with ATAC-seq and RNA-seq data; threshold distance of 1Kb from annotated genes.

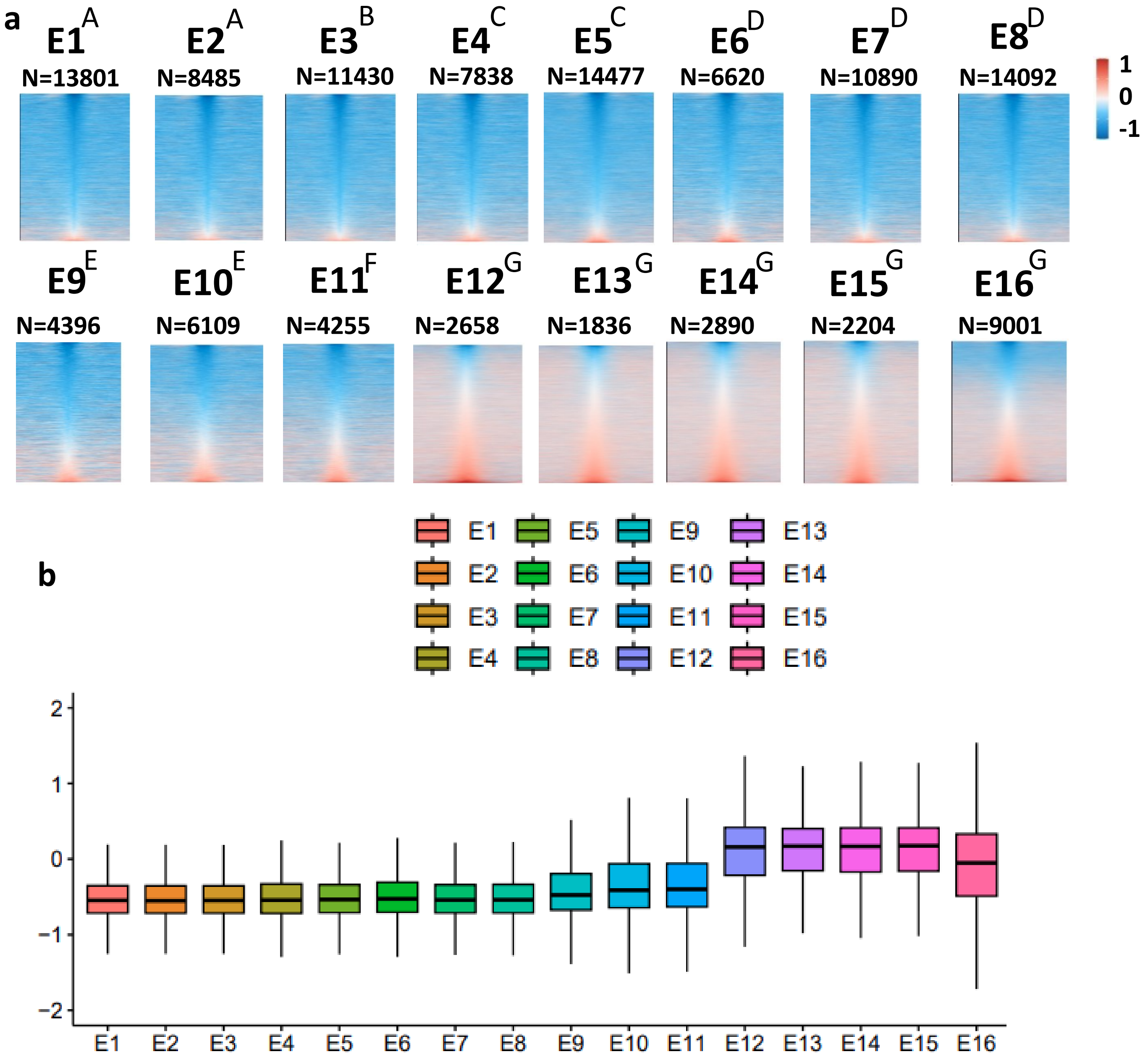

Supplementary Figure 7. Relationship between insulation index and chromatin states. (a) Heatmap presenting the insulation score values of all chromatin states. E stands for chomatin state number. The number above each chromatin state denotes their annotation as follows: A. H3K4me1 controlled genes. B. TES flanking region. C. Active regulatory elements. D. ORFs of actively transcribed genes. E. H3K4me2 repressed genes. F. Polycomb marked loci. G. Constitutive heterochromatin or intergenic region. (b) Box plot representing the median of the insulation index associated to chromatine states. X axis represents different chromatin states and y axis stands for insulation score values. which excluded outliers.

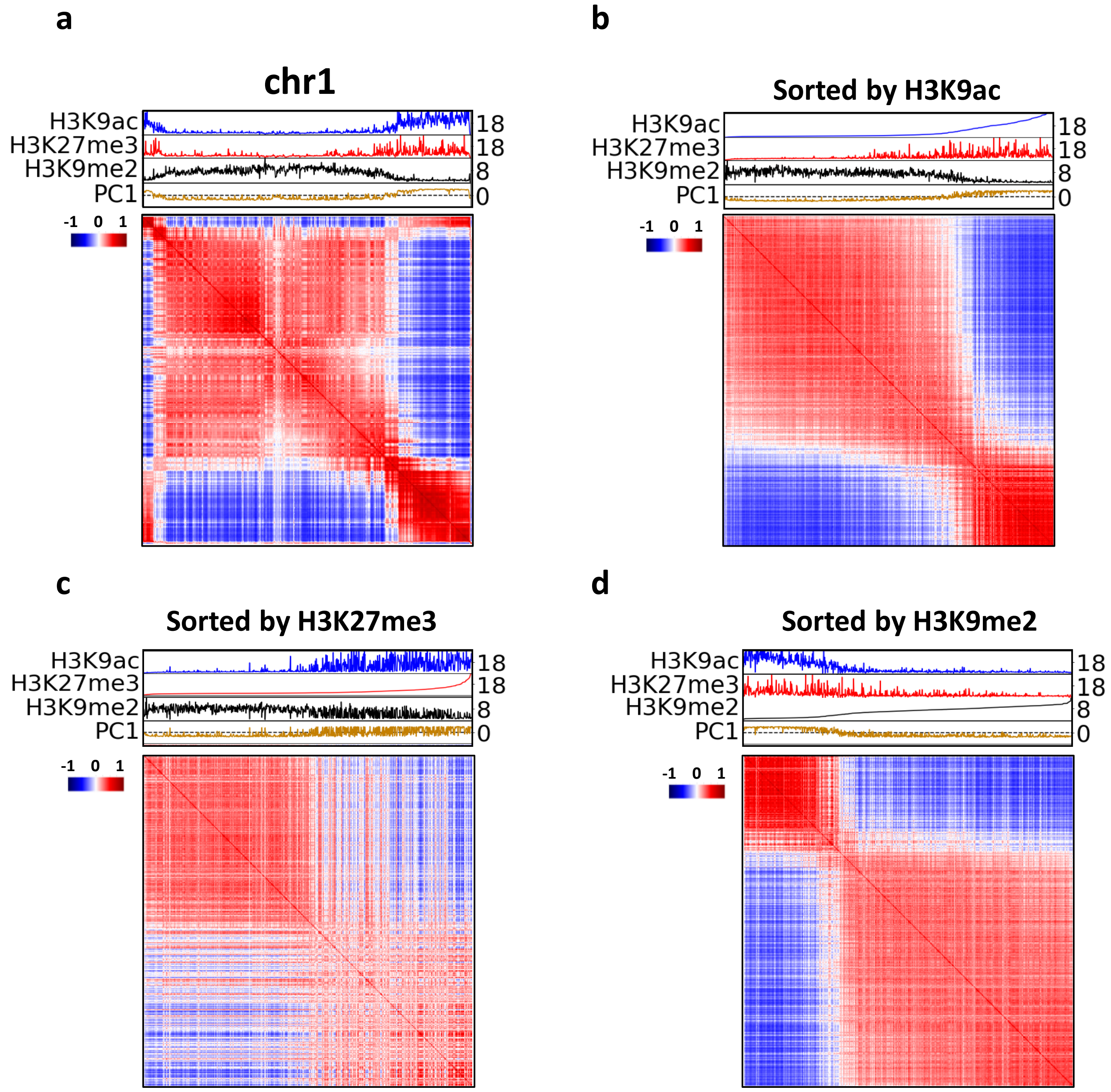

Supplementary Figure 8. Pearson correlations for distance-normalized Hi-C interaction frequency map of chromosomes 1-12. Chromosome 3 is shown in main Figure 2. Shown above each Hi-C map from top to bottom are the following: H3K9ac signal (blue). H3K27me3 signal (red). and H3K9me2 signal (black) and PC1 (brown). For each chromosome from top left to bottom right: (a) Interactions among sequences arranged in natural order. (b) Interactions among sequences sorted by H3K9ac levels. from lowest to highest (c) Interactions among sequences sorted by H3K27me3 levels. from lowest to highest (d) Interactions among sequences sorted by H3K9me2 levels. from lowest to highest.

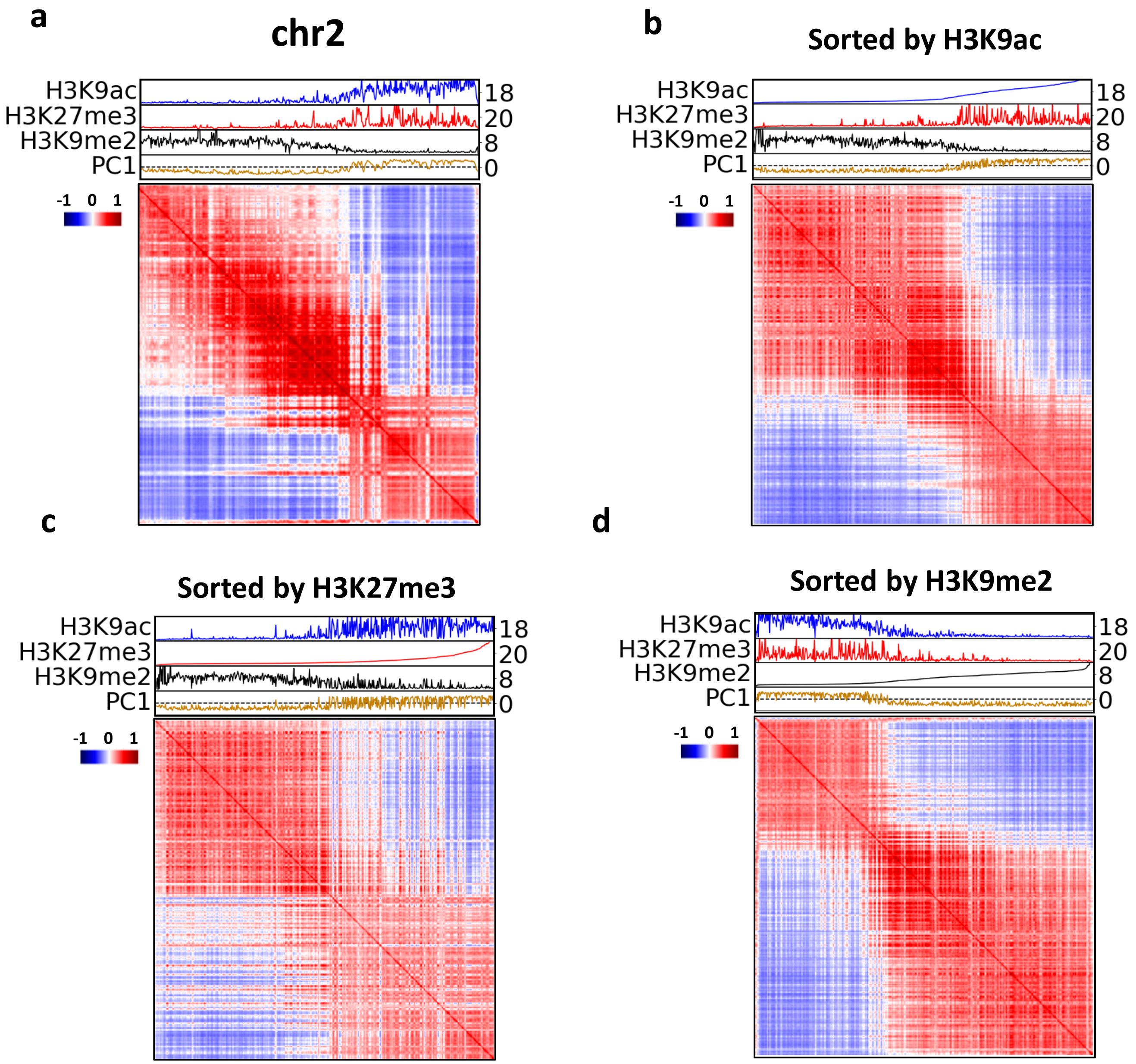

Supplementary Figure 8. Pearson correlations for distance-normalized Hi-C interaction frequency map of chromosomes 1-12. Chromosome 3 is shown in main Figure 2. Shown above each Hi-C map from top to bottom are the following: H3K9ac signal (blue). H3K27me3 signal (red). and H3K9me2 signal (black) and PC1 (brown). For each chromosome from top left to bottom right: (a) Interactions among sequences arranged in natural order. (b) Interactions among sequences sorted by H3K9ac levels. from lowest to highest (c) Interactions among sequences sorted by H3K27me3 levels. from lowest to highest (d) Interactions among sequences sorted by H3K9me2 levels. from lowest to highest.

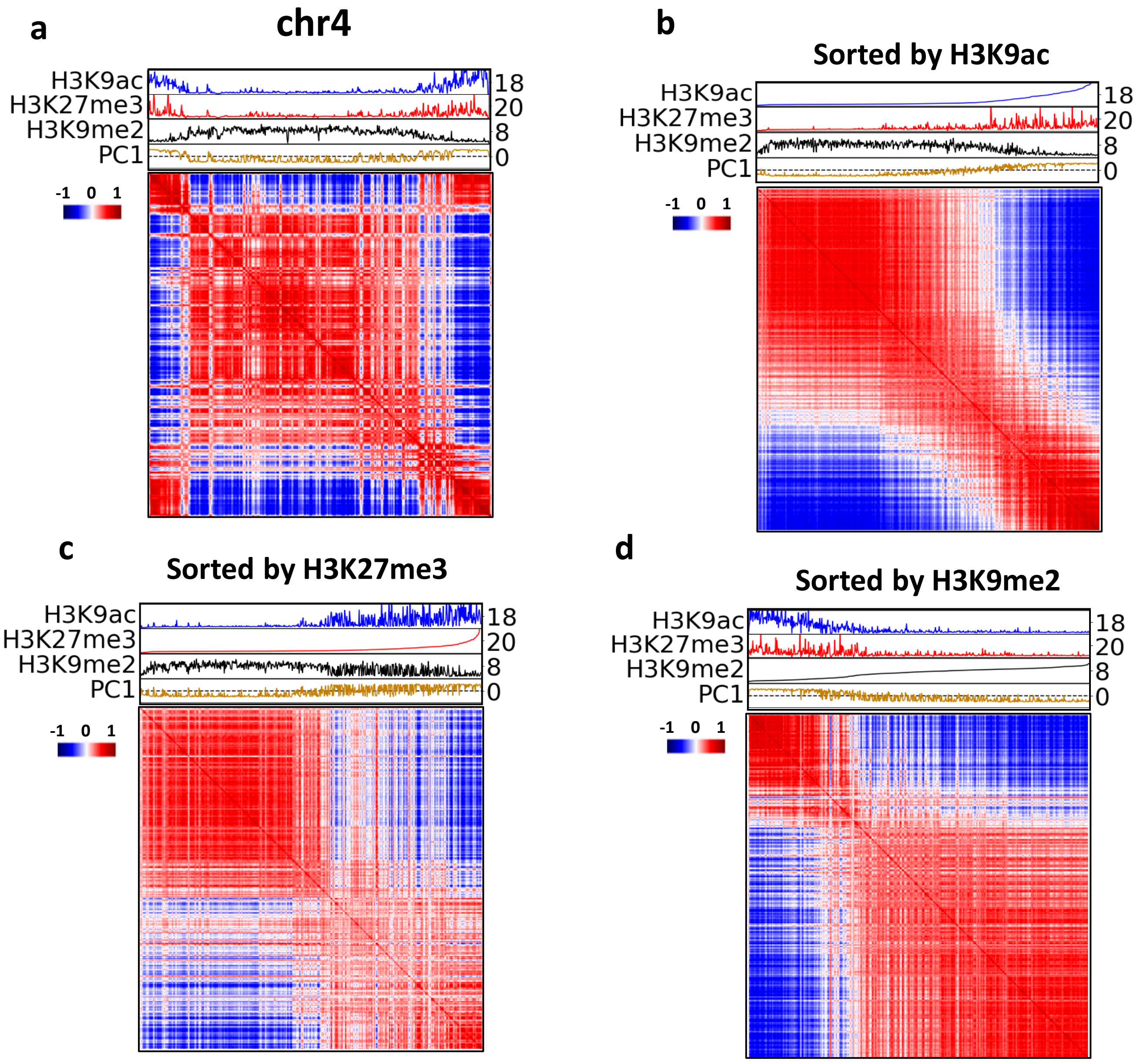

Supplementary Figure 8. Pearson correlations for distance-normalized Hi-C interaction frequency map of chromosomes 1-12. Chromosome 3 is shown in main Figure 2. Shown above each Hi-C map from top to bottom are the following: H3K9ac signal (blue). H3K27me3 signal (red). and H3K9me2 signal (black) and PC1 (brown). For each chromosome from top left to bottom right: (a) Interactions among sequences arranged in natural order. (b) Interactions among sequences sorted by H3K9ac levels. from lowest to highest (c) Interactions among sequences sorted by H3K27me3 levels. from lowest to highest (d) Interactions among sequences sorted by H3K9me2 levels. from lowest to highest.

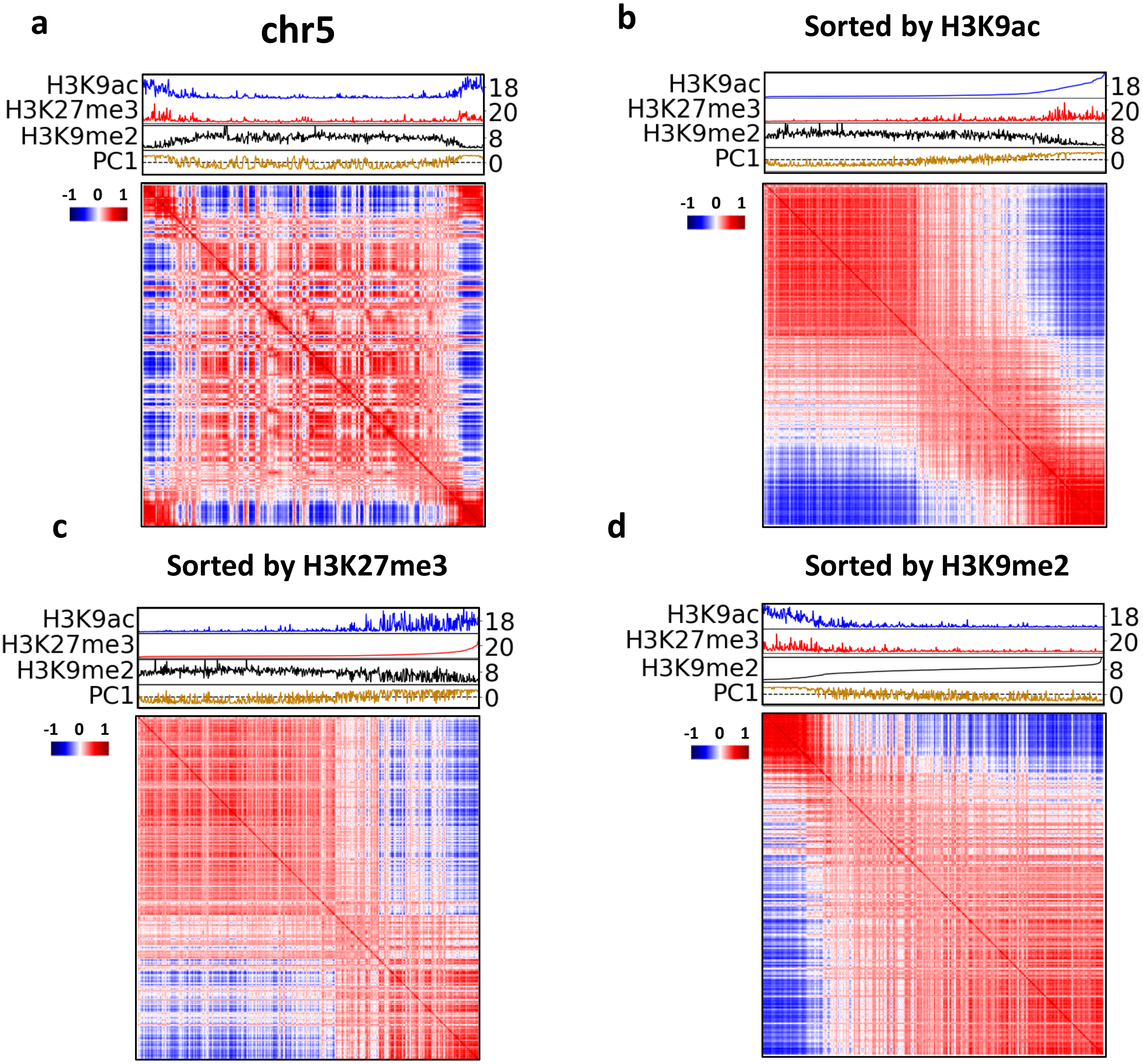

Supplementary Figure 8. Pearson correlations for distance-normalized Hi-C interaction frequency map of chromosomes 1-12. Chromosome 3 is shown in main Figure 2. Shown above each Hi-C map from top to bottom are the following: H3K9ac signal (blue). H3K27me3 signal (red). and H3K9me2 signal (black) and PC1 (brown). For each chromosome from top left to bottom right: (a) Interactions among sequences arranged in natural order. (b) Interactions among sequences sorted by H3K9ac levels. from lowest to highest (c) Interactions among sequences sorted by H3K27me3 levels. from lowest to highest (d) Interactions among sequences sorted by H3K9me2 levels. from lowest to highest.

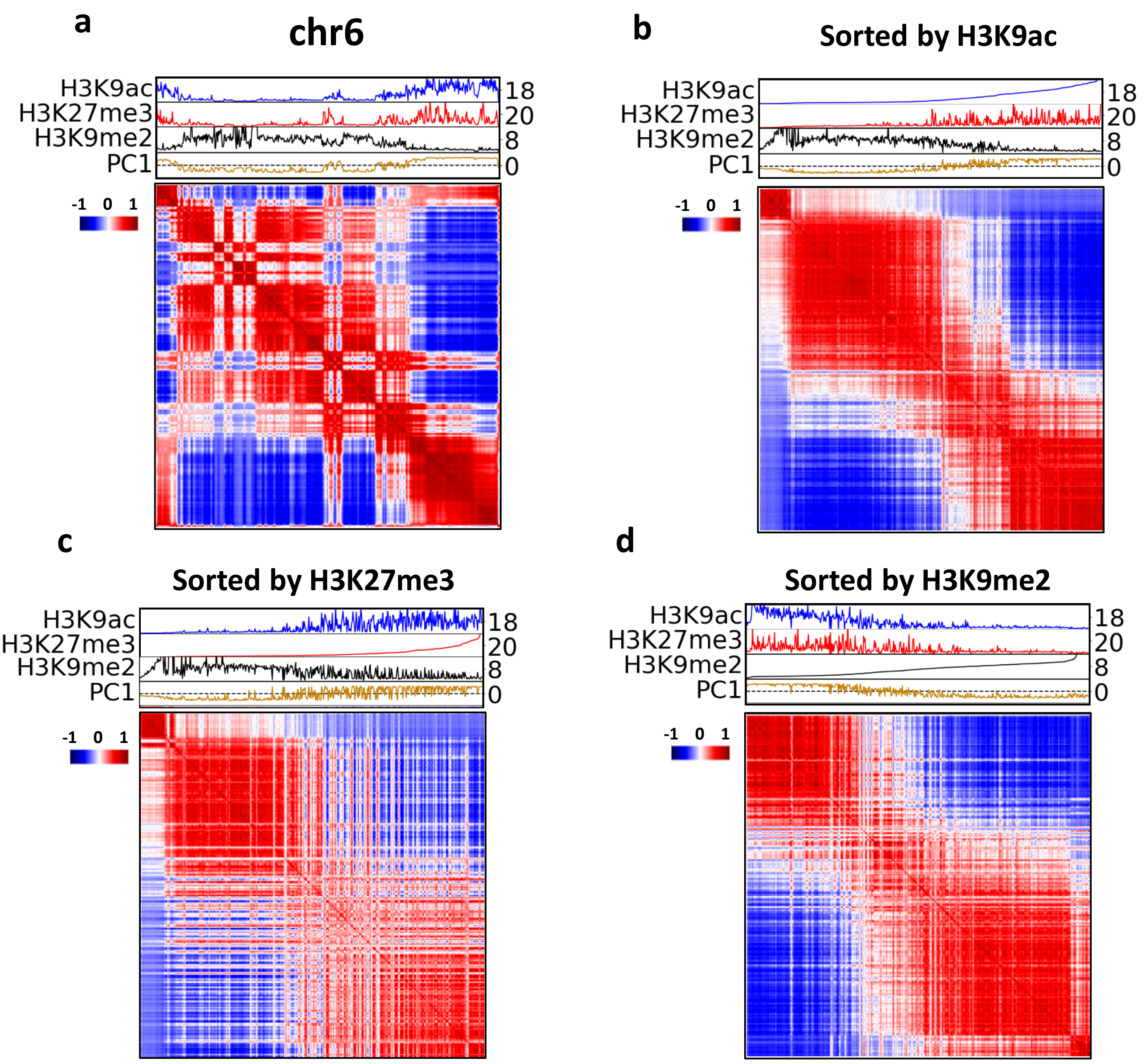

Supplementary Figure 8. Pearson correlations for distance-normalized Hi-C interaction frequency map of chromosomes 1-12. Chromosome 3 is shown in main Figure 2. Shown above each Hi-C map from top to bottom are the following: H3K9ac signal (blue). H3K27me3 signal (red). and H3K9me2 signal (black) and PC1 (brown). For each chromosome from top left to bottom right: (a) Interactions among sequences arranged in natural order. (b) Interactions among sequences sorted by H3K9ac levels. from lowest to highest (c) Interactions among sequences sorted by H3K27me3 levels. from lowest to highest (d) Interactions among sequences sorted by H3K9me2 levels. from lowest to highest.

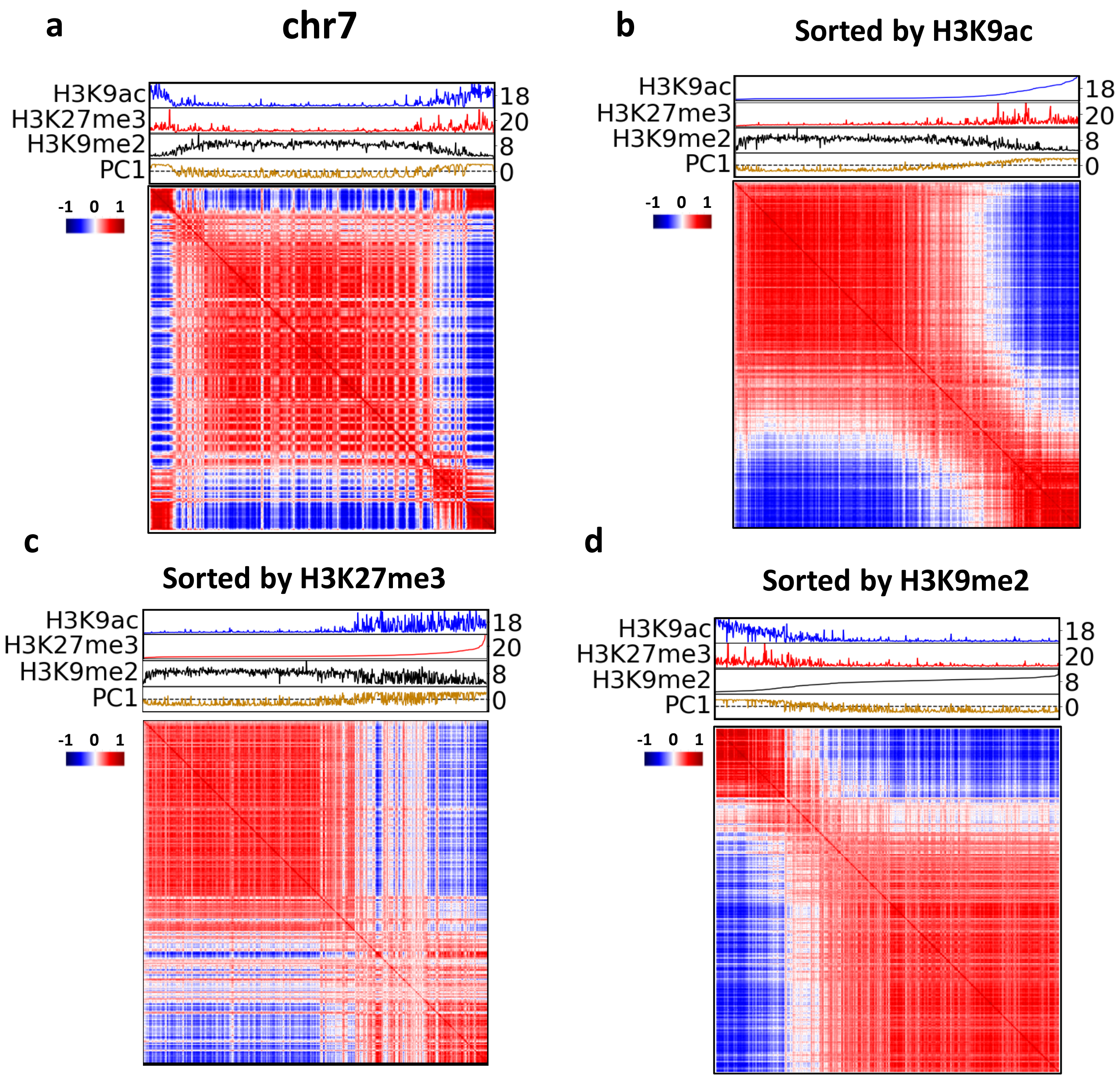

Supplementary Figure 8. Pearson correlations for distance-normalized Hi-C interaction frequency map of chromosomes 1-12. Chromosome 3 is shown in main Figure 2. Shown above each Hi-C map from top to bottom are the following: H3K9ac signal (blue). H3K27me3 signal (red). and H3K9me2 signal (black) and PC1 (brown). For each chromosome from top left to bottom right: (a) Interactions among sequences arranged in natural order. (b) Interactions among sequences sorted by H3K9ac levels. from lowest to highest (c) Interactions among sequences sorted by H3K27me3 levels. from lowest to highest (d) Interactions among sequences sorted by H3K9me2 levels. from lowest to highest.

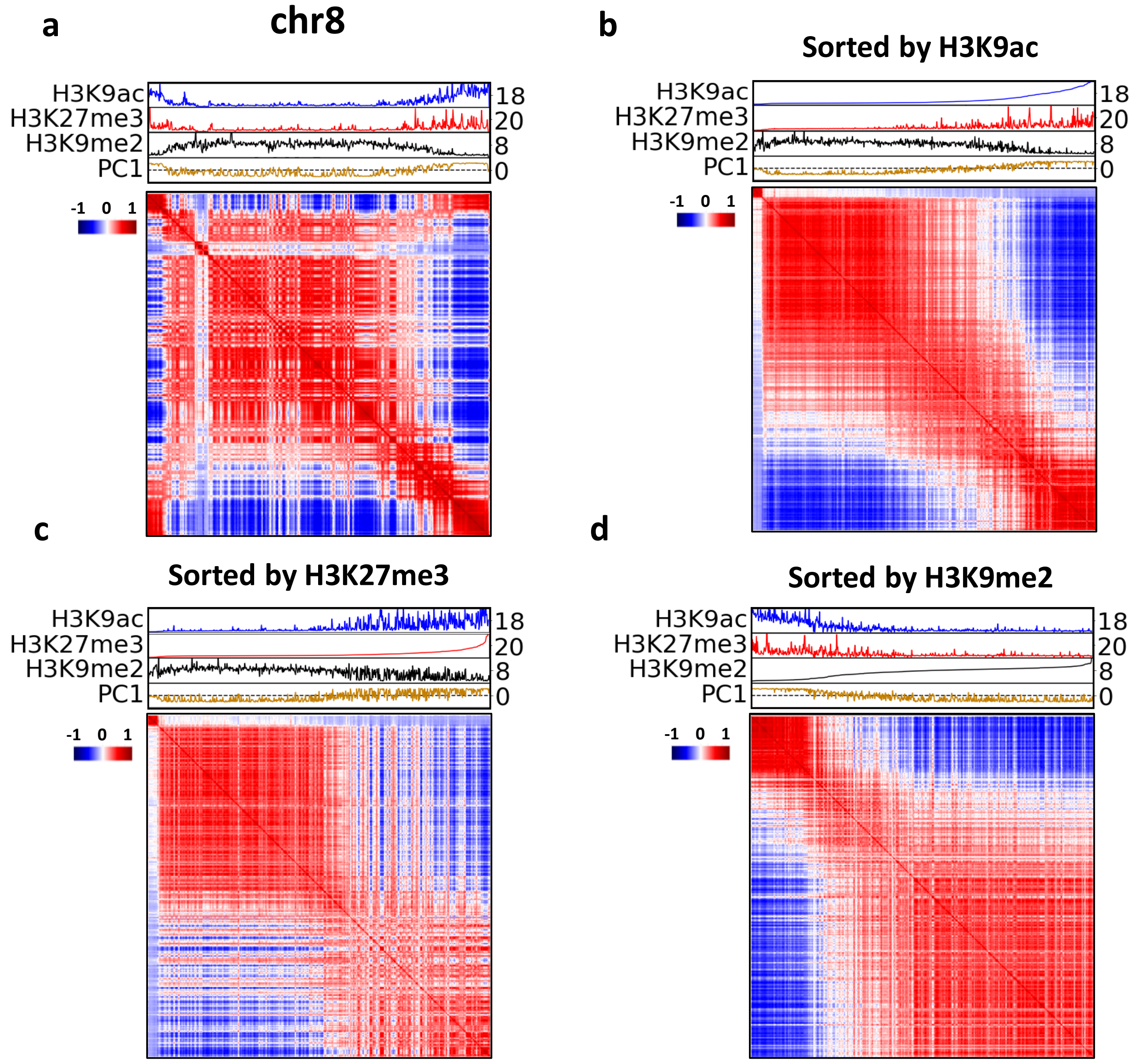

Supplementary Figure 8. Pearson correlations for distance-normalized Hi-C interaction frequency map of chromosomes 1-12. Chromosome 3 is shown in main Figure 2. Shown above each Hi-C map from top to bottom are the following: H3K9ac signal (blue). H3K27me3 signal (red). and H3K9me2 signal (black) and PC1 (brown). For each chromosome from top left to bottom right: (a) Interactions among sequences arranged in natural order. (b) Interactions among sequences sorted by H3K9ac levels. from lowest to highest (c) Interactions among sequences sorted by H3K27me3 levels. from lowest to highest (d) Interactions among sequences sorted by H3K9me2 levels. from lowest to highest.

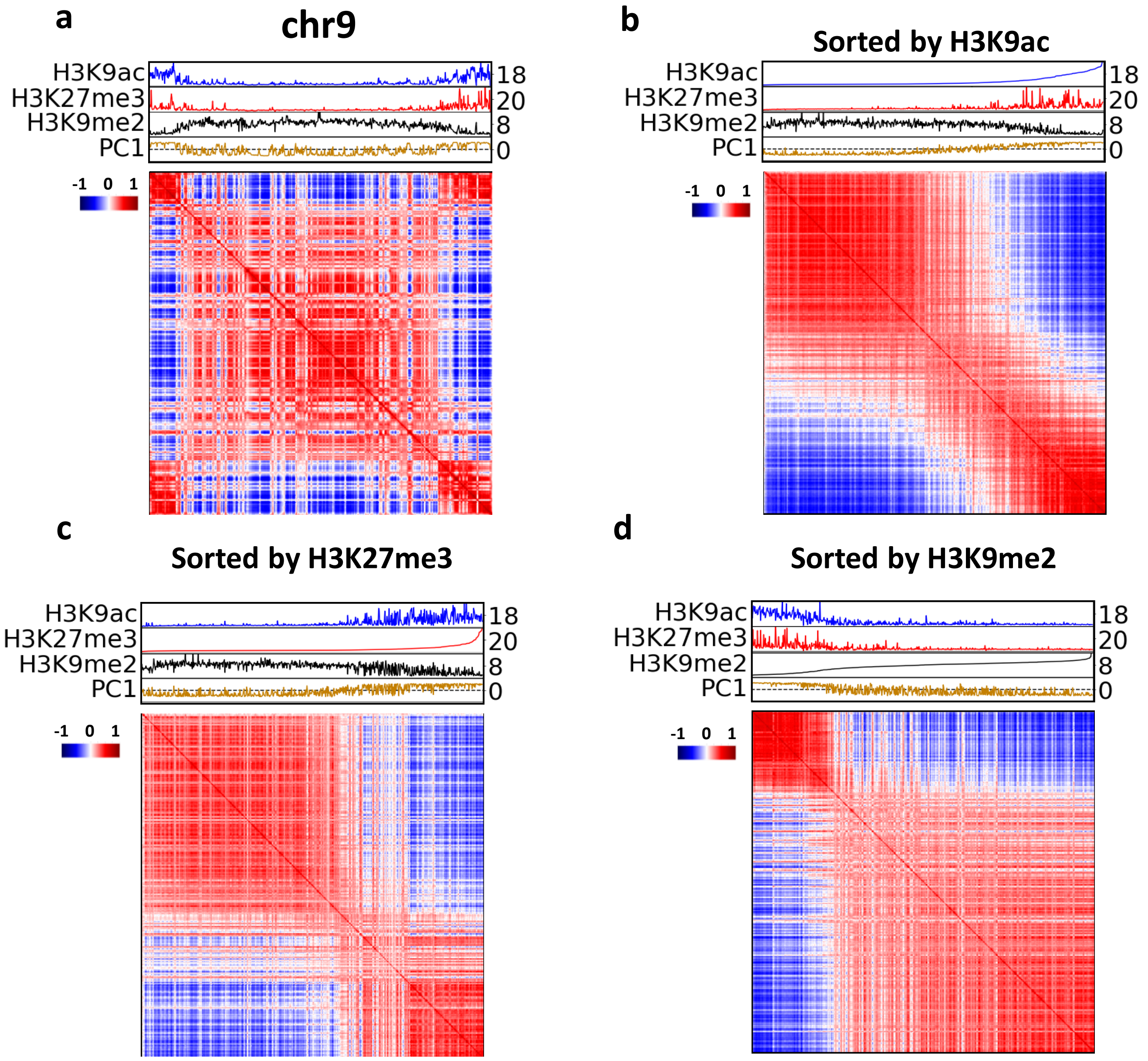

Supplementary Figure 8. Pearson correlations for distance-normalized Hi-C interaction frequency map of chromosomes 1-12. Chromosome 3 is shown in main Figure 2. Shown above each Hi-C map from top to bottom are the following: H3K9ac signal (blue). H3K27me3 signal (red). and H3K9me2 signal (black) and PC1 (brown). For each chromosome from top left to bottom right: (a) Interactions among sequences arranged in natural order. (b) Interactions among sequences sorted by H3K9ac levels. from lowest to highest (c) Interactions among sequences sorted by H3K27me3 levels. from lowest to highest (d) Interactions among sequences sorted by H3K9me2 levels. from lowest to highest.

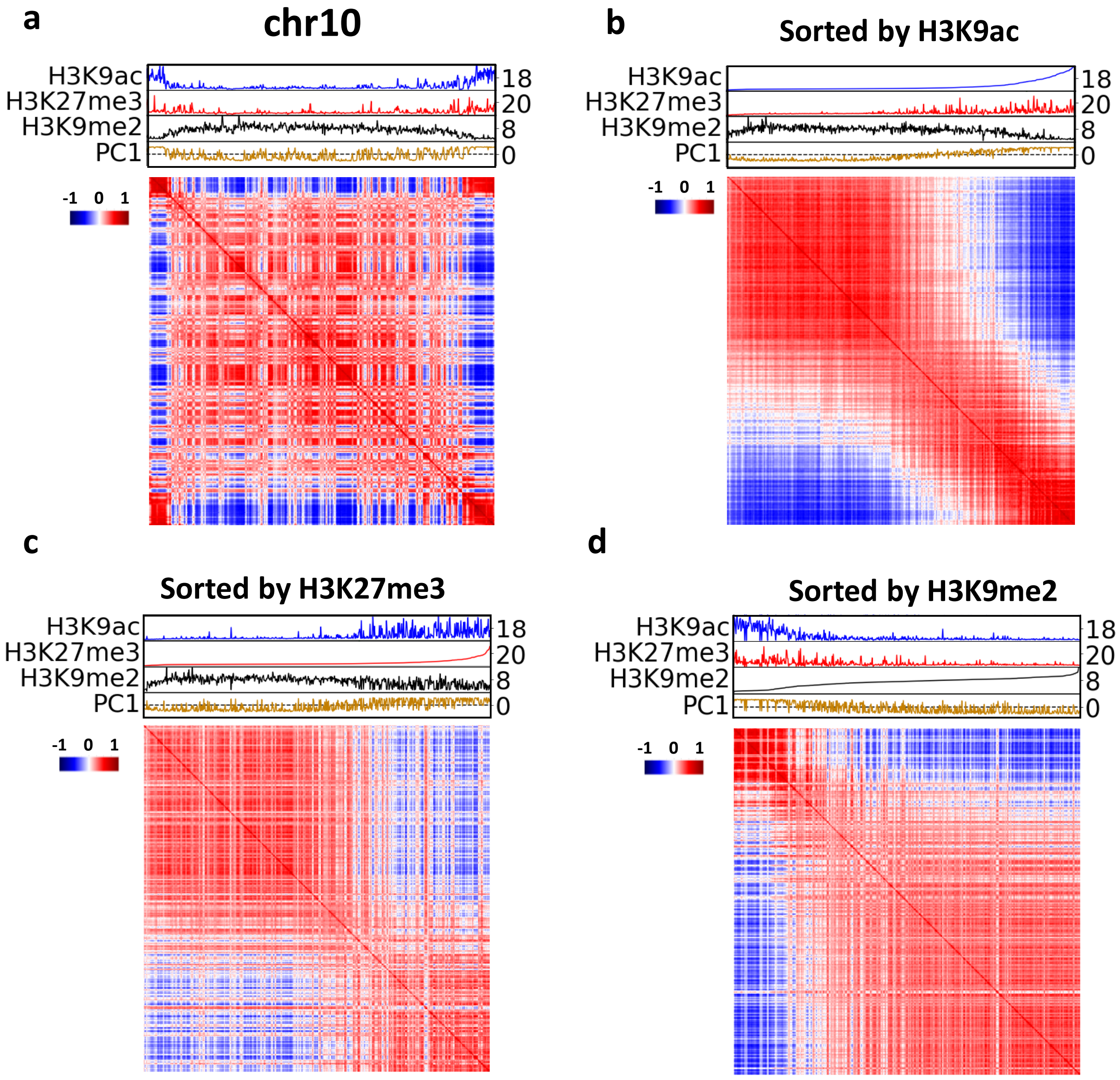

Supplementary Figure 8. Pearson correlations for distance-normalized Hi-C interaction frequency map of chromosomes 1-12. Chromosome 3 is shown in main Figure 2. Shown above each Hi-C map from top to bottom are the following: H3K9ac signal (blue). H3K27me3 signal (red). and H3K9me2 signal (black) and PC1 (brown). For each chromosome from top left to bottom right: (a) Interactions among sequences arranged in natural order. (b) Interactions among sequences sorted by H3K9ac levels. from lowest to highest (c) Interactions among sequences sorted by H3K27me3 levels. from lowest to highest (d) Interactions among sequences sorted by H3K9me2 levels. from lowest to highest.

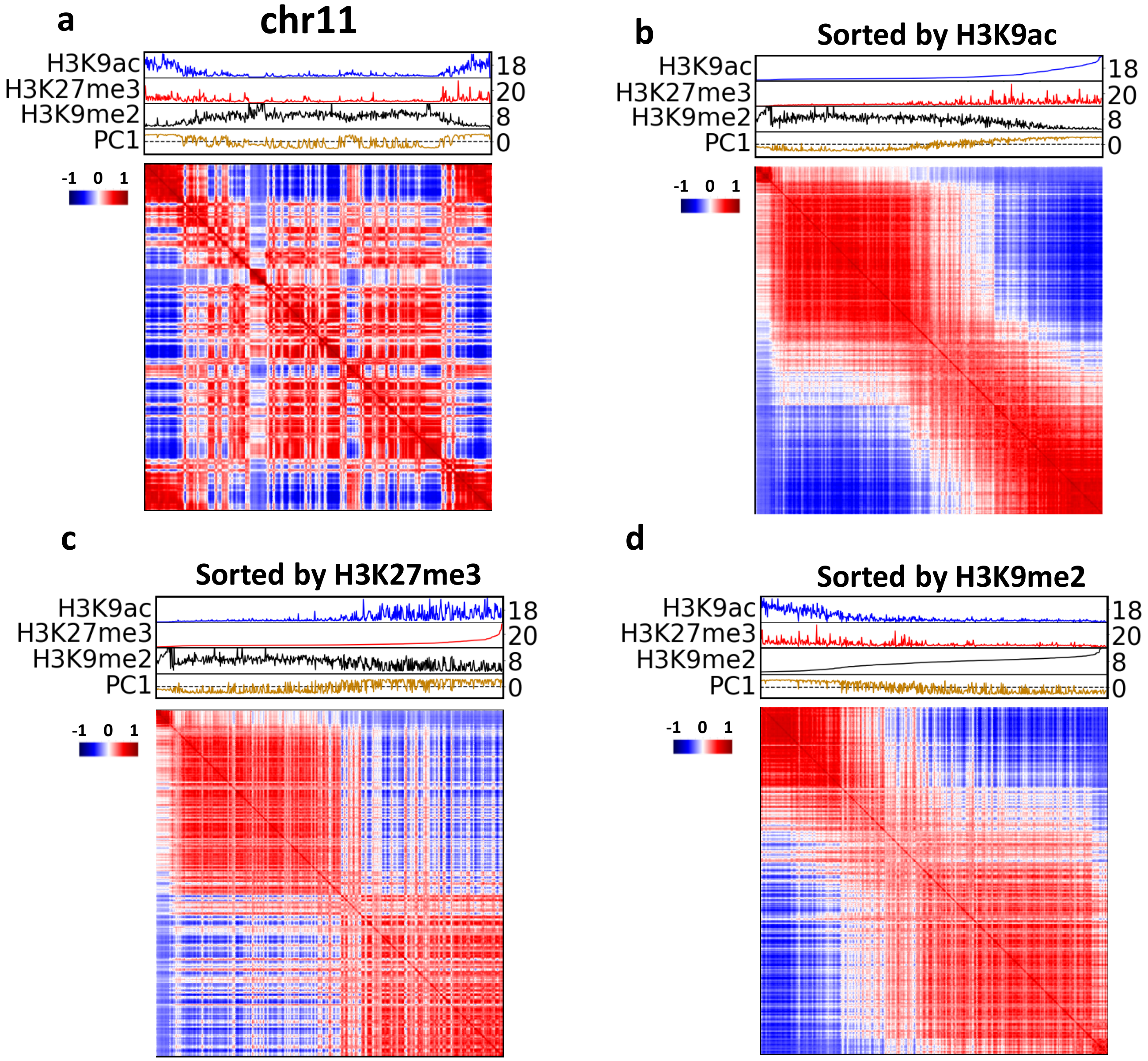

Supplementary Figure 8. Pearson correlations for distance-normalized Hi-C interaction frequency map of chromosomes 1-12. Chromosome 3 is shown in main Figure 2. Shown above each Hi-C map from top to bottom are the following: H3K9ac signal (blue). H3K27me3 signal (red). and H3K9me2 signal (black) and PC1 (brown). For each chromosome from top left to bottom right: (a) Interactions among sequences arranged in natural order. (b) Interactions among sequences sorted by H3K9ac levels. from lowest to highest (c) Interactions among sequences sorted by H3K27me3 levels. from lowest to highest (d) Interactions among sequences sorted by H3K9me2 levels. from lowest to highest.

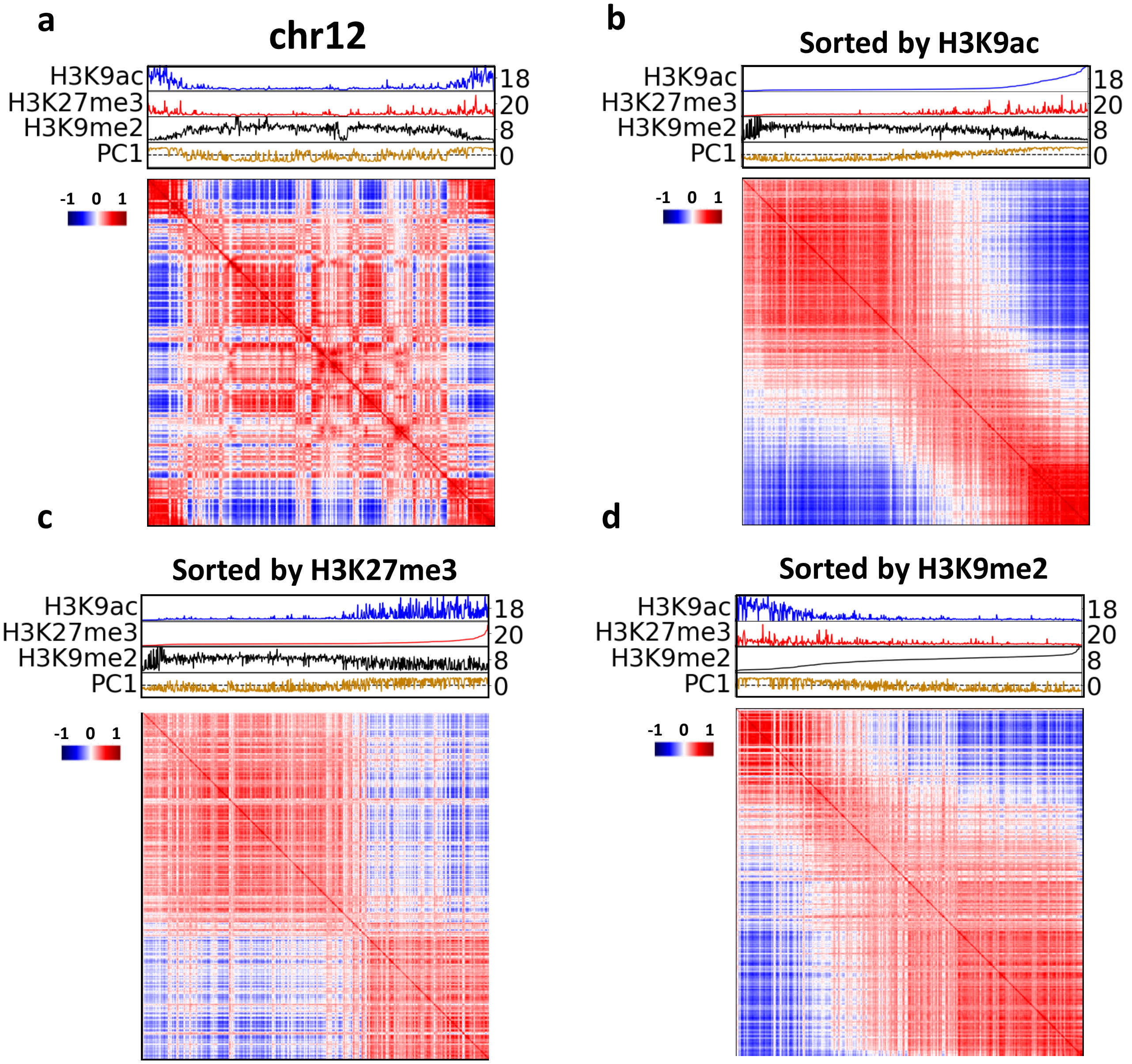

Supplementary Figure 8. Pearson correlations for distance-normalized Hi-C interaction frequency map of chromosomes 1-12. Chromosome 3 is shown in main Figure 2. Shown above each Hi-C map from top to bottom are the following: H3K9ac signal (blue). H3K27me3 signal (red). and H3K9me2 signal (black) and PC1 (brown). For each chromosome from top left to bottom right: (a) Interactions among sequences arranged in natural order. (b) Interactions among sequences sorted by H3K9ac levels. from lowest to highest (c) Interactions among sequences sorted by H3K27me3 levels. from lowest to highest (d) Interactions among sequences sorted by H3K9me2 levels. from lowest to highest.

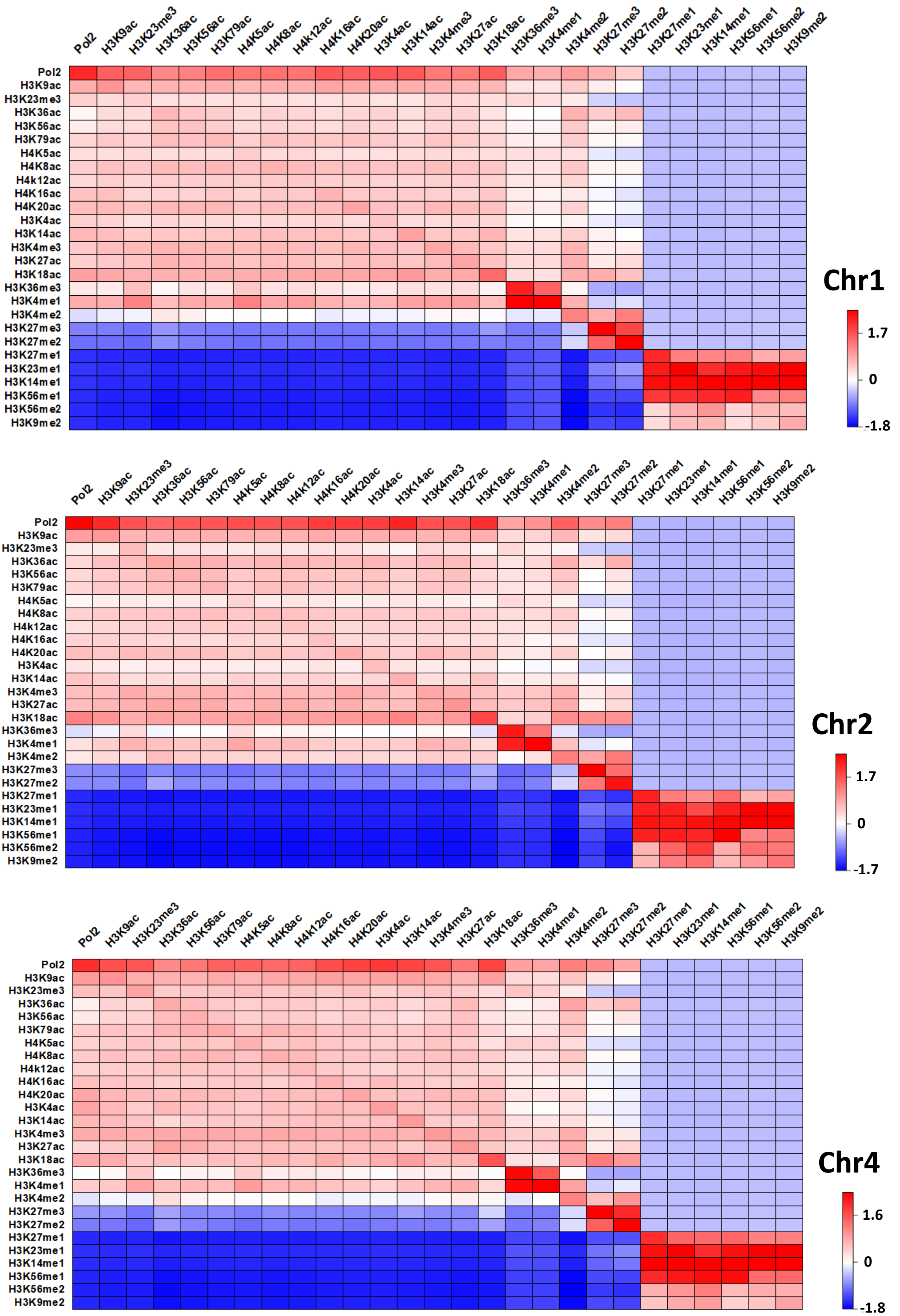

Supplementary Figure 9. Heatmap presenting the logarithms of odds ratios of all combinations of interacting chromatin histone marks by chromosome. Chromosome 3 is shown in main figure 2. Positive log2(odds ratio) indicates enrichment and negative indicates depletion.

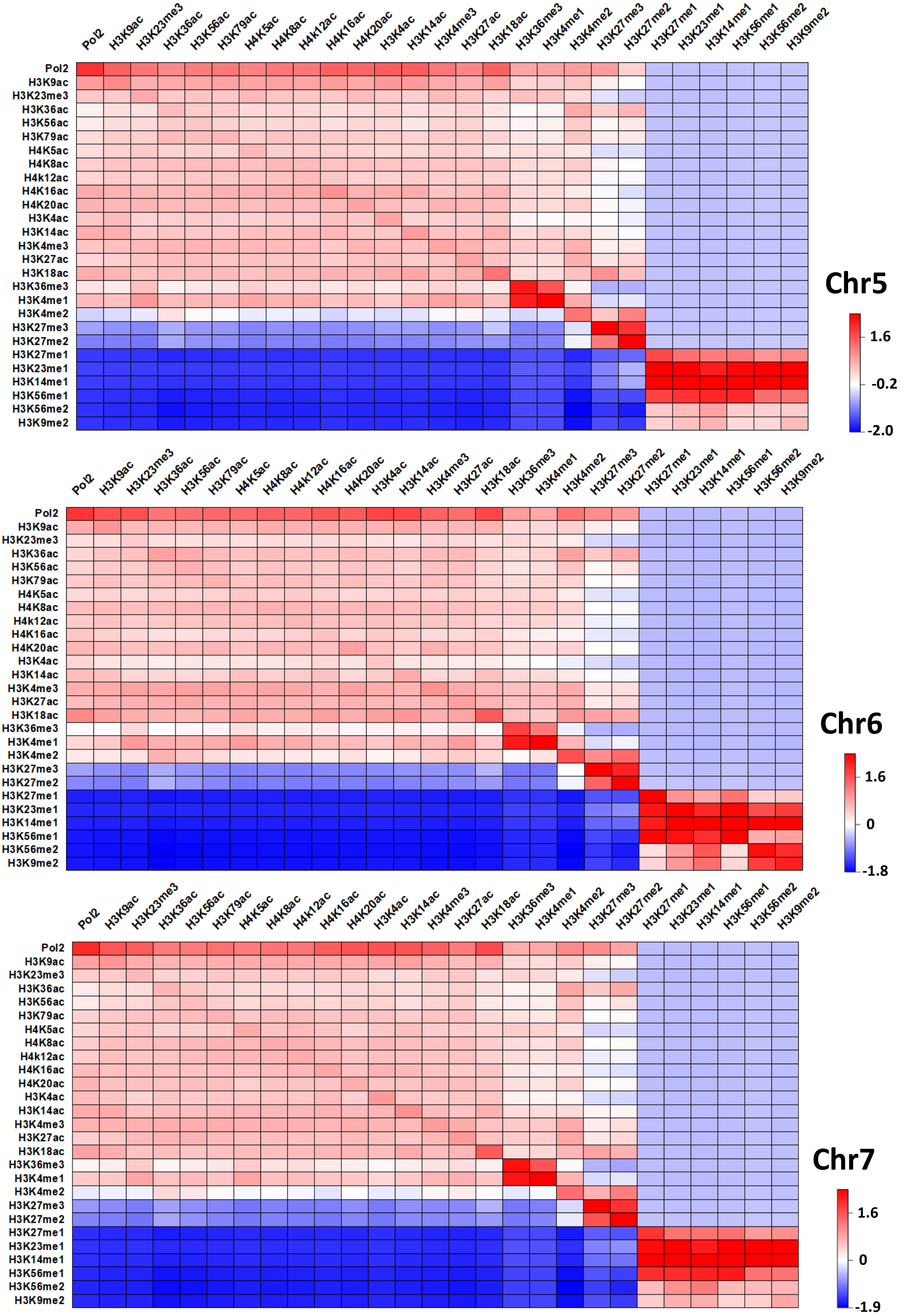

Supplementary Figure 9. Heatmap presenting the logarithms of odds ratios of all combinations of interacting chromatin histone marks by chromosome. Chromosome 3 is shown in main figure 2. Positive log2(odds ratio) indicates enrichment and negative indicates depletion.

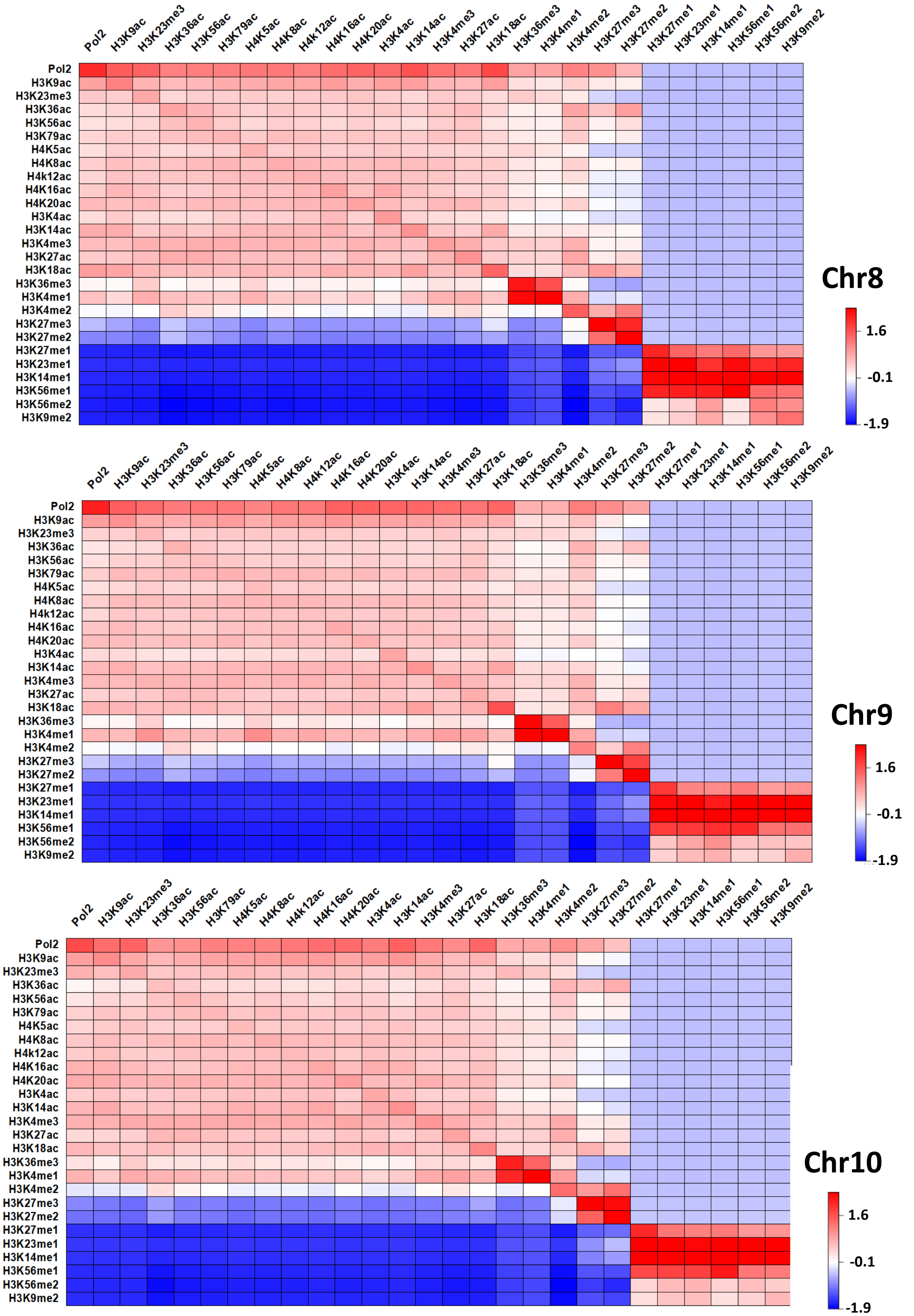

Supplementary Figure 9. Heatmap presenting the logarithms of odds ratios of all combinations of interacting chromatin histone marks by chromosome. Chromosome 3 is shown in main figure 2. Positive log2(odds ratio) indicates enrichment and negative indicates depletion.

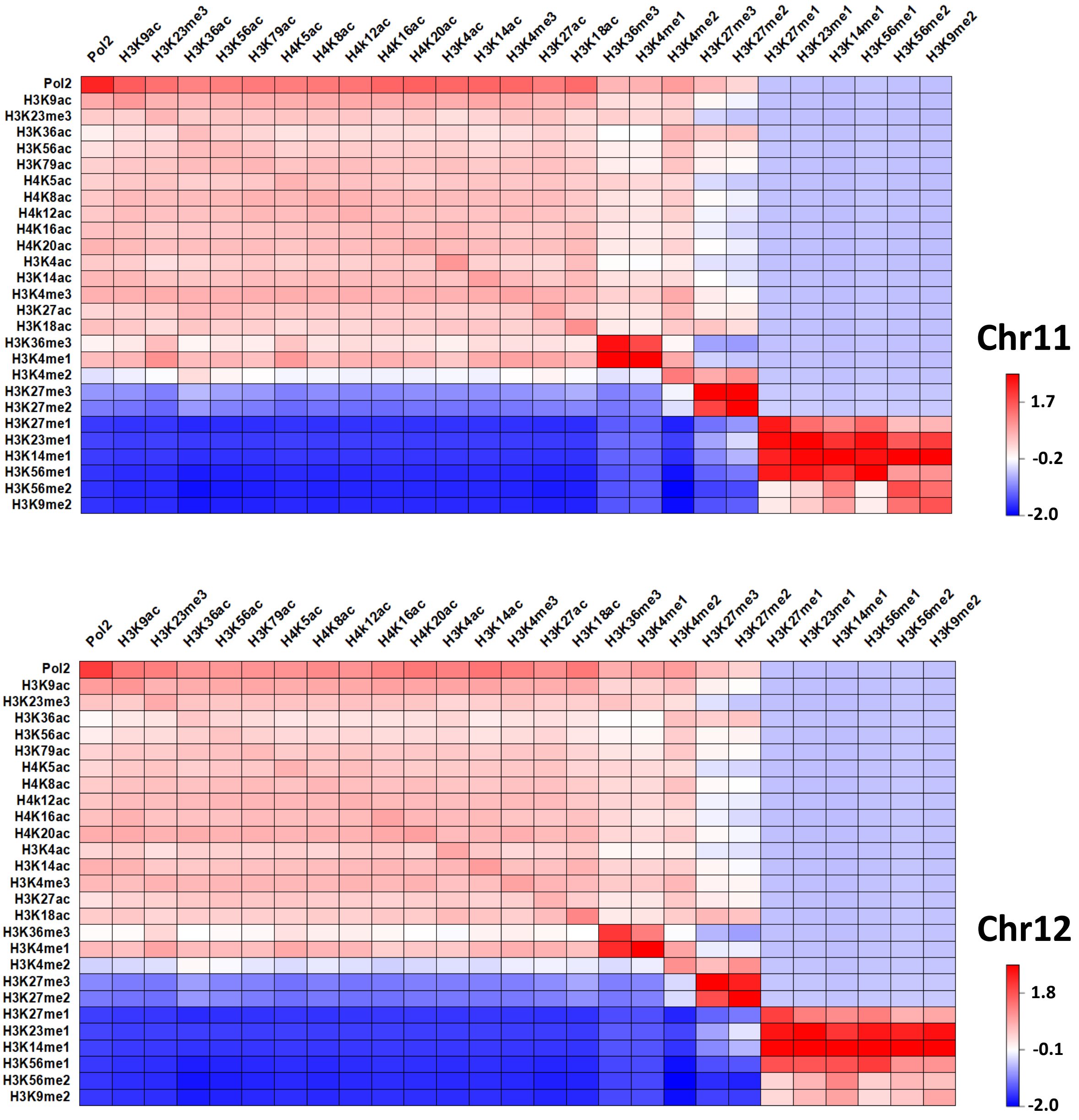

Supplementary Figure 9. Heatmap presenting the logarithms of odds ratios of all combinations of interacting chromatin histone marks by chromosome. Chromosome 3 is shown in main figure 2. Positive log2(odds ratio) indicates enrichment and negative indicates depletion.

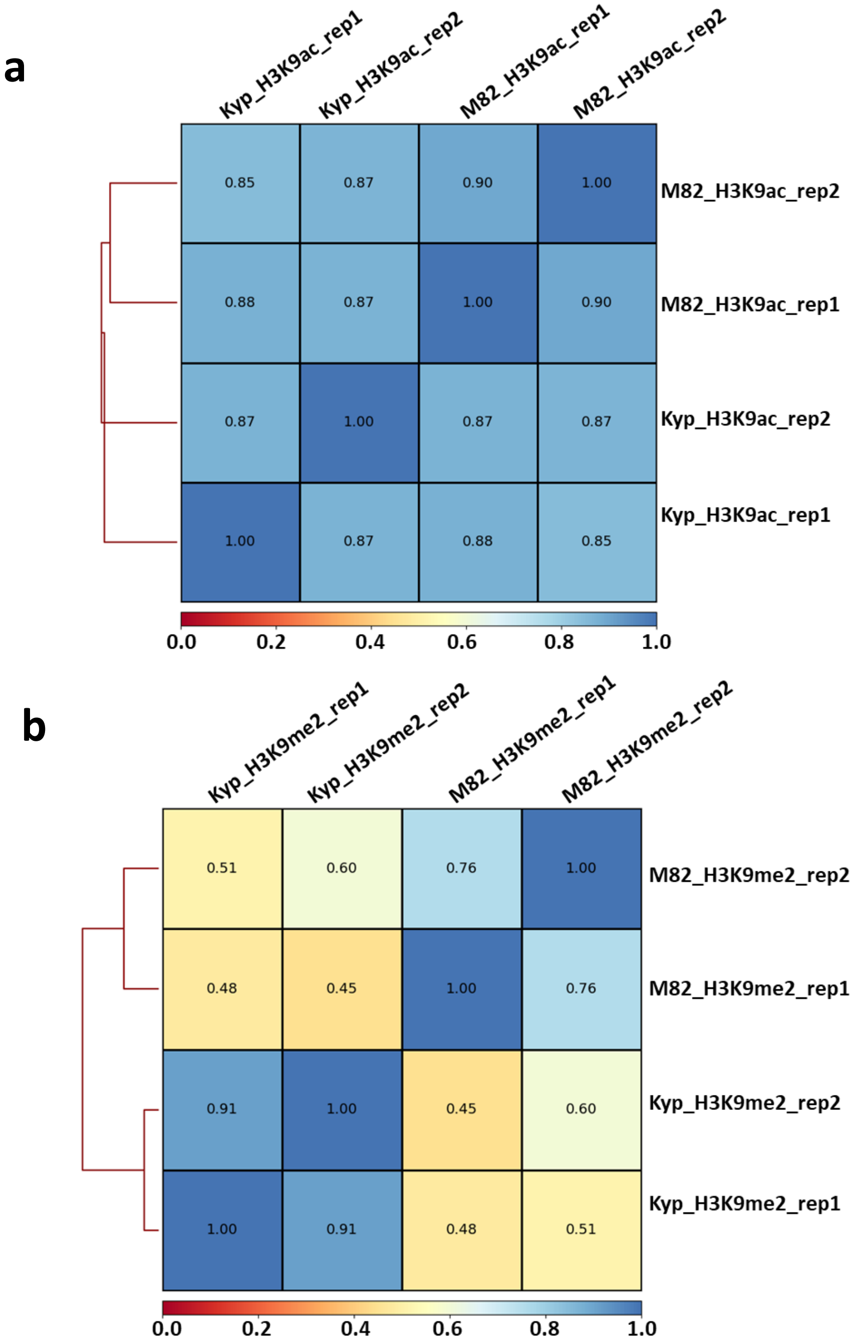

Supplementary Figure 10. (a) Pearson correlation heatmaps of ChIP-seq (H3K9ac) (b) Pearson correlation heatmaps of ChIP-seq (H3K9me2)

Motif	P-value
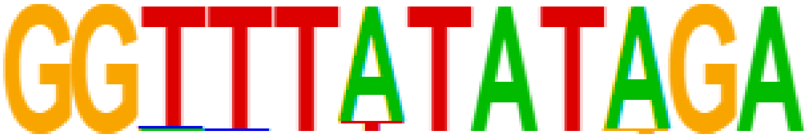	1e-62

Supplementary Figure 11. TBP binding motif (GGTTTATATAGA), determined by HOMER enrichment analysis.

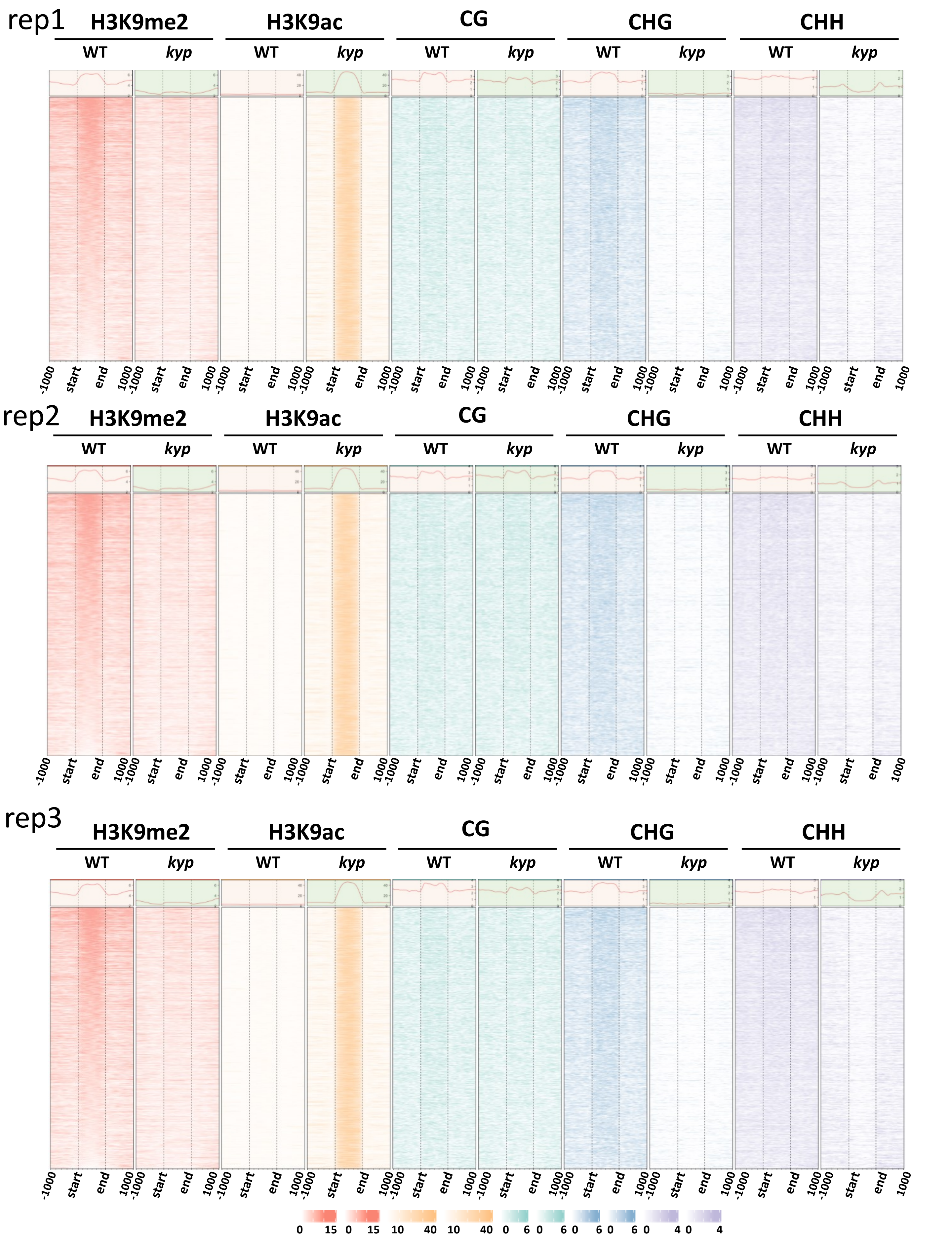

Supplementary Figure 12. Heatmaps showing the distribution of H3K9me2. H3K9ac and DNA methylation signals on genomic regions that gain H3K9ac in *kyp* (n = 1974). Data presented based on individual biological replicates.

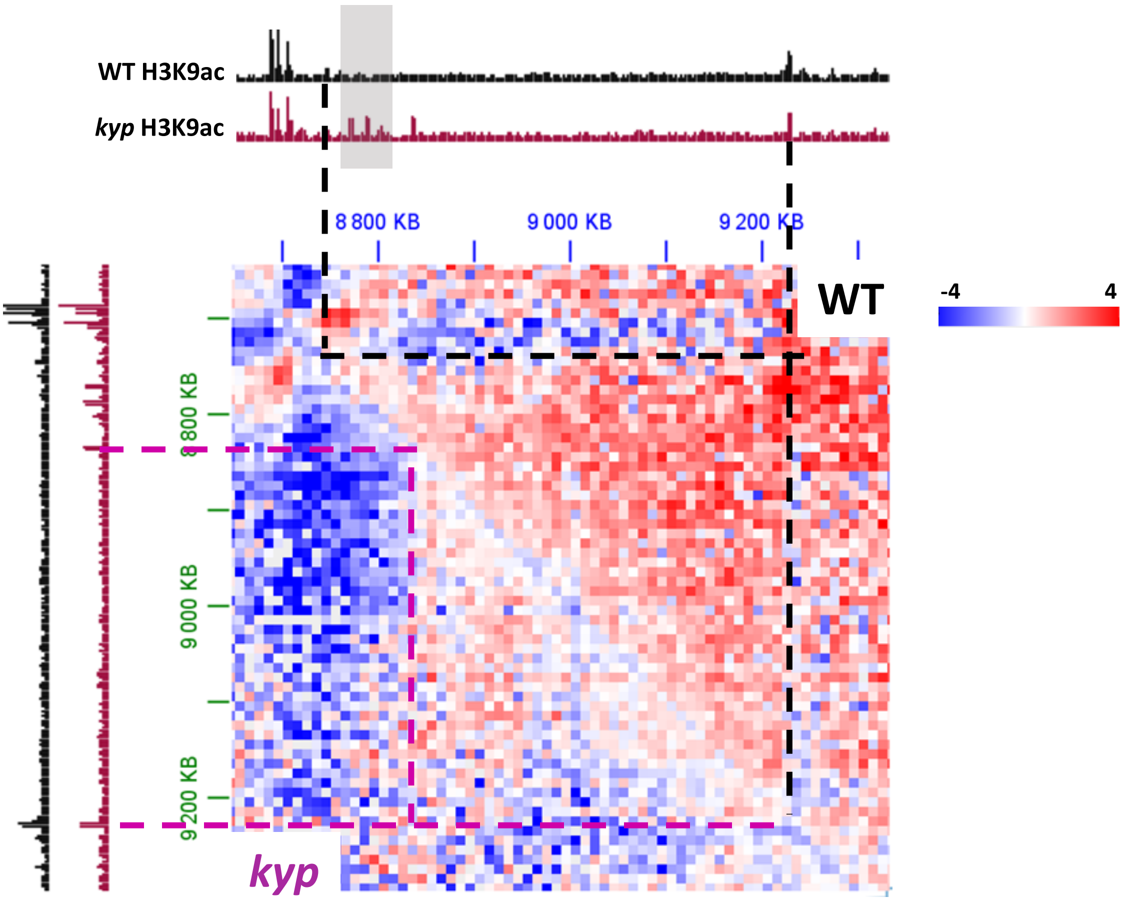

Supplementary Figure 13. Integration of Hi-C and ChIP-seq, a 2D heatmap with the interaction frequency in region chr1:8650kb-9320kb. Red dots represent enriched interactions, and blue dots depleted interactions. Additionally, the Hi-C Heatmap comparing WT and *kyp* contacts is illustrated, with dashed lines showing the boundaries of TAD-like structures that correlate with acetylation.

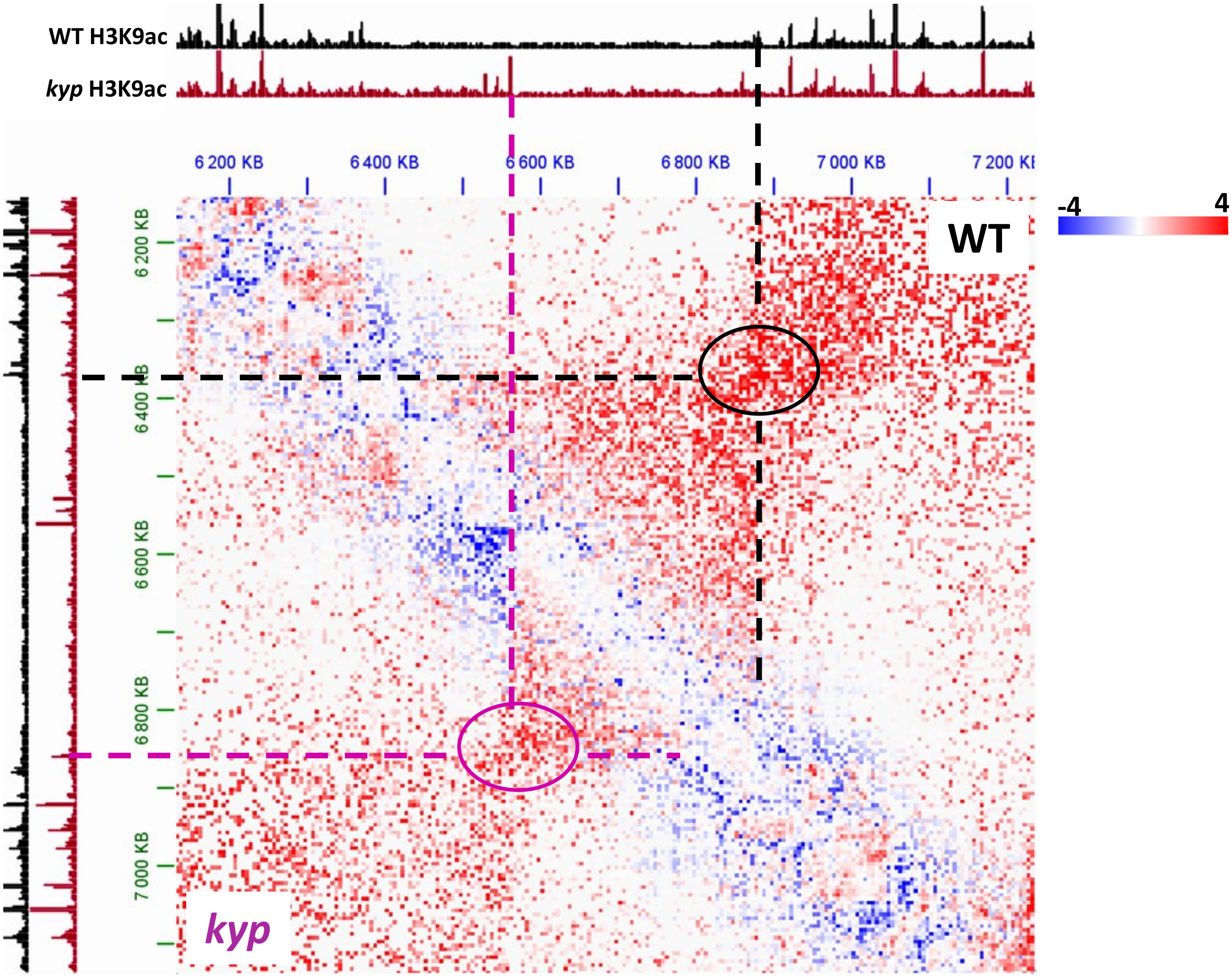

Supplementary Figure 14. Integration of Hi-C and ChIP-seq, a 2D heatmap with the interaction frequency in region chr9:6.15-7.2Mb. Red dots represent enriched interactions, and blue dots depleted interactions. Additionally, the Hi-C Heatmap comparing WT and *kyp* contacts is illustrated, with dashed lines showing the boundaries of TAD-like structures that correlate with acetylation.

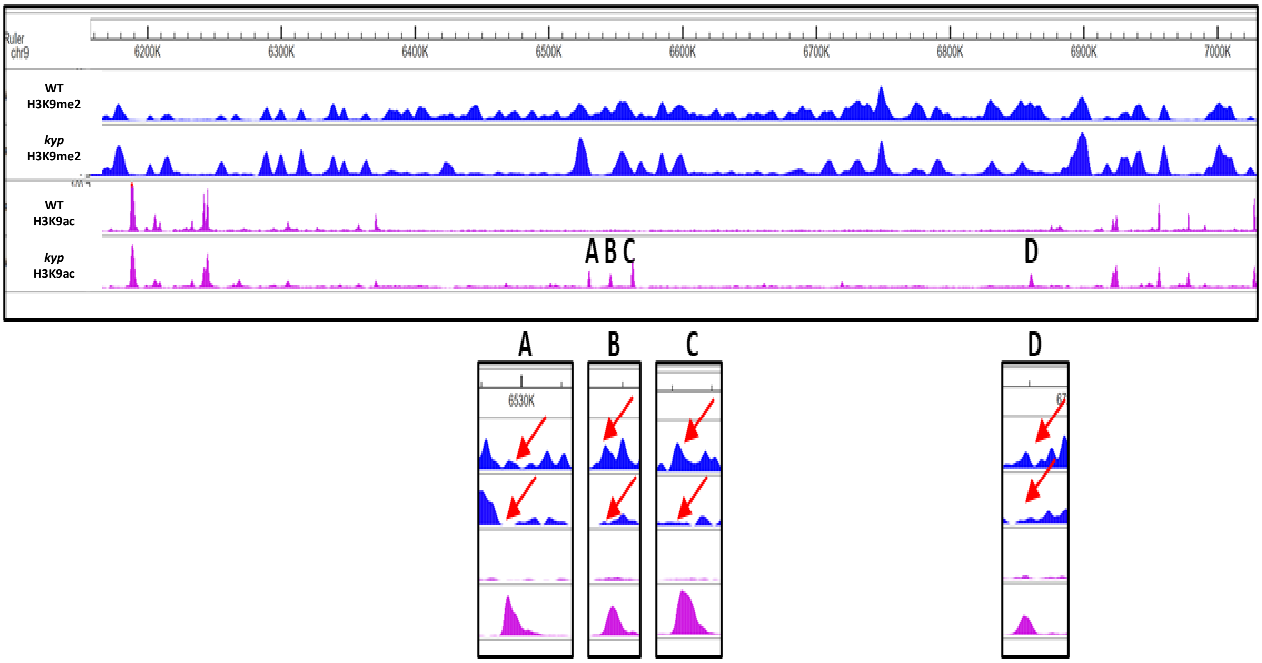

Supplementary Figure 15. Zoom-in region presented in figure 3C (chr9:6.15-7Mb). Red arrows highlight regions losing H3K9me2 and gaining H3K9ac. Scale 0-36 for H3K9me2 and 0-100 for H3K9ac.

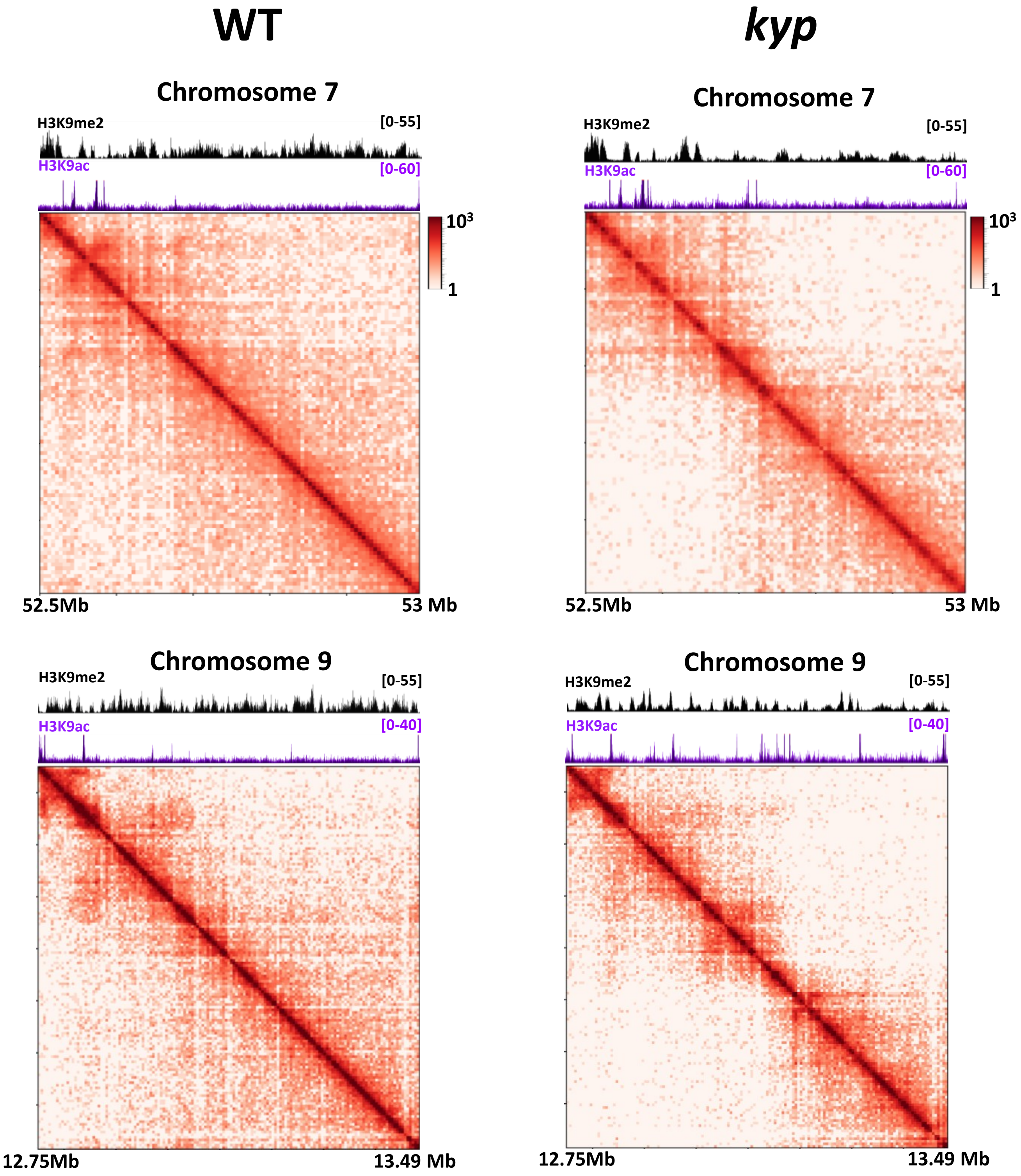

Supplementary Figure 16. Examples of TAD-like structures rearrangements. Heatmaps showing the contact matrices over genomic loci that display a switch from H3K9me2 to H3K9ac in *kyp.* Histone modification ChIP-seq tracks from WT and *kyp* are also shown.

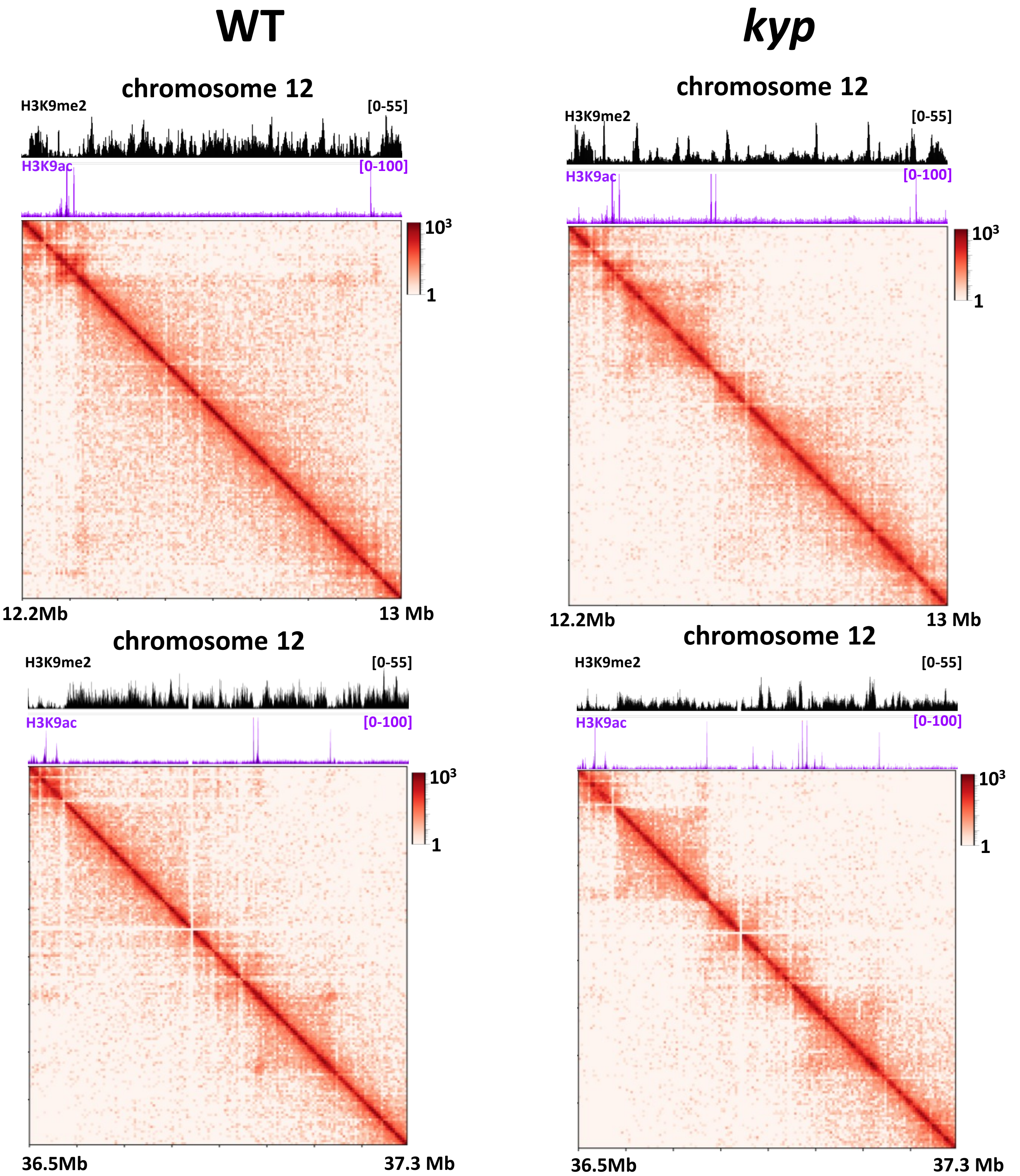

Supplementary Figure 16. Examples of TAD-like structures rearrangements. Heatmaps showing the contact matrices over genomic loci that display a switch from H3K9me2 to H3K9ac in *kyp.* Histone modification ChIP-seq tracks from WT and *kyp* are also shown.

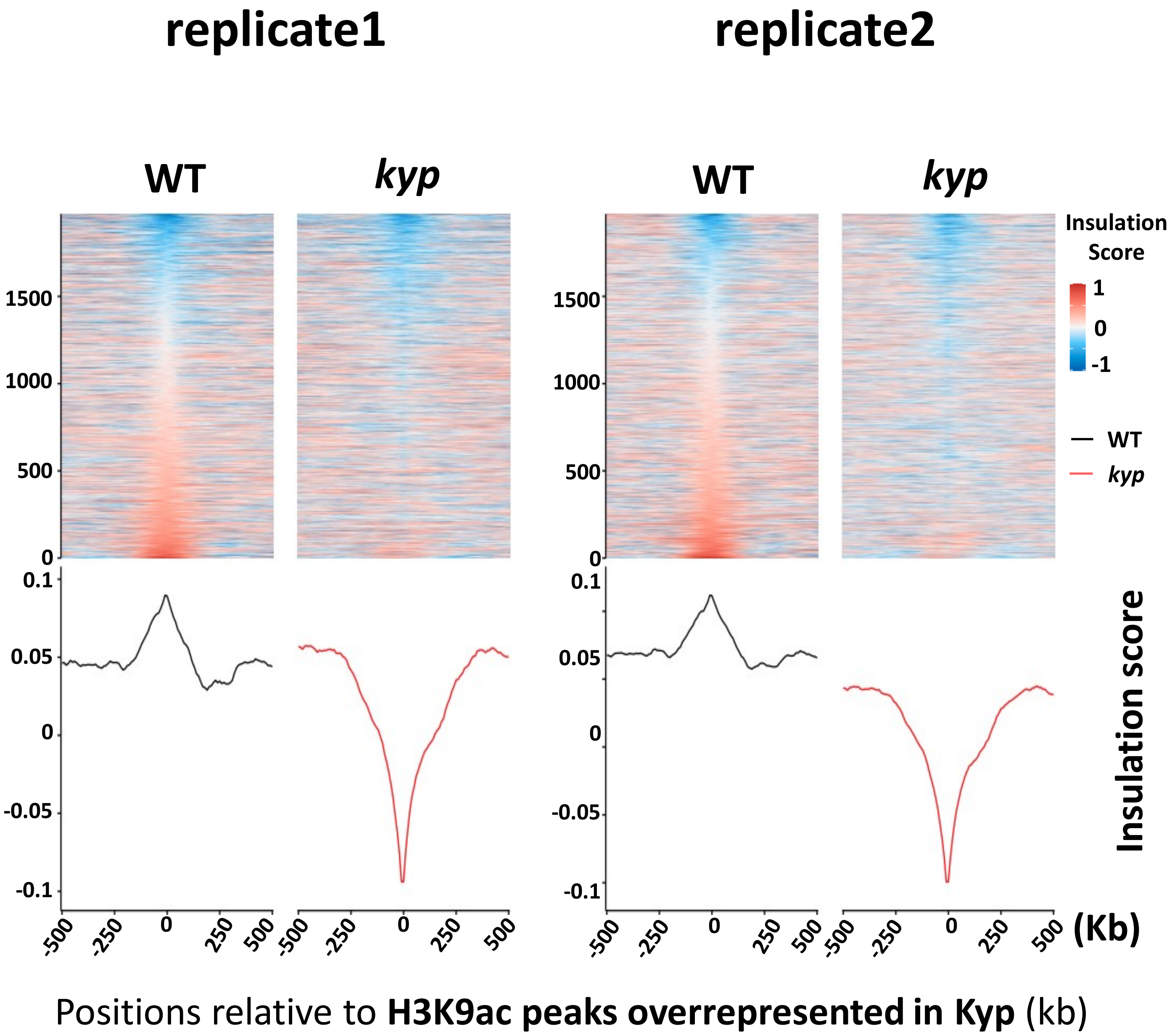

Supplementary Figure 17. Heatmaps (top) and average insulation scores (bottom) around H3K9ac peaks overrepresented in *kyp* within ±500 Kb. The insulation scores at H3K9ac peaks were reduced in *kyp*. The black line represents the WT insulation score and red line represent *kyp* insulation score.

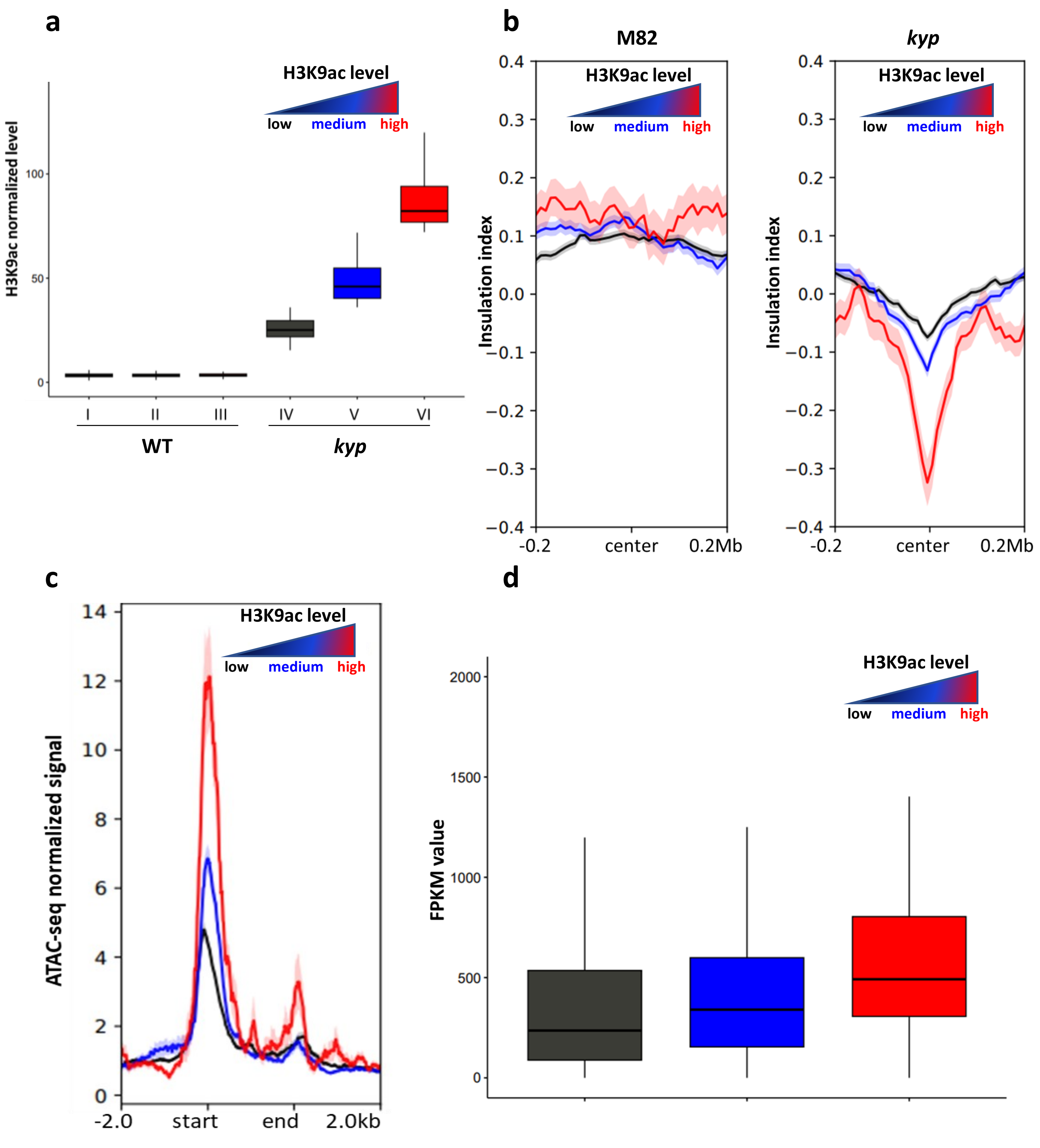

Supplementary Figure 18. (a) Ectopic gained H3K9ac enrichment levels in *kyp* classified from low to high as shown in IV, V and VI groups. Corresponding regions in the WT are presented in groups I, II and III; showing their related H3K9ac levels. (b) Plots displaying the average insulation index around H3K9ac peaks classified into three categories (low in black, medium in blue, and high in red) overrepresented in M82 and *kyp* within ±0.2 Mb. (c) Metaplot showing the normalized ATAC-seq read density in kyp over the region that gain H3K9ac categorized from low to high level of H3K9ac. (d) Average expression levels of the transcripts associated to the three H3K9ac categories in *kyp*.

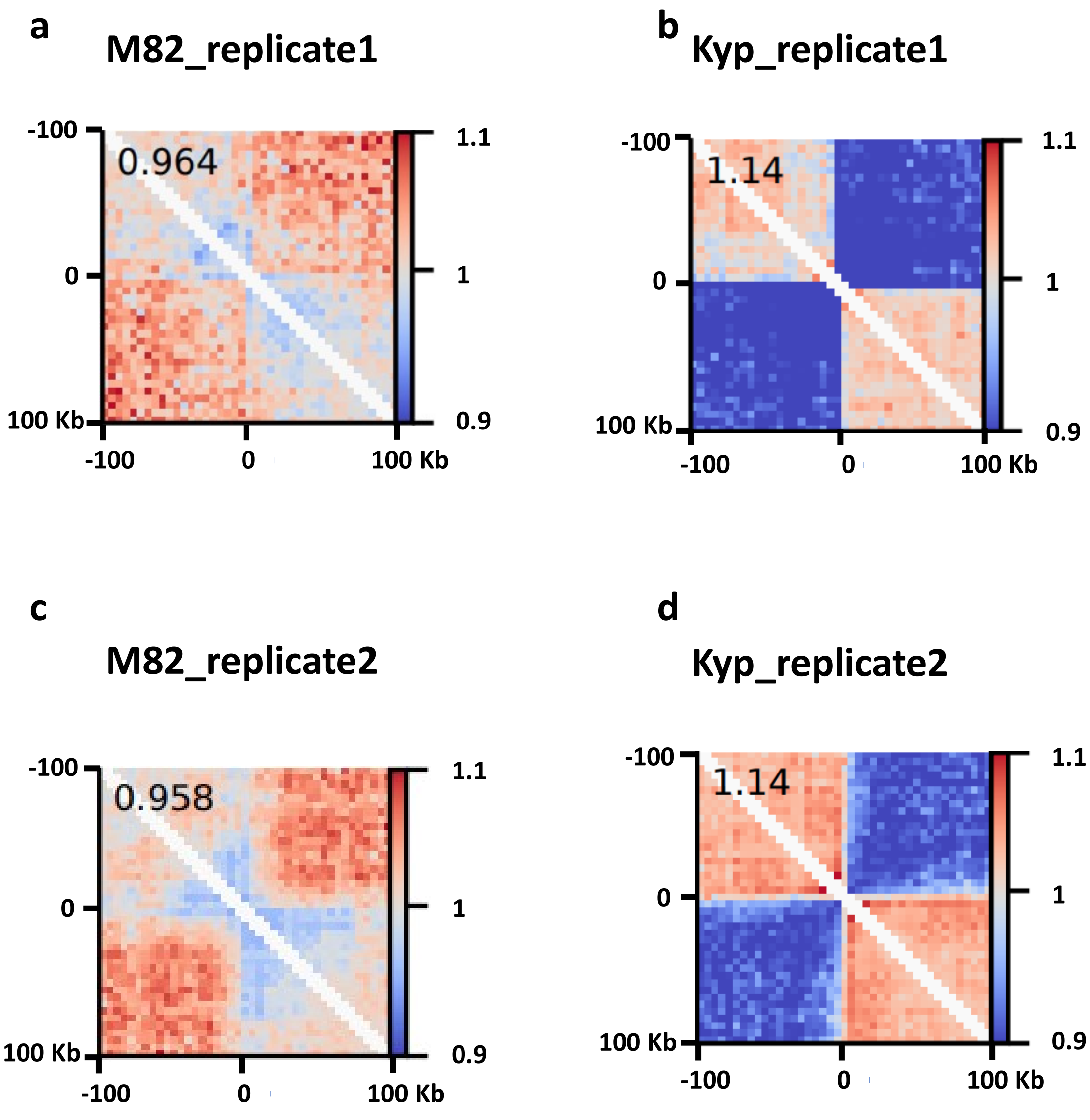

Supplementary Figure 19. Local Pile-up analysis of chromatin architecture surrounding H3K9ac peaks overrepresented in *kyp*. The 0 indicates H3K9ac enriched regions in *kyp* compared to WT. Relative insulation strength is shown in the left top corner of the plots. Distance from peak region is 100Kb. Color range corresponds to the log-scaled observed/expected contact frequency. (a. c) WT replicates 1 and 2. (b. d) *kyp* replicates 1 and 2. The actual value for each replicate is displayed on top left.

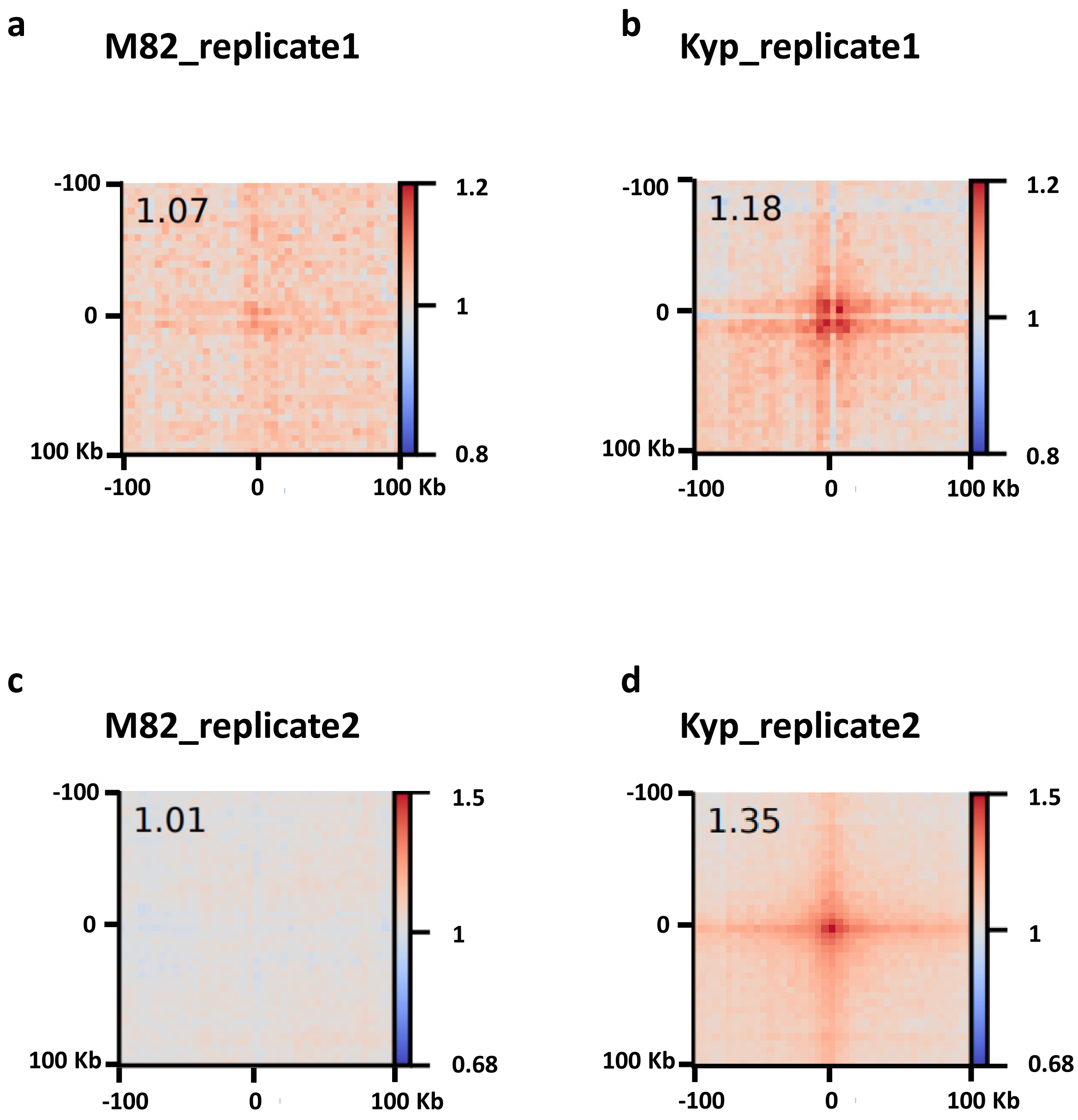

Supplementary Figure 20. Pile-ups of interactions between genomic loci that display a switch from H3K9me2 to H3K9ac in *kyp*. The 0 indicates H3K9ac enriched regions in *kyp* compared with WT. Distance from peak region is 100Kb. Color range corresponds to the log-scaled observed/expected contact frequency. Enrichment score is shown in the left top corner of the plots.

Supplementary Table 1. Antibodies used for immuniostaining and ChIP-Seq analyses.

Antibodies for Immunostaining	Antibodies for ChIPseq
RNAPII (Abcam. ab26721)	RNAPII (Abcam. ab26721)
H3K4ac (Active Motif. 39382)	H3K4ac (Active Motif. 39382)
H3K4me3 (Active Motif. 39157)	H3K4me3 (Millipore 07-473)
H3K4me2 (Active Motif. 39142)	H3K4me2 (Active Motif. 39142)
H3K4me1 (Active Motif. 39300)	H3K4me1 (Active Motif. 39300)
H3K9ac (Millipore. 07-352)	H3K9ac (Millipore. 07-352)
H3K9me2 (Abcam. ab1220)	H3K9me2 (Abcam. ab1220)
H3K14ac (Millipore. 07-353)	H3K14ac (Millipore. 07-353)
H3K14me1 (Active Motif. 61510)	H3K14me1 (Active Motif. 61510)
H3K18ac (Millipore. 07-354)	H3K18ac (Millipore. 07-354)
H3K23me3 (Active Motif. 61500)	H3K23me3 (Active Motif. 61500)
H3K23me1 (Active Motif. 39388)	H3K23me1 (Active Motif. 39388)
H3K27ac (Abcam. ab4729)	H3K27ac (Abcam. ab4729)
H3K27me3 (Active Motif. 39157)	H3K27me3 (Millipore 07-449)
H3K27me2 (Active Motif. 39246)	H3K27me2 (Abcam. 24684)
H3K27me1 (Millipore. 07-488)	H3K27me1 (Millipore. 07-488)
H3K36ac (Active Motif. 39380)	H3K36ac (Active Motif. 39380)
H3K36me3 (Abcam. ab9050)	H3K36me3 (Abcam. ab9050)
H3K56me2 (Active Motif. 39278)	H3K56me2 (Active Motif. 39278)
H3K56me1 (Active Motif. 39274)	H3K56me1 (Active Motif. 39274)
H3K79ac (Active Motif. 39566)	H3K79ac (Active Motif. 39566)
H4K5ac (Active Motif. 39700)	H4K5ac (Active Motif. 39700)
H4K8ac (Active Motif. 61104)	H4K8ac (Active Motif. 61104)
H4K12ac (Active Motif. 61531)	H4K12ac (Active Motif. 61531)
H4K16ac (Active Motif. 39930)	H4K16ac (Active Motif. 39930)
H4K20ac (Active Motif. 61531)	H4K20ac (Active Motif. 61531)

Supplementary Table 2. Mapping statistics for hic libraries.

	HiC_M82_rep1		HiC_kyp_rep1		HiC_M82_rep2		HiC_kyp_rep2	
Total_pairs_processed	608269392	100.00	484313159	100.0	334842370	100.0	236015040	100.0
Unmapped_pairs	4057094	0.67	3071686	0.63	843664	0.25	604367	0.26
Low_qual_pairs	0	0.0	0	0.0	0	0.0	0	0.0
Unique_paired_alignments	446761946	73.45	327612330	67.65	237908609	71.05	157268943	66.64
Multiple_pairs_alignments	138798588	22.82	132690276	27.40	85431316	25.51	70100143	29.70
Pairs_with_singleton	18651764	3.07	20938867	4.32	10658781	3.18	8041587	3.41
Low_qual_singleton	0	0.0	0	0.0	0	0.0	0	0.0
Unique_singleton_alignments	0	0.0	0	0.0	0	0.0	0	0.0
Multiple_singleton_alignments	0	0.0	0	0.0	0	0.0	0	0.0
Reported_pairs	446761946	73.45	327612330	67.65	237908609	71.05	157268943	66.64
valid_pairs	104665004	0.17	112722633	0.23	169512103	0.506	111884859	0.47
trans_interaction	34878158		29383231		24048706		15657048	
cis_interaction	69786846		83339402		145463397	6.05	96227811	6.15
cis_shortRange	33936805		27829374		61857354	0.43	38201614	0.40
cis_longRange	35850041		55510028		83606043	0.58	58026197	0.60
